# Phytotoxic Metabolites from *Hyptis* Species: Chemical Profiling and Bioherbicide Activity Against *Amaranthus*

**DOI:** 10.1021/acsomega.5c07637

**Published:** 2025-10-18

**Authors:** Mariana A. Silva, Letícia P. F. da Silva, Karen J. Nicácio, Barbara S. Bellete, Lucas C. C. Vieira, Olívia M. Sampaio

**Affiliations:** † Chemistry Department, Federal University of Mato Grosso, 78060-900 Cuiabá, MT, Brazil; ‡ Chemistry Department, Federal University of Lavras, 37200-900 Lavras, MG, Brazil

## Abstract

The phytotoxic effects of extracts from *Hyptis crenata* (***Hcre***), *Hyptis suaveolens* (***Hsua***), *Hyptis saxatilis* (***Hsax***), *Hyptis campestris* (***Hcam***), and *Hyptis brevipes* (***Hbre***) were evaluated against three *Amaranthus* weed species under controlled germination chamber conditions. Extracts ***Hcre***, ***Hsua*** and ***Hsax*** inhibited seed germination by 85–93% relative to the control and markedly suppressed radicle and hypocotyl growth in *Amaranthus viridis* and *Amaranthus hybridus* in Petri dish assays. At 200 ppm, ***Hcre*** and ***Hsax*** reduced radicle and hypocotyl lengths by up to 90% in both species, comparable to the effects of commercial herbicides Sencor and Sulfentrazone. Multivariate analyses, including PCA, PLS-DA, and molecular networking based on LC-MS/MS data, enabled the annotation of compounds associated with phytotoxicity. A total of 56 metabolites were identified, including terpenoids, flavonoids, α-pyrones, carbohydrate derivatives, and polyketides, using both an in-house database and the GNPS platform. Terpenoids were the predominant class, with abietane-type diterpenes as the most abundant subgroup, exhibiting strong correlations with the observed biological activity.

## Graphical abstract

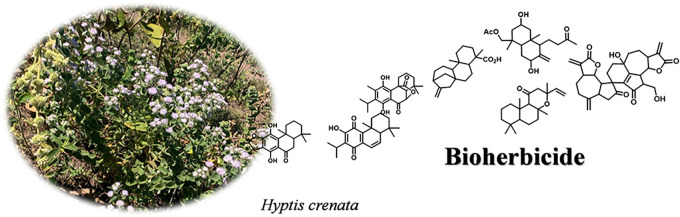

## Introduction

Agrochemical development draws from diverse sources of bioactive compounds, ranging from the phytotoxic metabolites found in nature to synthetically engineered molecules which are designed for targeted activity and reduced environmental toxicity.^1,2^ Traditionally, research into the diverse array of bioactive metabolites produced by the plants have showed a significant potential, encompassing applications such as bioherbicides development, immune modulation, pharmacological activities, and the enhancement of the abiotic stress tolerance.^3–5^ The control of invasive plants remains a major challenge in agricultural production.^6^ Integrated weed management, particularly the use of bioherbicides, has emerged as a promising strategy for sustainable agriculture, offering both ecological and economic benefits.^7^ Bioherbicides derived from natural sources, such as plant and microbial extracts, have demonstrated potential for pre- and postemergence weed control by inhibiting seed germination and plant growth.^8^

Seed germination, a critical stage in plant development, involves essential physiological and biochemical processes necessary for seedling establishment.^9^ Plant extracts used as bioherbicides can suppress germination by blocking nutrient hydrolysis and disrupting cell division,^10^ thereby chemically regulating weed development. These extracts are rich in secondary metabolites that act as phytotoxins, inhibiting weed growth by interfering with processes such as transpiration, respiration, and photosynthesis.^11^ This has driven interest in plant species with allelopathic potential as sources for novel bioherbicides.^12^

The genus *Hyptis* (Lamiaceae), comprising approximately 400 species primarily distributed across tropical America, has attracted considerable attention due to its chemical diversity.^13^ Species in this genus produce a wide array of bioactive compounds, including flavonoids,^14^ terpenes and steroids,^15^ phenolic acids,^16^ alkaloids,^17^ lignans,^18^ and other secondary metabolites..^19,20^ Previous studies have reported the phytotoxic activity of *Hyptis* extracts and isolated metabolites. For example, extracts from *Hyptis suaveolens* and *Hyptis rhomboidea* inhibited germination and seedling growth of several crop and weed species, including *Brassica campestris*, *Raphanus sativus L*., *Oryza sativa*, *Lactuca sativa*, *Lolium multiflorum*, and *Echinochloa crus-galli*.^21^ Additionally, suaveolic acid, an allelochemical isolated from *H*. *suaveolens*, reduced the growth of *L*. *sativa*, *L*. *multiflorum*, and *E*. *crus-galli*.^22^

Metabolomics has increasingly been applied to investigate the phytotoxicity of plant extracts, providing insights into their chemical composition and enabling the identification of secondary metabolites responsible for bioactivity. For instance, allelochemicals such as afzelin derivatives, anthraquinones, and phenolic acids were linked to the phytotoxic activity of three *Cassia* species against Chenopodium murale.^23^ Similarly, *Jatropha gossypiifolia* L. inhibited the germination and growth of *Bidens bipinnata* L. with activity attributed to bioactive alkaloids, phenolics, and terpenoids.^24^ Moreover, metabolomic analyses of *Dipteryx lacunifera* Ducke, *Ricinus communis* L., *Piper tuberculatum* Jacq., and *J*. *gossypiifolia* L. identified phenolics and terpenoids as allelochemicals contributing to their allelopathic effects.^25^

This study extends beyond previous research by comparatively investigating the phytotoxic and allelopathic activities of five Hyptis species, Hyptis crenata, H. suaveolens, Hyptis saxatilis, Hyptis campestris, and Hyptis brevipes, against Amaranthus weeds, a globally relevant and herbicide-resistant genus. By integrating bioassays with UPLC-qTOF-MS/MS metabolic profiling and molecular networking via GNPS, we provide a comparative chemical–biological analysis within Hyptis. This integrative approach not only identifies bioactive metabolites but also highlights interspecific variation in allelopathic potential, offering new insights into the discovery of plant-derived bioherbicides.

## Results and Discussion

### Phytotoxicity Assays

Phytotoxic activity was assessed using both seed germination and seedling growth assays to evaluate the bioherbicidal potential of *Hyptis* extracts against three weed species: *Amaranthus viridis*, *Amaranthus hybridus* and *Amaranthus lividus*. Statistical analysis indicated significant inhibitory effects (*p* ≤ 0.05), with the magnitude of inhibition varying by species and growth parameter. While all five *Hyptis* extracts exhibited biological activity; three demonstrated particularly consistent inhibition, reducing germination by more than 80% at 200 ppm relative to the controls. Two commercial herbicides, Sencor (***Senc***) and Sulfentrazone (***Sulf***), were included as reference standards (Figure 1).

**Figure 1:**
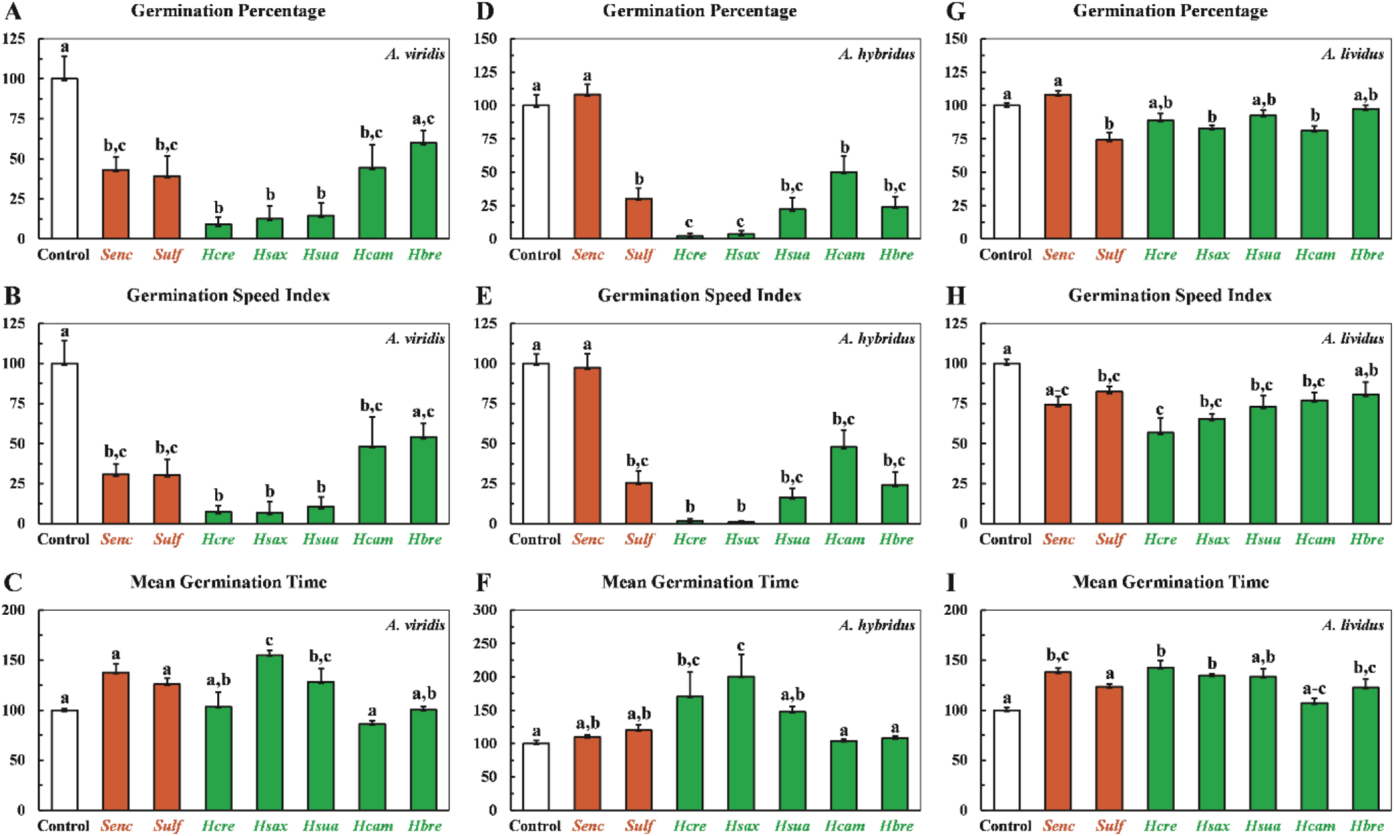
Mean values of germination process parameters of *A*. *viridis* (Charts A–C), *A*. *hybridus* (Charts D–F), and *A*. *lividus* (Charts G–I) after treatment with *Hyptis* extracts. Values are expressed as mean ± SEM (*n* = 7). Columns with different letters are significantly different (*p* < 0.05) from each other.

In A. viridis, the extracts *H*. *crenata* (***Hcre***), *H*. *suaveolens* (***Hsua***), and *H*. *saxatilis* (***Hsax***) significantly reduced the germination percentage (**GP**) by 91%, 88%, and 86%, respectively, compared to the control. These reductions exceeded those observed for the commercial herbicides ***Senc*** (57%) and ***Sulf*** (61%) (Figure 1 – Chart **A**). Similarly, the same extracts decreased the germination speed index (**GSI**) by 93%, 94%, and 90%, surpassing the reductions achieved by ***Senc*** (69%) and ***Sulf*** (70%) (Figure 1 – Chart **B**). Moreover, the mean germination time (**MGT**) increased by 55% (***Hsua***) and 28% (***Hsax***), indicating a delay in germination progression (Figure 1 – Chart **C**). Considering **MGT** is inversely related to **GSI**, these increases confirm a deceleration in the germination process following treatment with the extracts.

The extracts ***Hcre***, ***Hsua***, and ***Hsax*** exhibited strong phytotoxic effects on A. hybridus seed germination. The **GP** was reduced by 98, 96, and 78%, respectively (Figure 1D), while the **GSI** decreased by 98, 99, and 84%, respectively (Figure 1 – Chart **E**). Additionally, **MGT** increased by 70, 100, and 47% for ***Hcre***, ***Hsua***, and ***Hsax***, respectively (Figure 1 – Chart **F**), indicating delayed germination and confirming the inhibitory impact of the extracts on seedling development.

In the *A*. *lividus* assay, treatment with *Hyptis* extracts inhibited seed germination. Specifically, ***Hsax*** and ***Hcam*** reduced **GP** by 18 and 19%, respectively (Figure 1 – Chart **G**). The ***Hcre*** extract had the most pronounced effect on **GSI**, reducing it by 44% (Figure 1 – Chart **H**). Additionally, **MGT** increased by 42% for ***Hsua*** and 34% for ***Hsax***, indicating a delay in the germination progression (Figure 1 – Chart **I**).

Figure 2 illustrates the effects of Hyptis extracts on seedling growth, with all treatments exhibiting phytotoxic activity. In A. viridis and A. hybridus, the strongest effects were observed with ***Hcre*** and ***Hsax***, which reduced hypocotyl length (**HL**) and root length (**RL**). In A. viridis assay, ***Hcre*** and ***Hsax*** reduced **HL** by 67 and 72%, respectively (Figure 2 – Chart **A**), comparable to the commercial herbicides ***Senc*** (58%) and ***Sulf*** (51%). Similarly, **RL** was reduced by 59% with ***Hcre*** and 74% with ***Hsax***, relative to the negative control (Figure 2 – Chart **B**).

**Figure 2:**
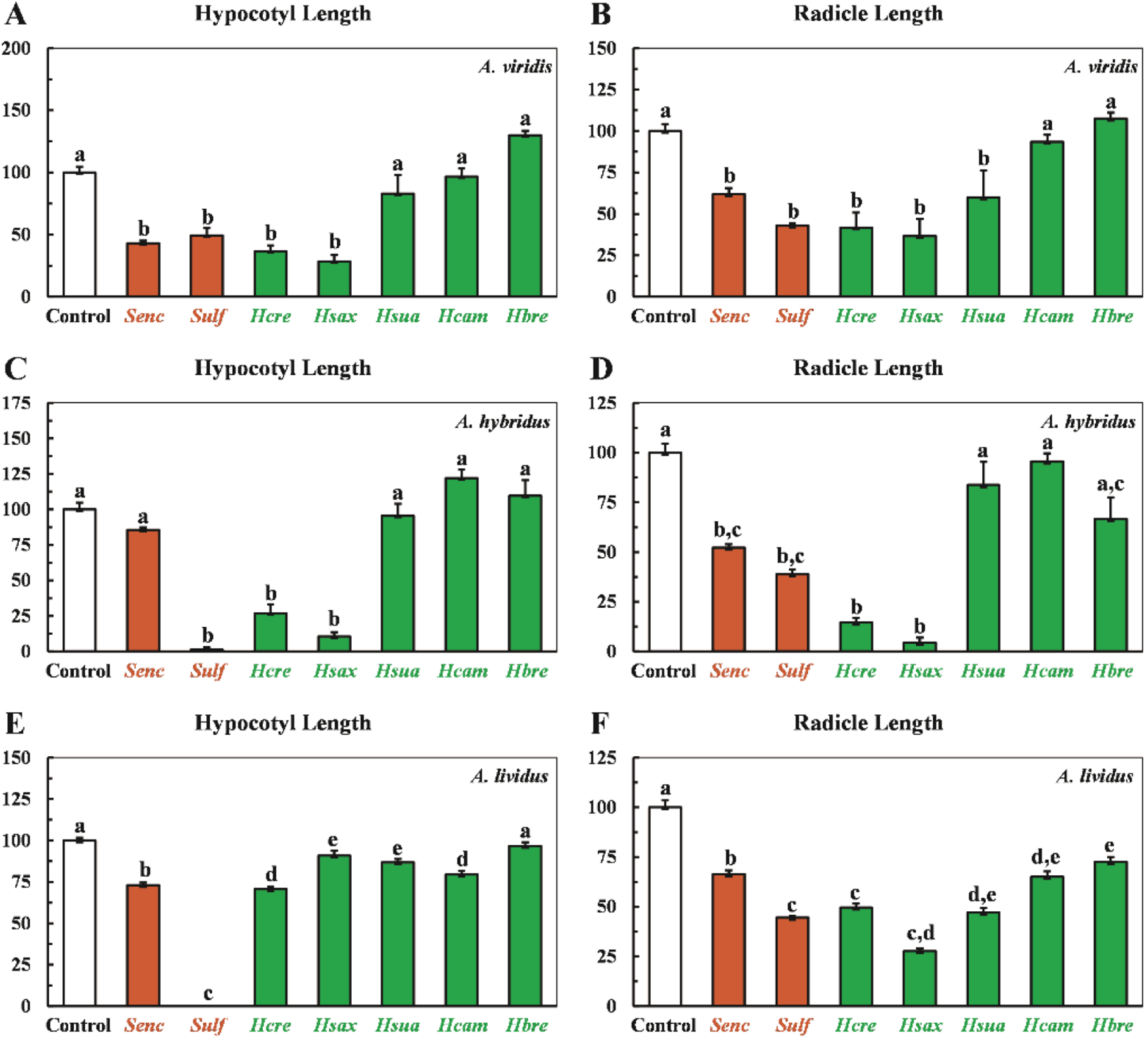
Mean values of hypocotyl and radicle lengths of *A*. *viridis* (Charts **A**, **B**), *A*. *hybridus* (Charts **C**, **D**), and *A*. *lividus* (Charts **E**, **F**) after treatment with *Hyptis* extracts. Values are expressed as mean ± SEM (*n* = 30). Columns with different letters are significantly different (*p* < 0.05) from each other.

In the *A*. *hybridus* assay, ***Hcre*** and ***Hsax*** reduced **HL** by 74 and 90%, respectively (Figure 2 – Chart **C**), comparable to ***Sulf*** (99%). Additionally, **RL** was reduced by 86% with ***Hcre*** and 96% with ***Hsax***, exceeding the reductions observed with the commercial herbicides (Figure 2 – Chart **D**). For *A*. *lividus*, all extracts except ***Hbre*** inhibited **HL**, with reductions ranging from 10 to 30% relative to the negative control (Figure 2 – Chart **E**). **RL** reductions ranged from 28% to 73%, with ***Hcre*** and ***Hsax*** showing the strongest inhibitory effects, comparable to ***Senc*** (44%) and ***Sulf*** (66%) (Figure 2 – Chart **F**). These results demonstrate that *Hyptis* extracts, particularly ***Hcre*** and ***Hsax***, exhibit strong and consistent phytotoxic effects on the growth of all evaluated weed species.

Compared to previous studies, the *Hyptis* extracts ***Hcre***, ***Hsua***, and ***Hsax*** exhibited stronger phytotoxic activity, achieving greater inhibition of germination at equal or lower concentrations than those reported for other plant-based extracts.^26–28^ ***Hcre*** and ***Hsax*** demonstrated superior suppression of weed germination after 7 days of exposure compared to the results of Quasem et al., who evaluated eight vegetable extracts against *Amaranthus retroflexus* over 14 day.^29^ Similarly, Khasabuli et al. reported that *Bidens pilosa* exerted significant allelopathic effects on *Amaranthus dubius*, with higher concentrations inhibiting both germination and seedling growth, particularly affecting root and shoot development.^30^ Together, these findings reinforce the potential of *Hyptis* extracts as promising sources of bioherbicidal compounds for managing *Amaranthus* species.

Moreover, the phytotoxic effects of *Hyptis* extracts have been documented against other problematic weed species, including *Eleusine coracana*,^31^ *E*. *crus-galli*,^32^ and *L*. *multiflorum*.^22^ These results further highlight the potential of *Hyptis* as a natural herbicide for sustainable weed management, where allelopathic interactions disrupt key metabolic pathways in target species, ultimately inhibiting their growth and development.^33^

The reduction in plant growth observed in this study demonstrates the selective phytotoxic activity of the Hyptis extracts at 200 ppm. This allelopathic potential against Amaranthus species is likely attributed to bioactive secondary metabolites present in Hyptis. Plant extracts often contain compounds capable of inhibiting germination and growth in other species, potentially by interference with indole-3-acetic acid biosynthesis via the tryptophan pathway, disruption of protein synthesis, or impairing ion absorption.^34^

Several *Hyptis* species have previously been recognized for their phytotoxic potential and proposed as candidates for bioherbicide development.^35^ For example, the secondary metabolite 6-methoxy-benzoxazolin-2­(3*H*)-one, isolated from *H*. *suaveolens*, strongly inhibits germination in eight weed species by blocking the induction of α-amylase, an enzyme essential for starch hydrolysis during seed germination and early seedling growth.^21^

The bioherbicidal potential of *Hyptis* species is closely associated with their structurally diverse secondary metabolites, particularly terpenes and flavonoids. Several terpenoid have been reported, including triterpene lactones from *Hyptis albida*,^36^ labdane diterpenoids from *Hyptis fasciculata*, isopimarane diterpenes from *Hyptis salzmanii*,^37^ and tricyclic diterpenes from *Hyptis dilatate*.^38^ In addition, flavonoids such as isoquercitrin from *H*. *fasciculata*,^39^ apigenin-*C*,*C*-diglucoside, and kaempferol-3-*O*-rutinoside from *Hyptis colombiana* ^19^ have been identified. Despite the chemical diversity of the genus, terpenoids remain the most prominent class of bioactive compounds, exhibiting a broad spectrum of biological activities.^40,41^

In summary, the increasing demand for safe and sustainable alternatives has driven the development of bioherbicides derived from plant extracts, which provide effective weed control without long-term persistence or broad toxicity.^42^ Among these, allelochemical-based bioherbicides are particularly promising, as they combine low toxicity, multiple modes of action, and rapid degradation, thereby minimizing risks to nontarget organisms and environmental accumulation.^8^ Our findings, demonstrating the herbicidal potential of Hyptis species, further support the feasibility of exploring allelochemical-inspired molecules as leads for the development of novel bioherbicides.

### Multivariate Statistical Analyses and Biomarkers Annotation

The principal component analysis (PCA) score plot (Figure 3 – Chart A) shows clear clustering of the treatments and the control, indicating high precision and stability of the analytical method. The first three principal components explain 74.3% of the total variance among the promising, nonpromising, and control treatments. The plot reveals three distinct clusters, with strong intragroup similarity observed for both promising and nonpromising treatments.

**Figure 3:**
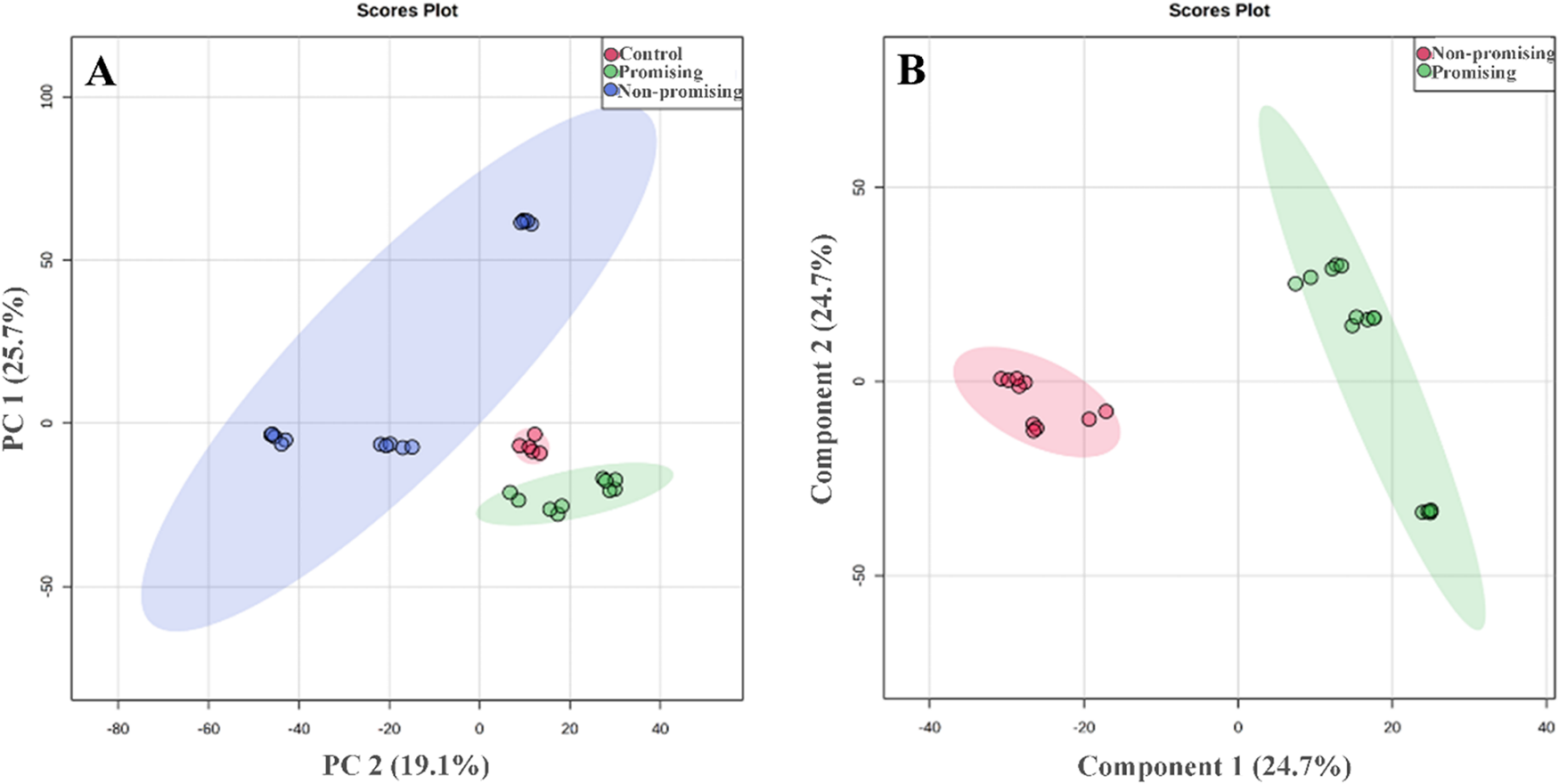
PCA score plot of the treatments (Chart - A) PLS-DA score plot supervised by the bioherbicide profile (Chart - B). Both analyses were performed using log transformation, normalization by sum, mean-centering scaling, and four components. The 95% Hotelling’s T^2^ ellipse is shown.

The partial least-squares - discriminant analysis (PLS-DA) score plot (Figure 3– Chart B) accounted for 70.2% of the total variance across the first three components. Model validation using 10-fold cross-validation yielded *R* ^2^ (goodness of fit) and *Q* ^2^ (predictive ability) values of 0.984 and 0.952, respectively. These values exceed commonly accepted thresholds for biological models (*R* ^2^ ≥ 0.5 and *Q* ^2^ ≥ 0.4), confirming the robustness and predictive reliability of the model.^43–45^

By examining the variable importance in projection (VIP) (Figure 4) and volcano plots (Figure 5), the key features responsible for sample discrimination and clustering, as well as those associated with promising bioherbicidal profiles, were identified. The main discriminative features - including *m*/*z*, retention time, putative annotations, and ion contributions - are summarized in Table S1 (Supporting Information). Feature IDs 1477, 839, 1269, 577, 560, 1046, 597, and 1385 were annotated as cannabigerivarin, phenylmethyl 2,3,4-tri-*O*-methyl-6-*O*-(triphenylmethyl)-β-d-glucopyranoside, 12,16-epoxy-11,14-dihydroxy-17(15→16)-abeo-abieta-8,11,13-trien-7-one, 7β-hydroxy-11,14-dioxoabieta-8,12-diene, rosmanol, aspewentin A, katsumain B, and (−)-ainsliadimer B, which were predominantly detected in extracts ***Hcre*** and ***Hsax***.

**Figure 4:**
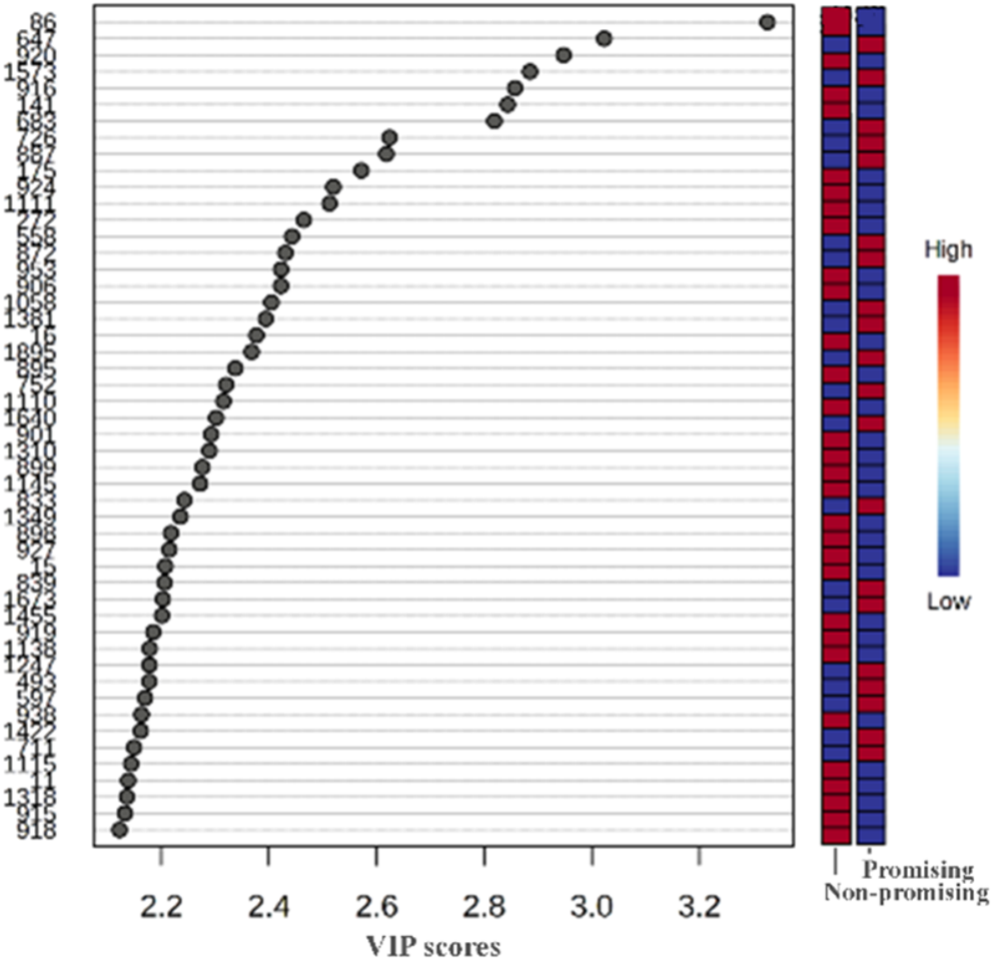
VIP scores indicating the top 50 important metabolites contributing to the separation of metabolic profiles in nonpromising/promising samples, with relative abundance of metabolites is indicated by a colored scale from blue to red representing the low and high, respectively.

**Figure 5:**
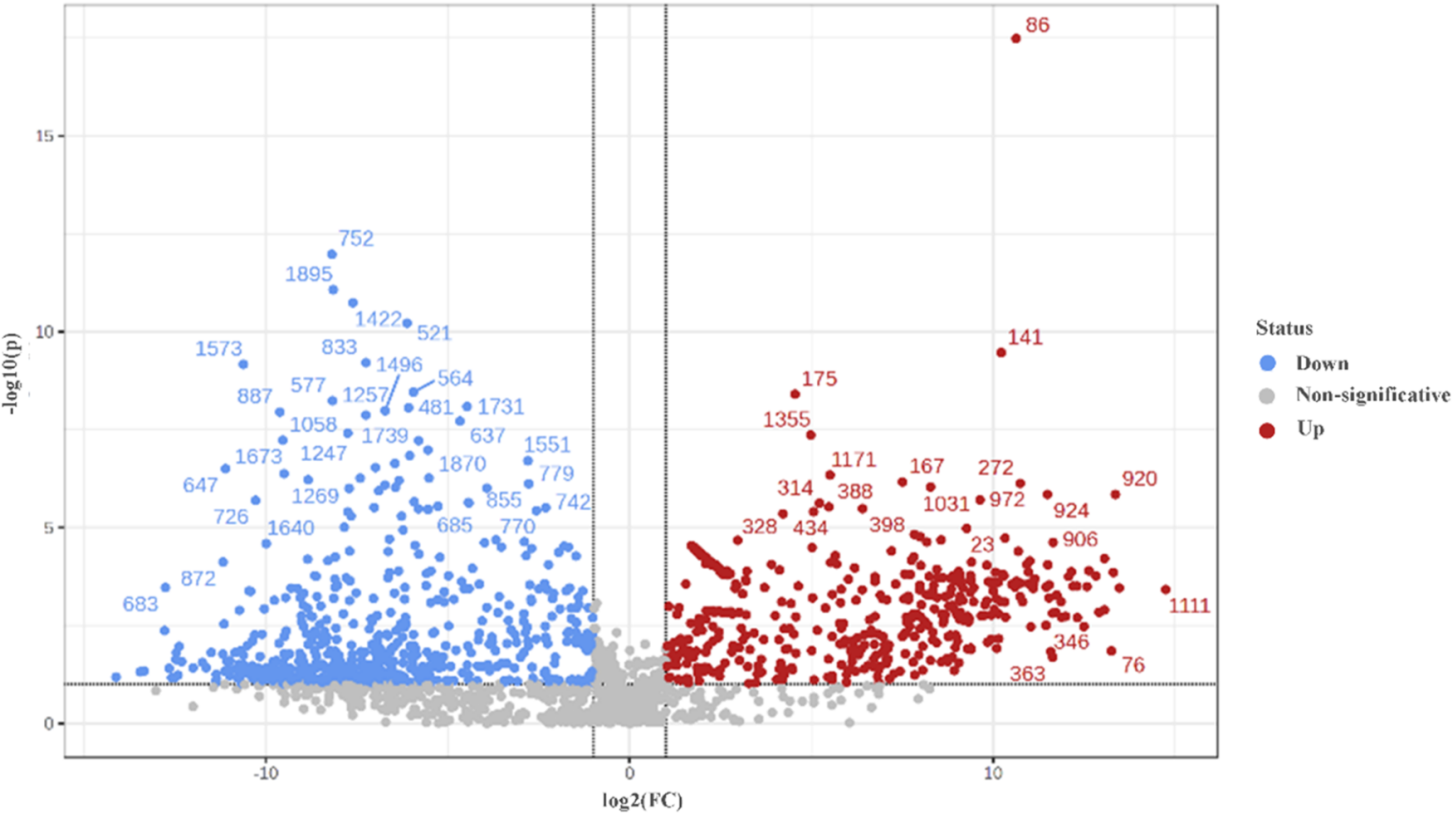
Volcano Plot indicating differential analysis between the VIPs from nonpromising/promising samples with *p*-value of 0.1.

Feature IDs 1381, 1220, 1496, and 1266 were putatively annotated as 19-acetoxy-2α,7α-dihydroxylabda-14,15-dinorlabd-8(17)-en-13-one, kaurenoic acid, pectinolide F, and 2-ethyl-3-methylbutyl 6-*O*-(6-deoxy-α-*L*-mannopyranosyl)-β-d-glucopyranoside, and were primarily associated with extracts ***Hsax*** and ***Hsua***. Additionally, feature IDs 1685, 711, 493, and 548 were annotated as kaempferol-3-*O*-(2-*O*-β-d-apiofuranosyl)-α-*L*-rhamnopyranoside, carasiphenol D, apigenin-7-(2-*O*-apiosylglucoside), and hopeachinol A, were more abundant in extracts ***Hcre*** and ***Hsua***, as visualized in the heatmap (Figure 6).

**Figure 6:**
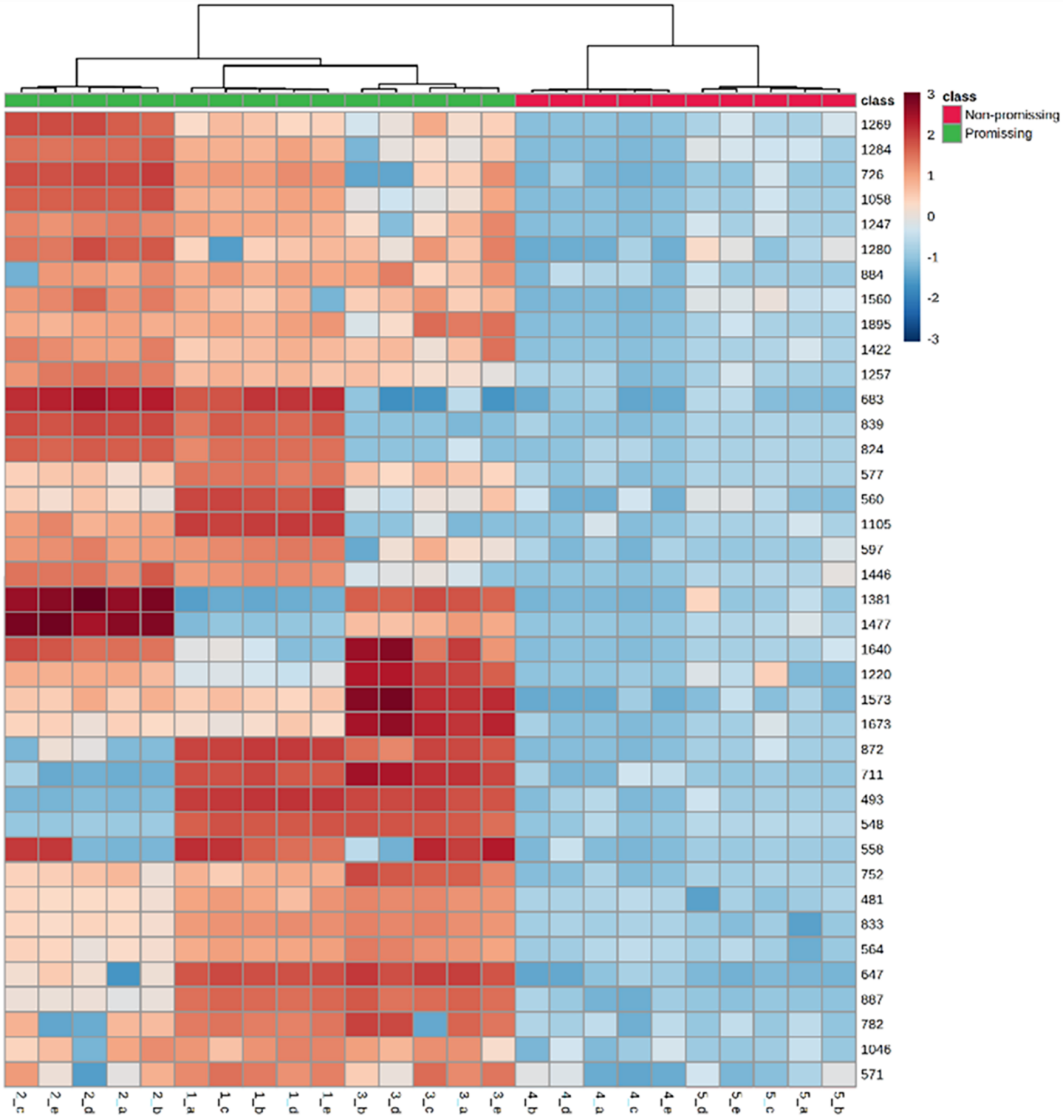
Heatmap of the IDs (metabolites) and the 40 highest VIP score values of the PLS-DA model, correlated to promising samples, for data visualization. Red color indicates high expression and blue indicates low expression in the plant extracts.

### Chemical Profile of Bioactive *Hyptis* Extracts

Given the significant phytotoxic effects observed for the ***Hcre***, ***Hsua***, and ***Hsax*** extracts, the chemical profiles of the corresponding Hyptis species were investigated. Metabolite annotation revealed a broad diversity of secondary metabolites, including 29 terpenoids, 7 flavonoid glycosides, 2 chalcones, 3 α-pyrones, 3 lactones, and 12 compounds classified as carbohydrates or polyketide derivatives, totaling 56 annotated compounds. The high abundance of terpenoid derivatives and flavonoid glycosides may underlie the observed phytotoxicity.

Among the putatively annotated compounds associated with the observed phytotoxic effects, 20 have been previously reported in the Hyptis genus, including various terpenes, flavonoids, pyrones, and lactones (Table S1, Supporting Information). Notably, for **Hcre** and **Hsax**, none of the annotated metabolites have been described previously, indicating that these compounds are reported for the first time in these species. In **Hsua**, only two identified compounds - the triterpene betulin (**22**) and the abietane diterpenoid 7α-ethoxyroyleanone (**6**) - have been reported previously. Of the compounds identified in the chemical profile, 52% were classified as terpenoids, as illustrated in Figure 7. Terpenes are widely recognized for their diverse biological activities, including inhibition of seed germination,^46^ insecticidal^47^ and herbicidal effects,^48^ suppression of root and shoot growth, and induction of disease symptoms in plants.^49^

**Figure 7:**
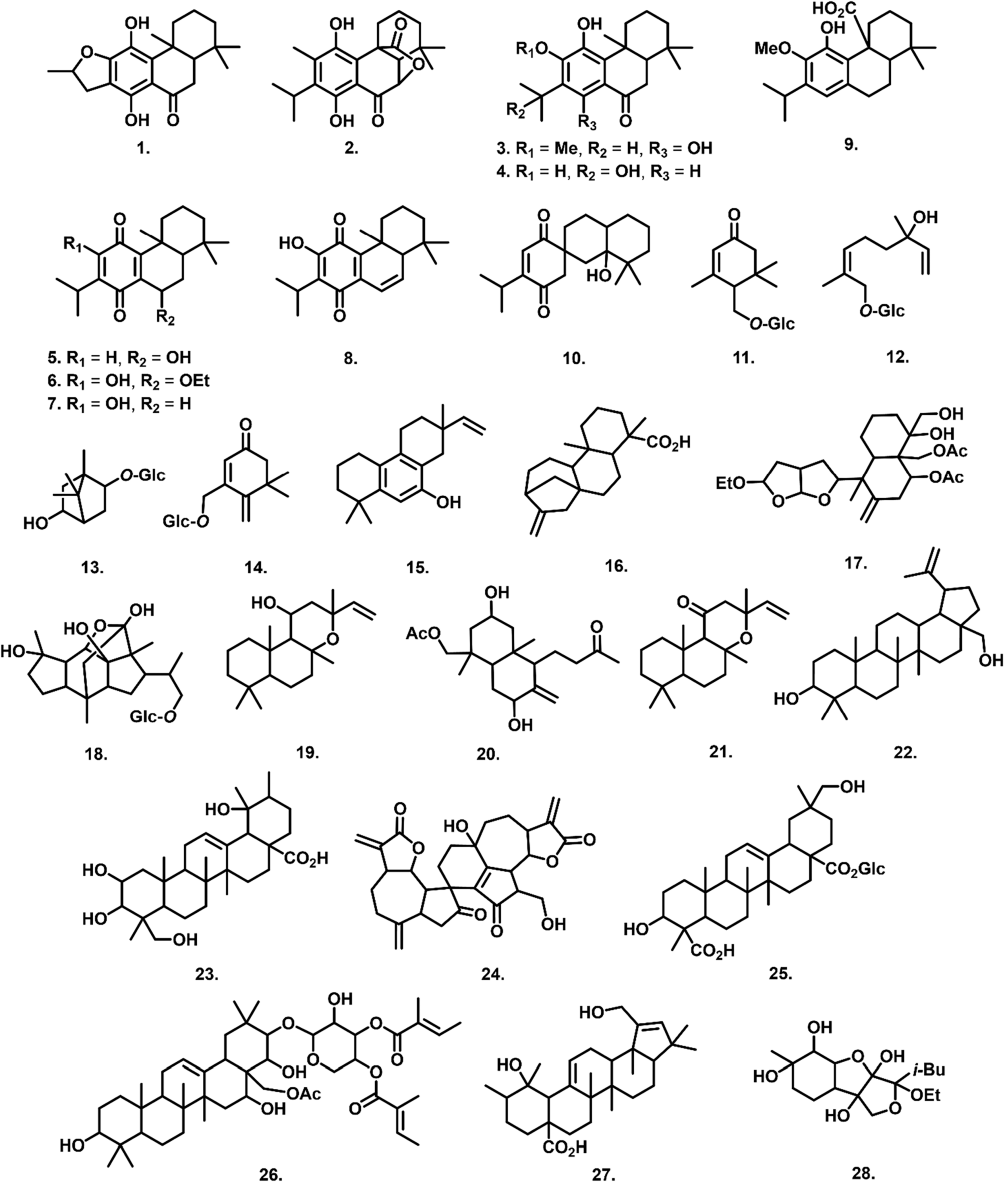
Chemical structure of terpenes annotated as biomarkers in the ***Hcre***, ***Hsua***, and ***Hsax*** extracts.

Diterpenes were the most representative subclass in chemical profile, comprising 16 compounds, of which 10 exhibited the characteristic tricyclic scaffold of abietane diterpenoids. Abietane diterpenoids can be classified into 14 structural categories.^50^ In this study, three distinct classes were identified: (i) aromatic abietanes (compounds **1–5**), (ii) *para*-quinone abietanes (compounds **6–9**), and (iii) a rearranged abietane diterpenoid (compound **10**), there by expanding the structural diversity of abietanes detected in *Hyptis* species.

Abietane-type diterpenes, such as those previously isolated from the roots of *Hyptis martiusii*, *Hyptis platanifolia*, and *Hyptis carvalho*, have been widely investigated for their biological activities, including antioxidant,^51^ antibacterial,^52^ cytotoxic,^53^ antileishmanial,^54^ topoisomerase I inhibitory,^55^ and seed germination inhibitory effects.^56^

Metabolites **1–3** were detected in the mass spectra as protonated adducts [M + H]^+^ with *m*/*z* values of 331.1906 (C_20_H_27_O_4_), 347.1851 (C_20_H_27_O_5_) and 347.2218 (C_21_H_31_O_4_), respectively. Abietane **1** was annotated as 12,16-epoxy-11,14-dihydroxy-17(15→16)-abeo-abieta-8,11,13-trien-7-one, a diterpenoid previously reported in Hyptis crassifoli*a*.^51^ Similarly, rosmanol (**2**) and 11,14-dihydroxy-12-methoxyabieta-8,11,13-triene-7-one (**3**) have been described in Hyptis *dilatata* ^38^ and Hyptis *verticillate*,^57^ respectively. All three metabolites exhibited neutral losses of water and carbon monoxide [M+H–H_2_O–CO]^+^ in their MS/MS fragmentation patterns, yielding ions at *m*/*z* 285.1824 (**1**),^51^ *m*/*z* 301.1766 (**2**),^58^ and *m*/*z* 301.2132 (**3**),^59^ consistent with their structural assignments.

The aromatic abietane diterpenoid 11,12,15-trihydroxy-8,11,13-abietatrien-7-one (**4**) has been previously reported in *H*. *crassifolia*.^60^ This compound was detected as a sodium adduct ion [M + Na]^+^, with an observed *m*/*z* of 355.1871, corresponding to the molecular formula C_20_H_28_O_4_Na. Compound **5**, with an observed *m*/*z* of 347.2218 and a calculated molecular formula of C_21_H_30_O_4_, was the only abietane in this study to yield a dehydrated ion [M-H_2_O+H]^+^. The base peak was detected at *m*/*z* 329.2062 (C_21_H_29_O_3_), indicating a prominent loss of water. This compound was annotated as 12-methoxycarnosic acid, previously described in *H*. *martiusii*.^61^

Metabolites **6–9** were annotated as *para*-quinone abietanes diterpenoids, characterized by carbonyl substitutions at the C-11 and C-14 positions. Some compounds, such as **8**, also exhibited 6,7-unsaturation.^62^ Compound **8** generated protonated adducts [M + H]^+^, whereas compounds **6**,^63^ **7**,^64^ and **9** ^53^ were detected as sodium adducts [M + Na]^+^. These abietanes displayed limited fragmentation in their MS/MS spectra, a feature commonly associated with the stability of the quinonoid structure. Compound **6** was detected at *m*/*z* 383.2195 [M + Na]^+^, corresponding to the molecular formula C_22_H_32_O_4_Na. Structurally similar to royleanone (**7**), it features an ethoxy substituent at C-7 and was annotated as 7α-ethoxyroyleanone, previously reported in *Hyptis camaroides*.^65^

Metabolites **7** and **8** were classified as royleanone abietanes, differing in their degree of unsaturation and hydroxylation patterns at positions C-6, C-7, and C-12. Compound **7**, annotated as royleanone, was observed at *m*/*z* 339.1938 ([M + Na]^+^, C_20_H_28_O_3_Na), whereas compound **8**, identified as 6,7-dehydroroyleanone, was detected at *m*/*z* 315.1952 ([M + H]^+^, C_20_H_27_O_3_). Both compounds have been previously isolated from *H*. *verticillate*.^57^

Compound **9**, an isomer of compound **7**, differs in the hydroxyl group position, located at C-12 (ring C) in compound **7** and at C-7 (ring B) in compound **9**. It was detected at *m*/*z* 339.1929 as a sodium adduct and annotated as 7β-hydroxy-11,14-dioxoabieta-8,12-diene (C_20_H_28_O_3_Na). This metabolite has also been reported in H. martiusii.^53^

Compound **10** was classified as a rearranged abietane diterpenoid, distinguished by a spiro-fused tricyclic skeleton with a spiro linkage at C-9.^66^ It was detected as a sodium adduct ion [M + Na]^+^, with an observed *m*/*z* of 341.2076 and a calculated molecular formula of C_20_H_30_O_3_Na. This compound was annotated as martiusane, a rare diterpene previously reported in *H*. *martiusii*.^66^

Aromatic abietanes represent the most dominant class of abietane diterpenoids, consistent with their reported prevalence. These compounds are characterized by an aromatic C-ring and are frequently involved in plant chemical defense mechanisms.^67^ In this study, all annotated aromatic abietanes were hydroxylated at the C-11 position, a structural feature that may influence their biological activity.

Except for compound **4**, the aromatic abietanes produced abundant and informative MS/MS fragment ions. Several compounds exhibited conserved fragmentation patterns, commonly involving neutral losses of water (18 Da), carbon monoxide (28 Da), and an additional 46 Da fragment, potentially corresponding to formic acid (HCOOH) or another carboxyl-derived moiety. Detailed mass spectral data for all compounds acquired in positive ionization mode are provided in Table S1.

## Conclusion

In this study, the bioherbicidal potential of plant extracts from *Hyptis* species was evaluated against weed species of the *Amaranthus* genus. The extracts exhibited significant phytotoxic effects, evidenced by reductions in seed germination and seedling growth, indicating the presence of potent allelochemicals. Multivariate statistical analyses corroborated the biological data, effectively distinguishing the most active extracts from the less effective ones. Metabolomic profiling revealed 56 secondary metabolites, primarily terpenoid, along with flavonoid glycosides, chalcones, and lactones.

These results highlight the potential of *Hyptis*-based bioherbicides as sustainable and environmentally friendly alternatives to synthetic herbicides. Future research should focus on isolating and characterizing the bioactive constituents, elucidating their mechanisms of action, and evaluating their efficacy and safety in practical applications. Specifically, field trials are needed to assess herbicidal performance under agricultural conditions, and crop toxicity tests should be conducted to ensure selectivity and safety for nontarget plants.

## Experimental Section

### Plant Material

*H*. *crenata*, *H*. *suaveolens*, and *H*. *campestris* were collected in Santo Antônio do Leverger, Mato Grosso, Brazil, at coordinates S15°55′53.3″, W055°56′18.7″; S15°50′30.1″, W56°04′28.0″; and S15°48′17.9″, W56°04′47.6″, respectively. *H*. *saxatilis* and *H*. *brevipes* were collected in Cuiabá, Mato Grosso, Brazil, at S15°31′55.6″, W56°02′06.7″. All specimens were collected in May 2021. Plant specimens were identified and deposited in the Central Herbarium of the Federal University of Mato Grosso under the following registration numbers: *H*. *crenata* (UFMT 44471), *H*. *suaveolens* (UFMT 45386), *H*. *saxatilis* (UFMT 44469), *H*. *campestris* (UFMT 44472), and *H*. *brevipes* (UFMT 44470).

Fresh aerial parts of *H*. *crenata* (240 g), *H*. *suaveolens* (340 g), *H*. *saxatilis* (460 g), *H*. *campestris* (230 g), and *H*. *brevipes* (360 g) were extracted with 150 mL of ethanol using homogenizer-assisted extraction. Extractions were performed with an Ultra-Turrax dispersing device (IKA-T25, Staufen, Germany) at 12,000 rpm for 5 min. The mixtures were subsequently filtered under reduced pressure, and the extraction procedure was repeated twice for each plant. The resulting extracts were combined, ethanol was removed under reduced pressure, and the residues were lyophilized and stored at −80 °C. The final extract yields were as follows: *H*. *crenata* (***Hcre***, 10.080 g, 4.20%), *H*. *suaveolens* (***Hsua***, 3.330 g, 0.97%), *H*. *saxatilis* (***Hsax***, 3.083 g, 0.67%), *H*. *campestris* (***Hcam***, 2.251 g, 0.98%), and *H*. *brevipes* (***Hbre***, 1.089 g, 0.30%).

### Phytotoxicity Assay

Commercially available *Amaranthus* seeds were obtained from Agro Cosmos Company (Engenheiro Coelho, Brazil). Seeds of *A*. *viridis*, *A*. *hybridus*, and *A*. *lividus* were surface-sterilized with sodium hypochlorite and thoroughly rinsed with distilled water. The sterilized seeds were placed in autoclaved Petri dishes (9 cm in diameter) lined with germination paper. Each dish was moistened with 4 mL of distilled water, and 20 seeds were evenly distributed per dish.

The control group was treated with dimethyl sulfoxide (DMSO) alone, while the experimental groups received 200 mg/L (200 ppm) solutions of extracts ***Hcre***, ***Hsua***, ***Hsax***, ***Hcam***, and ***Hbre***. Each treatment was performed with seven replicates, arranged in a completely randomized design. The plates were incubated at room temperature (25–30 °C), and germination was monitored daily. Seeds were considering germinated when the emerging seedling reached 2 mm in length. After 7 days, germination percentage as well as hypocotyl and radicle lengths were recorded, and percent inhibition was calculated.^68^

The **GP** was calculated as the ratio of germinated seeds to the total number of seeds sown. The **GSI** was determined according to the equation: **GI** = ∑(*Gi* × *I*), where *Gi* represents the number of germinated seeds on day *i*, and *I* is the corresponding day after incubation. The **MGT** was calculated as **MGT** = ∑(*Gi* × *I*)/∑(*Gi*). The **RL** and **HL** of the germinated seedlings were measured using a digital caliper.^69^

Data were analyzed by analysis of variance (ANOVA) using SPSS software (version 25.0, IBM Corp., Armonk, NY). When significant differences were detected (*p* ≤ 0.01 or *p* ≤ 0.05), means were compared using Tukey’s Honestly Significant Difference test.^70^

### UPLC-MS/MS Data Acquisition

The crude extracts of *Hyptis* were analyzed using a Shimadzu liquid chromatograph (Tokyo, Japan) coupled to a micrOTOF-Q II mass spectrometer (Bruker Daltonics, Boston, MA). The system was equipped with an electrospray ionization (ESI) source and a quadrupole time-of-flight (QTOF) analyzer.

Chromatographic separation was performed on a reverse-phase C18 column (150 × 4.6 mm, 5 μm; Phenomenex, Torrance, CA) maintained at 35 °C. The mobile phase consisted of solvent A (0.1% formic acid in ultrapure water; Sigma-Aldrich, St. Louis, MO) and solvent B (acetonitrile). The gradient program was as follows: 0–20 min, linear increase from 5% to 100% B; followed by a 3 min hold at 100% B. The injection volume was 1 μL of extract solution in methanol (1 mg/mL).^71^

Mass spectra were acquired in positive ion mode over the *m*/*z* range of 50 to 1100 Da using data-dependent acquisition (MS1 and MS2). Instrument parameters were set as follows: nebulizer gas pressure of 4.5 bar, drying gas flow rate of 9.0 L/min, capillary voltage of 3500 V, and ion source temperature of 220 °C. Automated fragmentation was applied to the four most intense ions in each spectrum, with collision-induced dissociation energies ranging from 20 to 105 eV depending on the precursor ion. After MS/MS acquisition, precursor ions were released for subsequent scans. Data were processed using Compass DataAnalysis 4.3 software (Bruker Daltonics, Boston, MA).

### Molecular Networking and Metabolite Annotation using GNPS

The acquired MS data were converted from. d to. mzXML format using MZmine 2.53 software.^72^ Data processing parameters were set according to general chromatographic characteristics: mass detection (noise level: 1.0 × 10^3^), chromatogram builder (threshold: 1.0 × 10^4^; *m*/*z* tolerance: 0,002), deconvolution (threshold: 5%; retention time (RT) range: 0.02 min; minimum relative height: 15%), deisotoping (*m*/*z* tolerance: 5 ppm; RT tolerance: 0.2 min), alignment (join aligner: *m*/*z* tolerance: 5 ppm; RT tolerance: 0.2 min; weight for *m*/*z*: 15, RT: 0.1 min), gap filling (intensity, *m*/*z* and RT tolerance: 10%, 5 ppm, and 0.2 min, respectively), and annotation (*m*/*z* and RT tolerance: 5 ppm and 30 min).

Molecular networks (MNs) were generated using the GNPS platform (https://gnps.ucsd.edu) through the classical molecular networking workflow.^73^ UPLC-MS/MS data were processed in positive ionization mode with the following parameters: cosine similarity score threshold of 0.5, minimum of six matched fragment ions, parent mass tolerance of 0.02 Da, and fragment ion tolerance of ± 0.05 Da. The maximum molecular family size was set to 100 nodes. The resulting network spectra were matched against GNPS spectral libraries. Visualization and network analysis were carried out in Cytoscape version 3.9.1.^74^

The MNs generated in positive ionization mode are accessible through the following links:

**Hcre** *:* https://gnps.ucsd.edu/ProteoSAFe/status.jsp?task=a604952de8d146c2bd32d906183e480e

***Hsua***:https://gnps.ucsd.edu/ProteoSAFe/status.jsp?task=e70e81f584284ad6ab13df17da121d04

***Hsax***:https://gnps.ucsd.edu/ProteoSAFe/status.jsp?task=11bd38d123bd4a1d9807f9a3167b07c3

***Hcam***:https://gnps.ucsd.edu/ProteoSAFe/status.jsp?task=7c015ea396e44a819c1a320df1d4f0a9

***Hbre***: https://gnps.ucsd.edu/ProteoSAFe/status.jsp?task=538bd191465a4adfb91c339bd9802ab3

Metabolite annotation was performed using the *in-house* database *HyptisDB* (Table S1, Supporting Information), which compiles natural products previously reported from *Hyptis* species, in combination with the GNPS molecular networking. Dereplication was further supported by cross-referencing the Dictionary of Natural Products, PubChem, and SciFinder. Following current metabolomics identification standards, most compounds were assigned a confidence level of 3. For certain known metabolites with higher-intensity MS signals, annotation confidence was upgraded to levels 2 or 3 based on detailed analysis of their fragmentation patterns.^75–77^

### Multivariate Data Analyses

UPLC–MS/MS data sets of *Hyptis* extracts in positive ionization mode were analyzed using multivariate statistical methods, including PCA, PLS-DA, and Volcano Plot (VP), with MetaboAnalyst 5.0 software (Montreal, Canada).

Analyses were conducted using a data matrix of 30 samples × 1895 variables, comprising promising, nonpromising, and blank samples based on bioherbicide assay results. For PCA and PLS-DA, the data were sum-normalized, log-transformed, and mean-centered. Blank samples were excluded from PLS-DA, which was validated using 10-fold cross-validation. VP were generated using unpaired analysis, a fold-change threshold of 2.0, comparison direction set as nonpromising versus promising, and a *p*-value threshold of 0.1.^78^

Bioherbicide activity correlations and VIP scores were obtained from PLS-DA using four components, while correlations with promising samples were further analyzed using VP. Metabolites with VIP values greater than 1.5 and negative log2­(FC) values were selected for annotation. Heatmaps were generated for data visualization using VIP scores, Euclidean distance metrics, and the Ward clustering method.

## Supplementary Material



## Notes

Table S1: Metabolites identified and annotated in the extracts of *Hyptis crenata*, *Hyptis suaveolens*, and *Hyptis saxatilis*; Figure SI 01–56: Mass spectrum of metabolites **1–56**. Table S2: *Hyptis* species database (PDF)
