# Supplementary material for: Phytotoxic Metabolites from Hyptis Species: Chemical Profiling and Bioherbicide Activity Against Amaranthus
Source: ACS Omega. 2025 Oct 18;10(42):50359–70. doi: 10.1021/acsomega.5c07637 (PMC12572983; doi:10.1021/acsomega.5c07637)
Supplement: Supplementary file 1 [file ao5c07637_si_001.pdf]

Supporting Information

Phytotoxic Metabolites from *Hyptis* Species: Chemical Profiling and Bioherbicide Activity Against *Amaranthus*

Mariana A. Silva,° *Letícia P. F. da Silva,*° *Karen J. Nicácio,*° *Barbara S. Bellete, Lucas C. C. Vieira*° *and Olívia M. Sampaio*° *

° Chemistry Department, Federal University of Mato Grosso, 78060-900, Cuiabá – MT, Brazil

Chemistry Department, Federal University of Lavras, 37200-900, Lavras-MG, Brazil

Table S1. Metabolites identified and annotated in the extracts of *Hyptis crenata*, *Hyptis suaveolens*, and *Hyptis saxatilis*.

Entry	VIP	Structure name	*m/z*	Molecular formula	Espécie	Class	Error (ppm)	Adduct	Ref.
**1**	2.1060	12,16-Epoxy-11,14-dihydroxy-17(15→16)-abeo-abieta-8,11,13-trien-7-one	331.1906	$C_{20}H_{26}O_4$	*H. suaveolens* *H. crenata*	Abietane Diterpene	0.1	$[M+H]^+$	LIMA, 2015.
**2**	1.8966	Rosmanol	347.1851	$C_{20}H_{26}O_5$	*H. crenata*	Abietane Diterpene	-0.6	$[M+H]^+$	CHEN, 2020.
**3**	1.7394	11,14-Dihydroxy-12-methoxyabieta-8,11,13-triene-7-one	347.2218	$C_{21}H_{30}O_4$	*H.suaveolens*	*Abietane* Diterpene	0.9	$[M+H]^+$	BHAT, 1975.
**4**	2.0545	11,12,15-Trihydroxy-8,11,13-abietatrien-7-one	355.1871	$C_{20}H_{28}O_4$	*H. suaveolens* *H. saxatilis* *H. crenata*	Abietane Diterpene	1.1	$[M+Na]^+$	LIMA, 2015.
**5**	1.7395	12-Methoxycarnosic acid	347.2218	$C_{21}H_{30}O_4$	*H. suaveolens* *H. crenata*	Abietane Diterpene	-1.4	$[M+H]^+$	PUKALSKA, 2005.
**6**	1.7860	7α-ethoxyroyleanone	383.2195	$C_{22}H_{32}O_4$	*H. saxatilis* *H. crenata* *H. suaveolens*	Abietane Diterpene	-2.4	$[M+Na]^+$	MICHAVILA, 1985.
**7**	1.6682	Royleanone	339.1938	$C_{20}H_{28}O_3$	*H. suaveolens* *H. saxatilis* *H. crenata*	Abietane Diterpene	-2.7	$[M+Na]^+$	DUAN, 2022.
**8**	1.5153	6,7-Dehydroroyleanone	315.1952	$C_{20}H_{26}O_3$	*H. suaveolens* *H. saxatilis* *H. crenata*	Abietane Diterpene	2.5	$[M+H]^+$	HENSCH, 1975.
**9**	2.0650	7-ß-hydroxy-11,14-Dioxoabieta-8,12-diene	339.1929	$C_{20}H_{28}O_3$	*H. crenata* *H. suaveolens* *H. saxatilis*	Abietane Diterpene	0.4	$[M+Na]^+$	ARAÚJO, 2006.
**10**	1.6309	Martiusane	341.2076	$C_{20}H_{30}O_3$	*H. saxatilis* *H. crenata* *H. suaveolens*	Abietane Diterpene	1.9	$[M+Na]^+$	DA CRUZ ARAÚJO, 2004.
**11**	2.8851	NCGC00385950-01_C16H26O7_(2,6,6-Trimethyl-4-oxo-2-cyclohexen-1-yl) methyl beta-D-glucopyranoside	353.1575	$C_{16}H_{26}O_7$	*H. saxatilis* *H. crenata* *H. suaveolens*	Monoterpene	-1.5	$[M+Na]^+$	N/A

**12**	2.0171	NCGC00380237-01_C16H28O7_6-Hydroxy-2,6-dimethyl-2,7-octadien-1-yl D-glucopyranoside	355.1719	$C_{16}H_{28}O_7$	*H. suaveolens*<br>*H. crenata*<br>*H. saxatilis*	Monoterpene	2.5	$[M+Na]^+$	N/A
**13**	1.9657	Angelicoidenol 2-O-beta-D-glucopyranoside	355.1723	$C_{16}H_{28}O_7$	*H. suaveolens*<br>*H. saxatilis*<br>*H. crenata*	Monoterpene	0.8	$[M+Na]^+$	KITAJIMA, 1998.
**14**	1.7741	Jasminoside A	353.1574	$C_{16}H_{26}O_7$	*H. saxatilis*<br>*H. crenata*<br>*H. suaveolens*	Monoterpene	-0.6	$[M+Na]^+$	ZHU, 2015.
**15**	2.0188	Jasminoside C	311.1493	$C_{16}H_{24}O_7$	*H. suaveolens*	Monoterpene	-2.4	$[M-H_2O+H]^+$	ZHAO, 2012.
**16**	1.9470	Aspewentin A	271.2062	$C_{19}H_{27}O$	*H. suaveolens*<br>*H. crenata*	Monoterpene	-3.1	$[M+H]^+$	MIAO, 2014.
**17**	1.9187	Kaurenoic acid	325.2144	$C_{20}H_{30}O_2$	*H. saxatilis*<br>*H. suaveolens*	Diterpene	-0.4	$[M+Na]^+$	ALI, 2023.
**18**	1.6449	Clerodiol	521.2718	$C_{26}H_{42}O_9$	*H. saxatilis*<br>*H. suaveolens*<br>*H. crenata*	Diterpene	-0.4	$[M+Na]^+$	N/A
**19**	1.6833	Cinncassiol D1 glucoside	537.2663	$C_{26}H_{42}O_{10}$	*H. crenata*<br>*H. saxatilis*<br>*H. suaveolens*	Diterpene	1.30	$[M+Na]^+$	NOHARA, 1981.
**20**	1.8300	11β-Hydroxymanoyl oxide	329.2442	$C_{20}H_{34}O_2$	*H. crenata*	Diterpene	-3.6	$[M+Na]^+$	MIRZAEI, 2020.
**21**	2.3941	19-acetoxy-2a,7a-dihydroxylabda-14,15-dinorlabd-8(17)-en-13-one	375.2136	$C_{20}H_{32}O_5$	*H. suaveolens*<br>*H. saxatilis*<br>*H. crenata*	Diterpene	1.7	$[M+Na]^+$	FRAGOSO-SERRANO, 1999.
**22**	1.7866	11-Oxomanoyl oxide	327.2291	$C_{20}H_{32}O_2$	*H. saxatilis*<br>*H. crenata*	Diterpene	0.2	$[M+Na]^+$	MAHMOUT, 2001.
**23**	2.4429	Betulin	465.3686	$C_{30}H_{50}O_2$	*H. crenata*<br>*H. saxatilis*	Triterpenoid	3.2	$[M+Na]^+$	ZHANG, 2019
**24**	1.9391	Hyptatic acid B	527.3335	$C_{30}H_{48}O_6$	*H. crenata*<br>*H. saxatilis*<br>*H. suaveolens*	Triterpenoid	2.3	$[M+Na]^+$	YAMAGISHI, 1988.
**25**	1.6960	(-)-Ainsliadimer B	503.2088	$C_{30}H_{32}O_8$	*H. suaveolens*<br>*H. crenata*	Triterpenoid	-4.8	$[M-H_2O+H]^+$	WANG, 2008.
**26**	1.5176	Ilexhainanoside E	687.3698	$C_{36}H_{56}O_{11}$	*H. crenata*<br>*H. saxatilis*<br>*H. suaveolens*	Triterpenoid	2.5	$[M+Na]^+$	N/A
**27**	1.8147	Majideagenin	829.5086	$C_{47}H_{72}O_{12}$	*H. suaveolens*	Triterpenoid	1.6	$[M+H]^+$	N/A

**28**	1.5174	Coleonolic acid	453.3344	$C_{30}H_{46}O_4$	*H. saxatilis*	Triterpenoid	4.3	$[M-H_2O+H]^+$	OBAROAKPO, 2020.
**29**	1.7035	Virgineol	355.1722	$C_{16}H_{28}O_7$	*H. crenata* *H. saxatilis* *H. suaveolens*	Sesquiterpenoid	1.6	$[M+Na]^+$	ASANO, 2013.
**30**	2.1777	Apigenin-7-(2-O-apiosylglucoside)	565.1558	$C_{26}H_{28}O_{14}$	*H. crenata* *H. saxatilis*	Flavonoid Glycoside	-0.6	$[M+H]^+$	SAIDI, 2023.
**31**	1.9758	Kaempferol3-O-neohesperidoside	595.1660	$C_{27}H_{30}O_{15}$	*H. crenata* *H. saxatilis* *H. suaveolens*	Flavonoid Glycoside	-0.3	$[M+H]^+$	DAUGUET, 1993.
**32**	1.8300	Quercetin	303.0500	$C_{15}H_{10}O_7$	*H. crenata* *H. saxatilis* *H. suaveolens*	Flavonoid Glycoside	-1.4	$[M+H]^+$	NAVARRETE, 2024.
**33**	1.5308	Kaempferol-3-O-(2-O-β-D-apiofuranosyl)-α-L-rhamnopyranoside phenoside A	565.1545	$C_{26}H_{28}O_{14}$	*H. crenata* *H. saxatilis*	Flavonoid Glycoside	1.3	$[M+H]^+$	N/A
**34**	1.7895	8-Formylisorhamentin 3-O-robinobioside	635.1591	$C_{29}H_{32}O_{17}$	*H. saxatilis*	Flavonoid Glycoside	2.2	$[M-H_2O+H]^+$	WANG, 2017.
**35**	1.9570	Apigenin 7-O-β-D-apiofuranosyl(1→2)-β-D-glucopyranoside	587.1377	$C_{26}H_{28}O_{14}$	*H. crenata* *H. saxatilis*	Flavonoid Glycoside	-0.9	$[M+Na]^+$	LIU, 2016.
**36**	1.9309	Kaempferol3-O-rutinoside	617.1478	$C_{27}H_{30}O_{15}$	*H. crenata* *H. saxatilis* *H. suaveolens*	Flavonoid Glycoside	0.0	$[M+Na]^+$	ASTITI, 2021
**37**	2.0180	Pectinolide A	311.1493	$C_{16}H_{22}O_6$	*H. suaveolens*	α-pyrone	-2.4	$[M+H]^+$	N/A
**38**	1.8404	Pectinolide F	441.1736	$C_{21}H_{30}O_{11}$	*H. suaveolens* *H. saxatilis*	α-pyrone	4.4	$[M-H_2O+H]^+$	BOALINO, 2003.
**39**	1.6418	Monticolide A	411.1671	$C_{20}H_{28}O_{10}$	*H. saxatilis* *H. crenata*	α-pyrone	-4.7	$[M-H_2O+H]^+$	DA SILVA, 2021.
**40**	1.7685	Hyptolide	369.1527	$C_{18}H_{24}O_8$	*H. saxatilis* *H. crenata* *H. suaveolens*	Lactone	1.3	$[M+H]^+$	SABITHA, 2013.
**41**	2.1697	Katsumain B	535.2507	$C_{35}H_{35}O_5$	*H. crenata* *H. saxatilis* *H. suaveolens*	Chalcone	-2.2	$[M+H]^+$	LI, 2010.
**42**	1.7895	Hydroxysafflor yellow B	635.1591	$C_{27}H_{32}O_{16}$	*H. saxatilis*	Chalcone	-1.6	$[M+Na]^+$	YUE, 2014.

**43**	1.7035	Maculalactone A	355.1722	$C_{25}H_{22}O_2$	*H. crenata*<br>*H. saxatilis*	Lactone	-7.7	$[M+H]^+$	BROWN, 2004.
**44**	1.8147	21-Hydroxyl-oligomycin A	829.5086	$C_{45}H_{74}O_{12}$	*H. suaveolens*<br>*H. crenata*	Lactone	-1.3	$[M+Na]^+$	N/A
**45**	2.3205	Amorphigenol O-glucoside	573.1952	$C_{29}H_{34}O_{13}$	*H. saxatilis*<br>*H. crenata*<br>*H. suaveolens*	Roteinoids	1.6	$[M-H_2O+H]^+$	N/A
**46**	2.2060	Phenylmethyl 2,3,4-tri-O-methyl-6-O-(triphenylmethyl)-β-D-glucopyranoside	537.2665	$C_{35}H_{38}O_6$	*H. saxatilis*<br>*H. crenata*<br>*H. suaveolens*	Carbohydrate	-5.7	$[M-H_2O+H]^+$	N/A
**47**	2.1494	Carasiphenol D	587.1374	$C_{35}H_{23}O_9$	*H. saxatilis*<br>*H. crenata*	Oligostilbenoids	-5.8	$[M+H]^+$	WANG, 2005.
**48**	1.9578	Hopeachinol A	587.1377	$C_{35}H_{22}O_9$	*H. saxatilis*<br>*H. crenata*	Polyphenol	-6.1	$[M+H]^+$	GE, 2010.
**49**	1.7899	Origanine A	635.1591	$C_{29}H_{30}O_{16}$	*H. saxatilis*	Polyphenol	0.4	$[M+H]^+$	LIU, 2012.
**50**	1.7416	Symplocuronic acid	565.1559	$C_{26}H_{30}O_{15}$	*H. saxatilis*<br>*H. crenata*	Phenolic glycoside	-9.6	$[M-H_2O+H]^+$	AHMAD, 2011.
**51**	1.6789	(2*R*)-2-Ethyl-3-methylbutyl 6-O-(6-deoxy-*α*-*L*-mannopyranosyl)-β-D-glucopyranoside	447.2202	$C_{19}H_{36}O_{10}$	*H. saxatilis*<br>*H. suaveolens*	Diglicoside	0.5	$[M+Na]^+$	N/A
**52**	1.5193	Cylindrocyclophane D	691.4182	$C_{40}H_{60}O_8$	*H. crenata*<br>*H. suaveolens*	Polyketide	2.3	$[M+Na]^+$	MOORE, 1992.
**53**	1.5104	Capsofulvesin A	765.4544	$C_{43}H_{66}O_{10}$	*H. crenata*	glycolipide	-3.6	$[M+Na]^+$	FANG, 2012.
**54**	1.7416	Acetylramosin C	565.1559	$C_{24}H_{30}O_{14}$	*H. saxatilis*<br>*H. crenata*	glycoside	-4.4	$[M+Na]^+$	JEWERS, 1975.
**55**	1.6036	(4R)-Dihydro-4-[[6-O-[(2E)-3-(4-hydroxy-3-methoxyphenyl)-1-oxo-2-propen-1-yl]-β-D-glucopyranosyl]oxy]-2(3H)-furanone	463.1242	$C_{20}H_{24}O_{11}$	*H. crenata*	phenolic glycoside	-3.4	$[M+Na]^+$	N/A
**56**	1.9971	Cannabigerivarin	271.2062	$C_{19}H_{28}O_2$	*H. crenata*<br>*H. suaveolens*	cannabinoids	2.5	$[M-H_2O+H]^+$	N/A

N/A = Not applicable

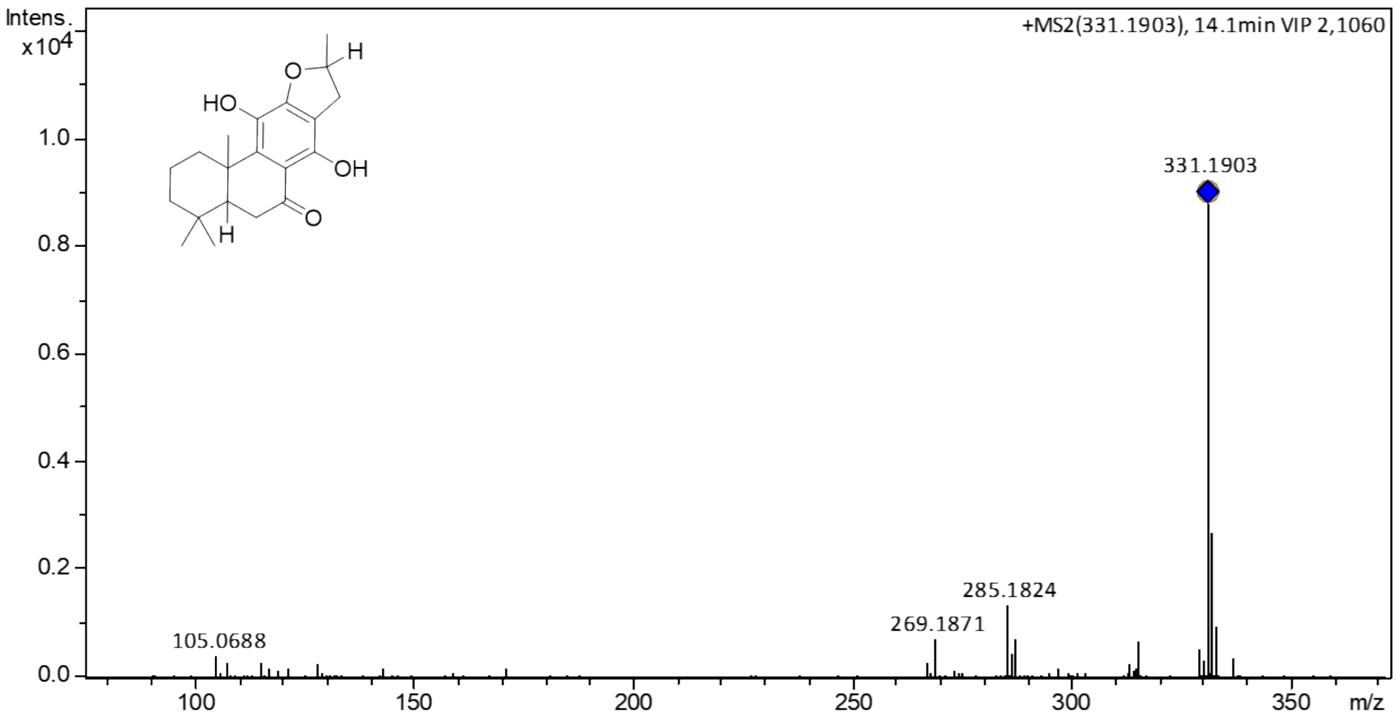

Figure SI 01. Mass spectrum of metabolite **1**.

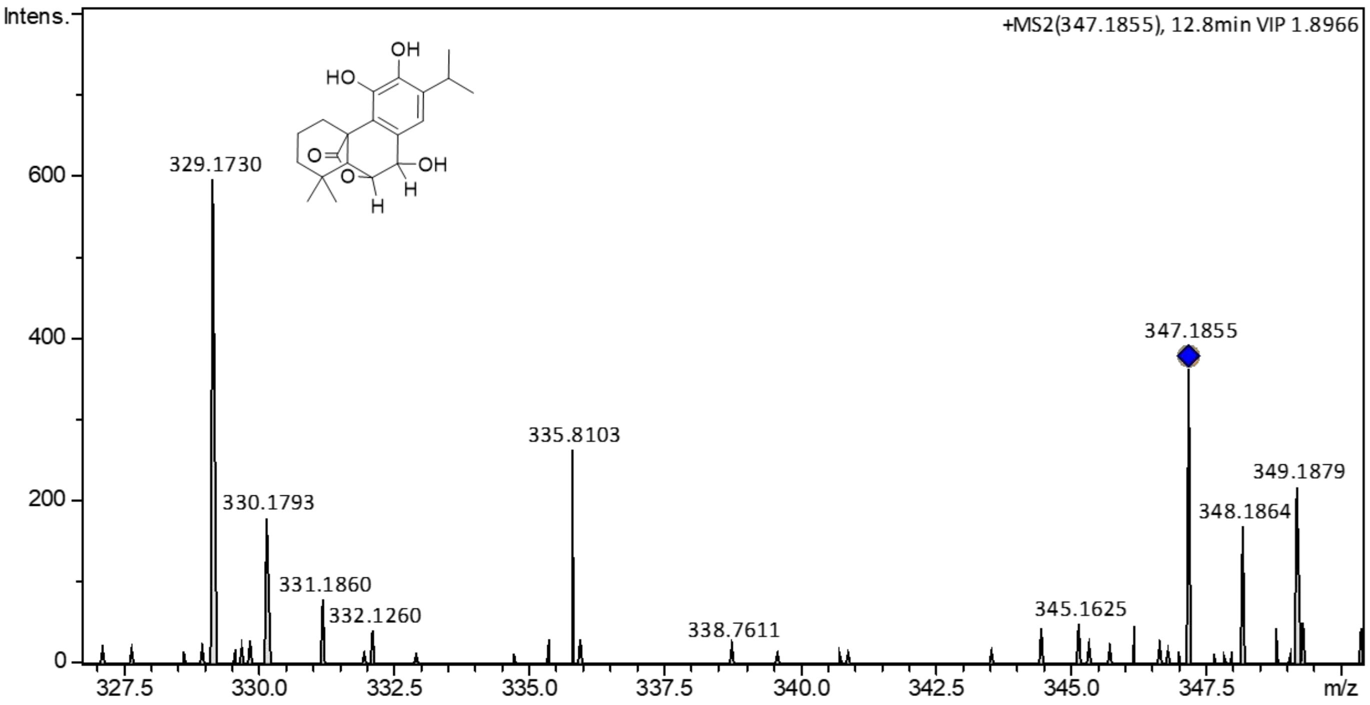

Figure SI 02. Mass spectrum of metabolite **2**.

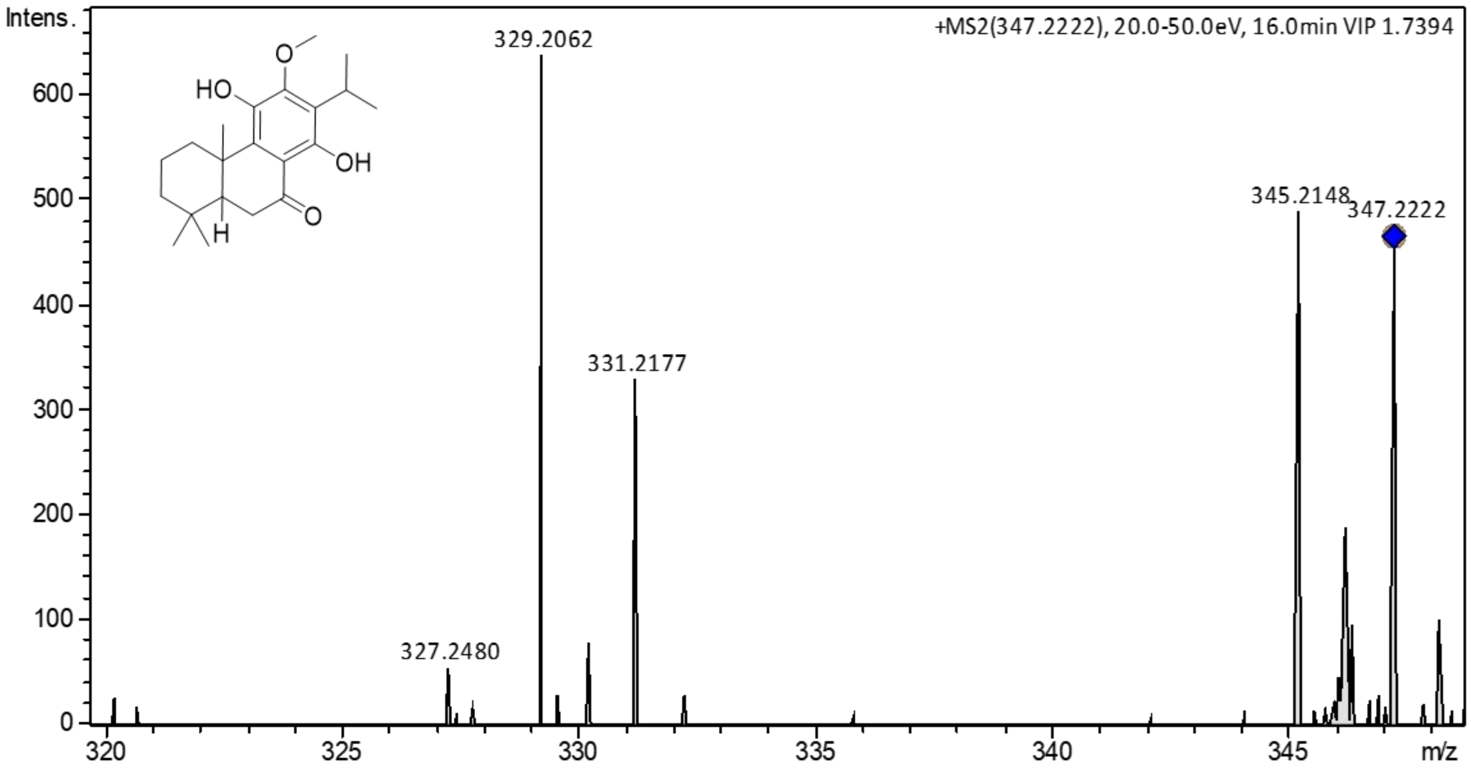

Figure SI 03. Mass spectrum of metabolite **3**.

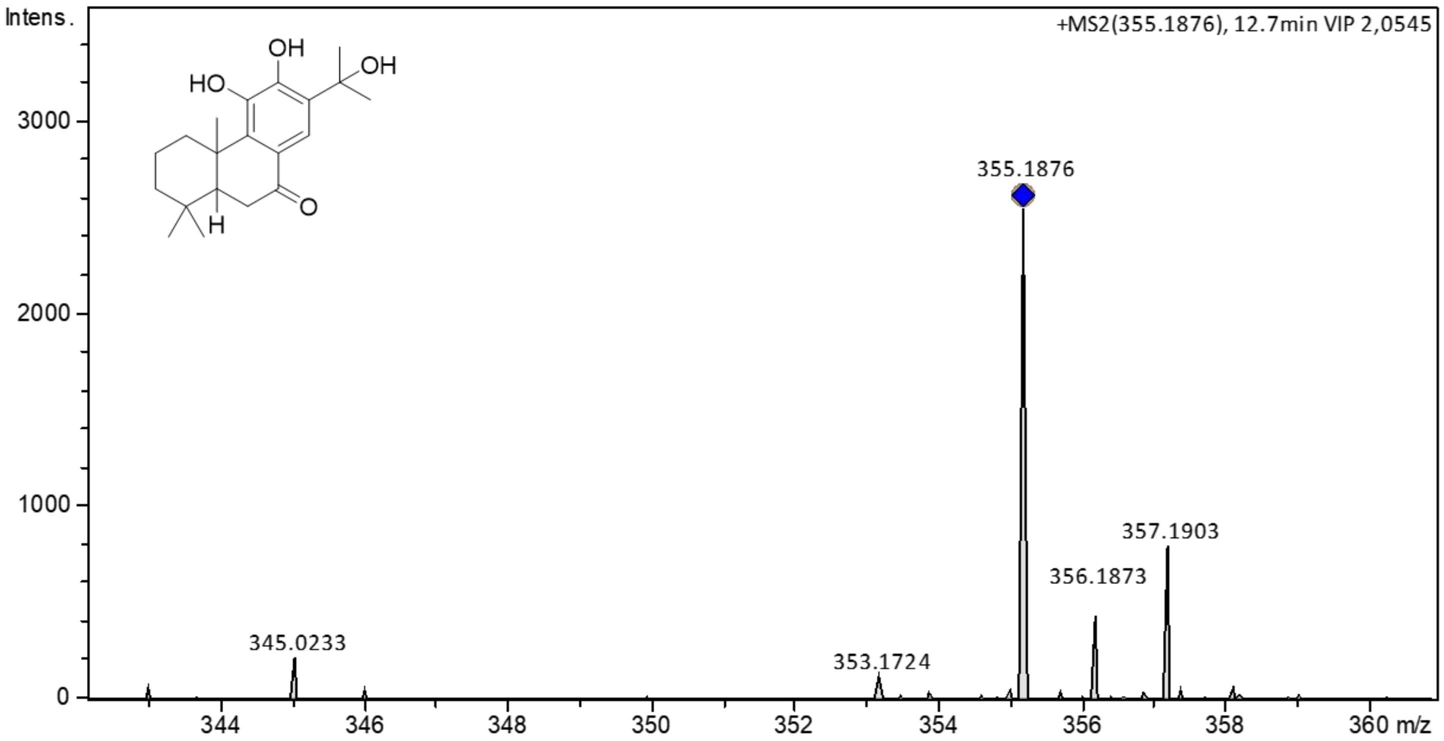

Figure SI 04. Mass spectrum of metabolite **4**.

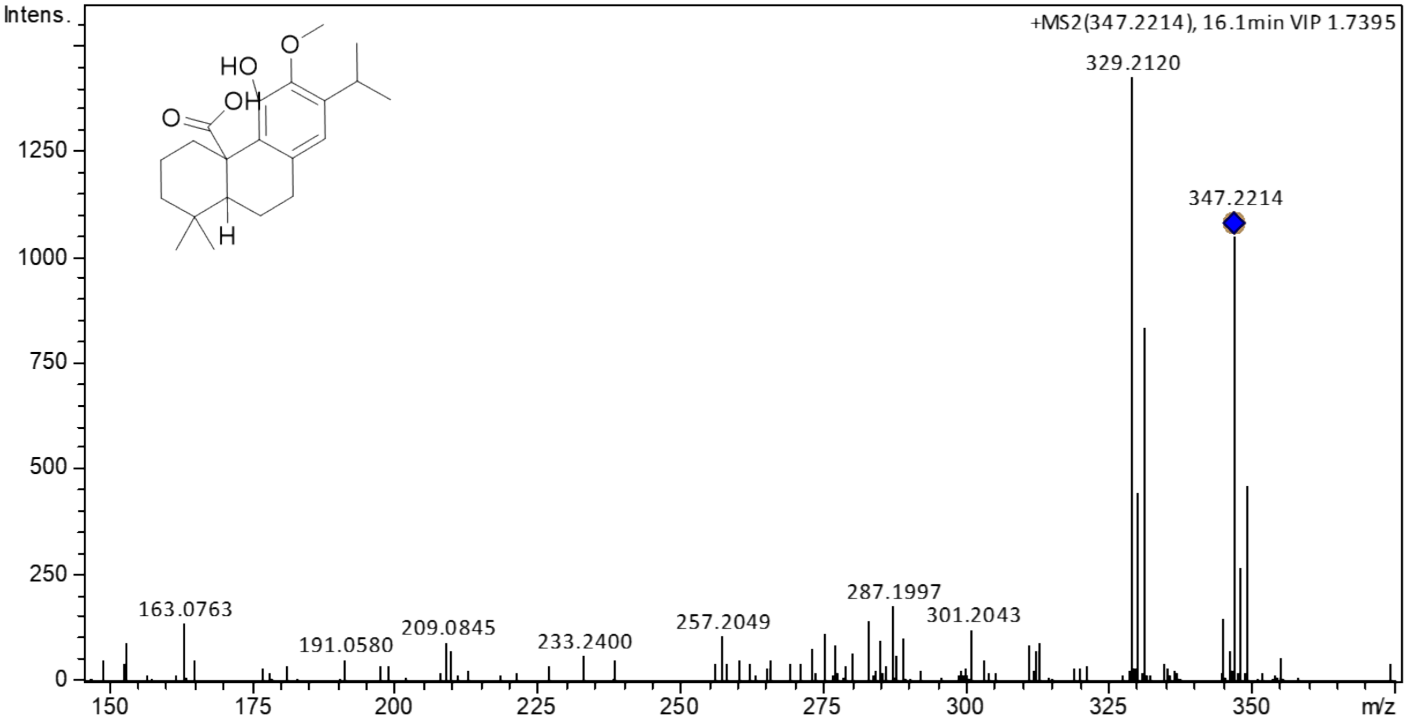

Figure SI 05. Mass spectrum of metabolite **5**.

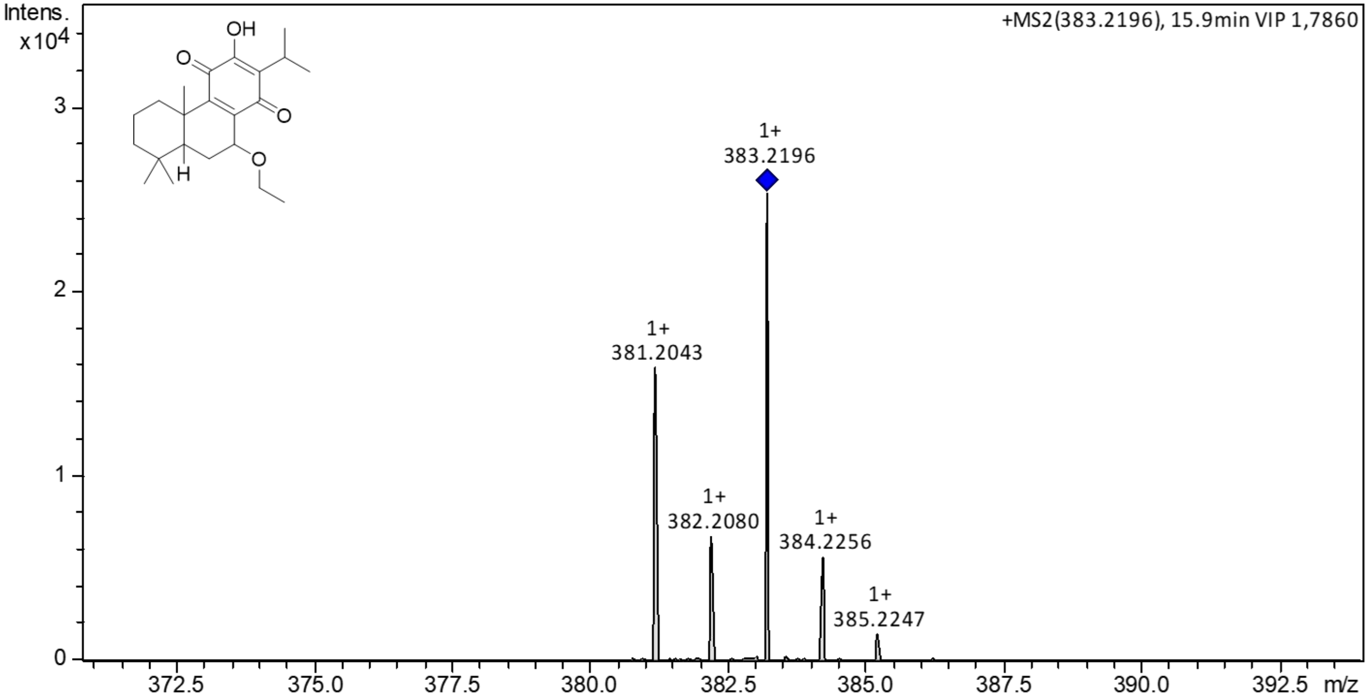

Figure SI 06. Mass spectrum of metabolite **6**.

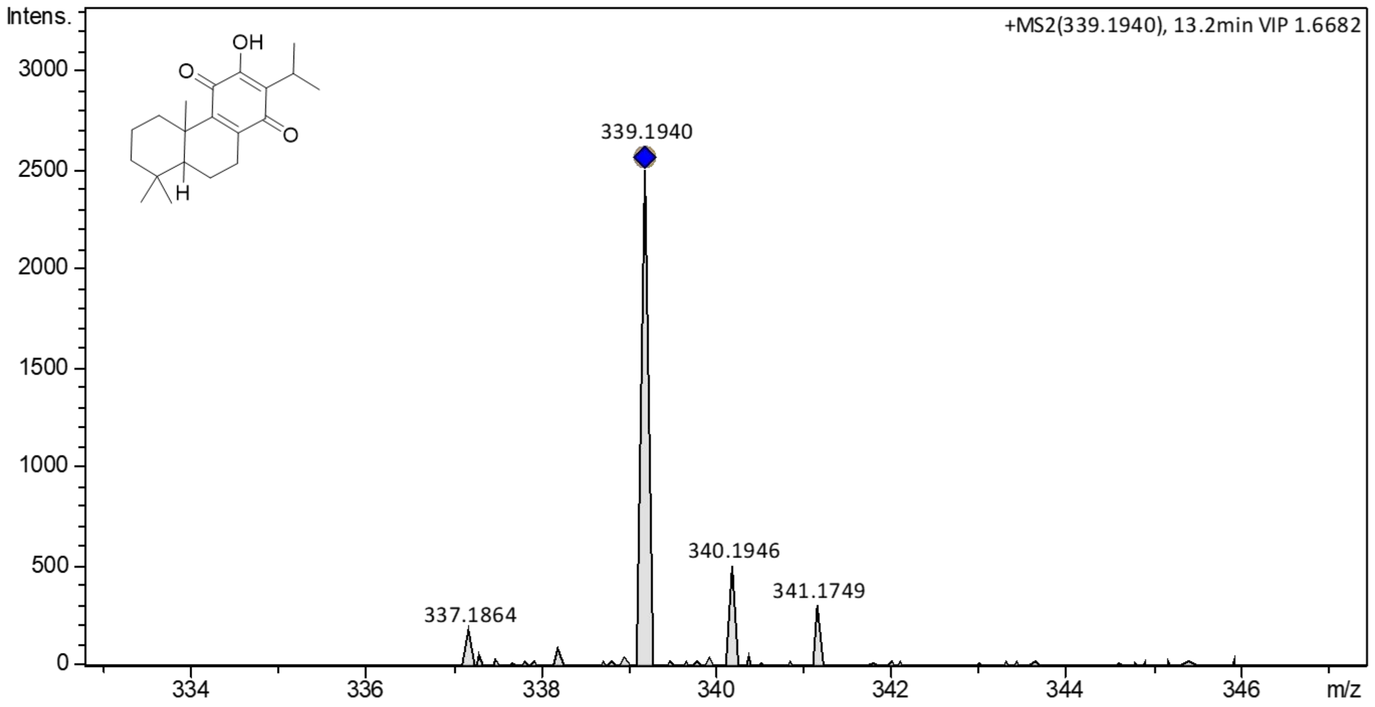

Figure SI 07. Mass spectrum of metabolite **7**.

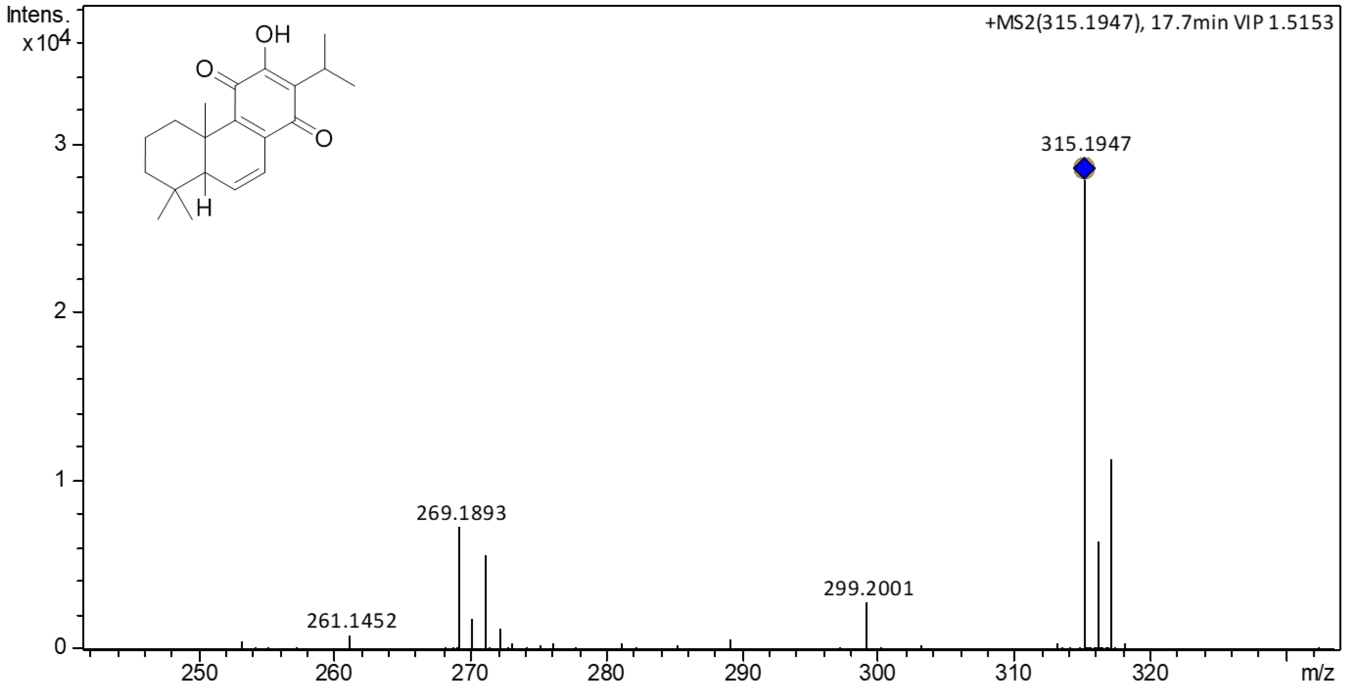

Figure SI 08. Mass spectrum of metabolite **8**.

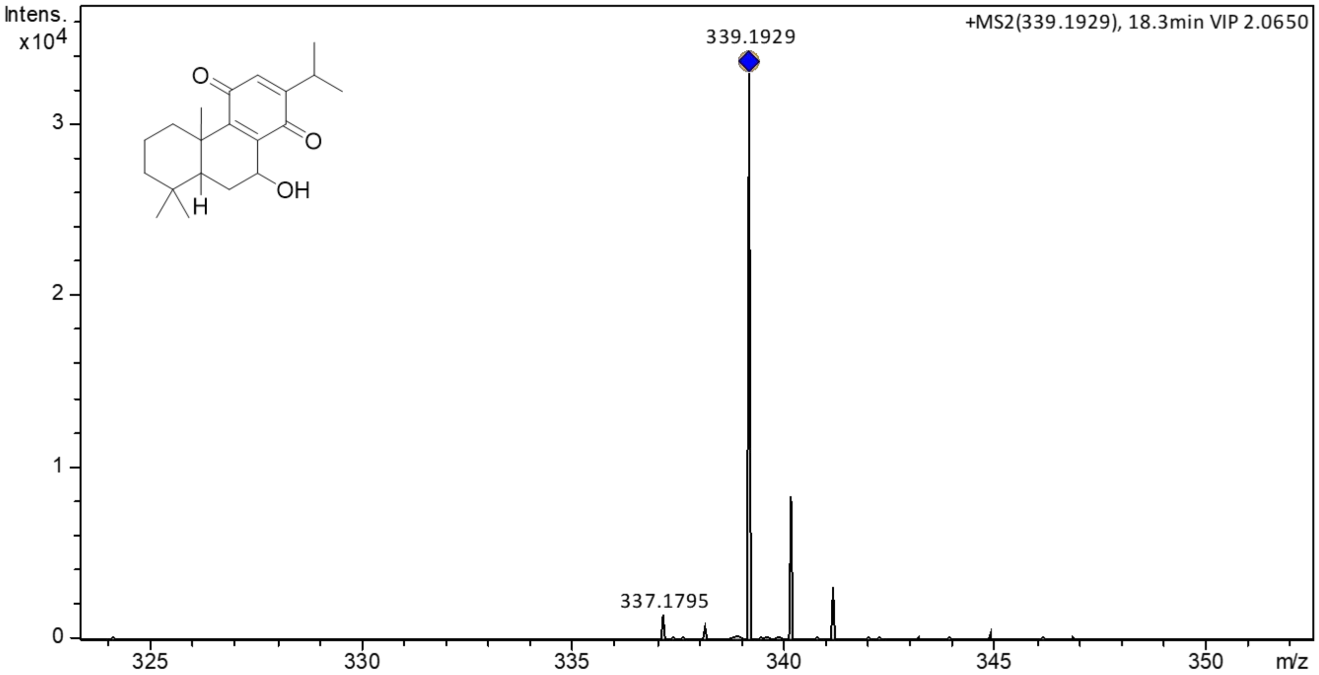

Figure SI 09. Mass spectrum of metabolite **9**.

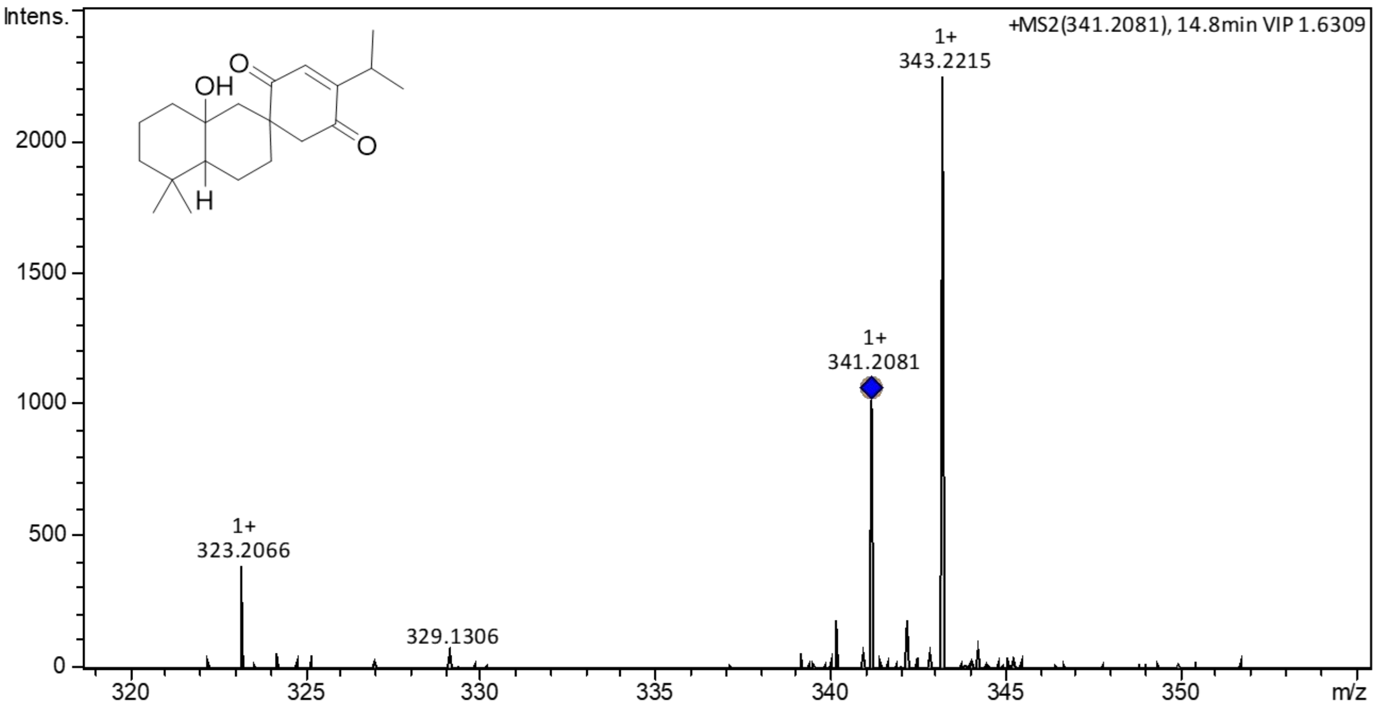

Figure SI 10. Mass spectrum of metabolite **10**.

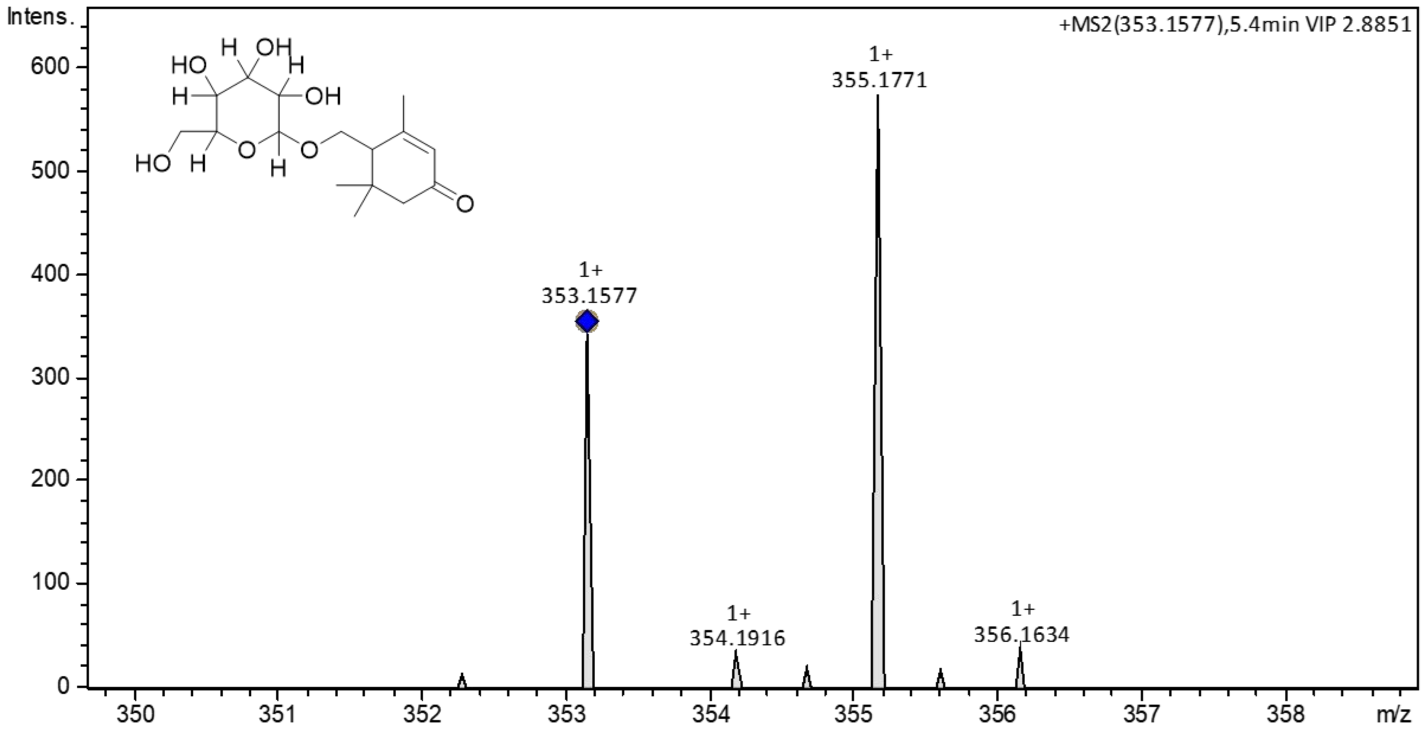

Figure SI 11. Mass spectrum of metabolite **11**.

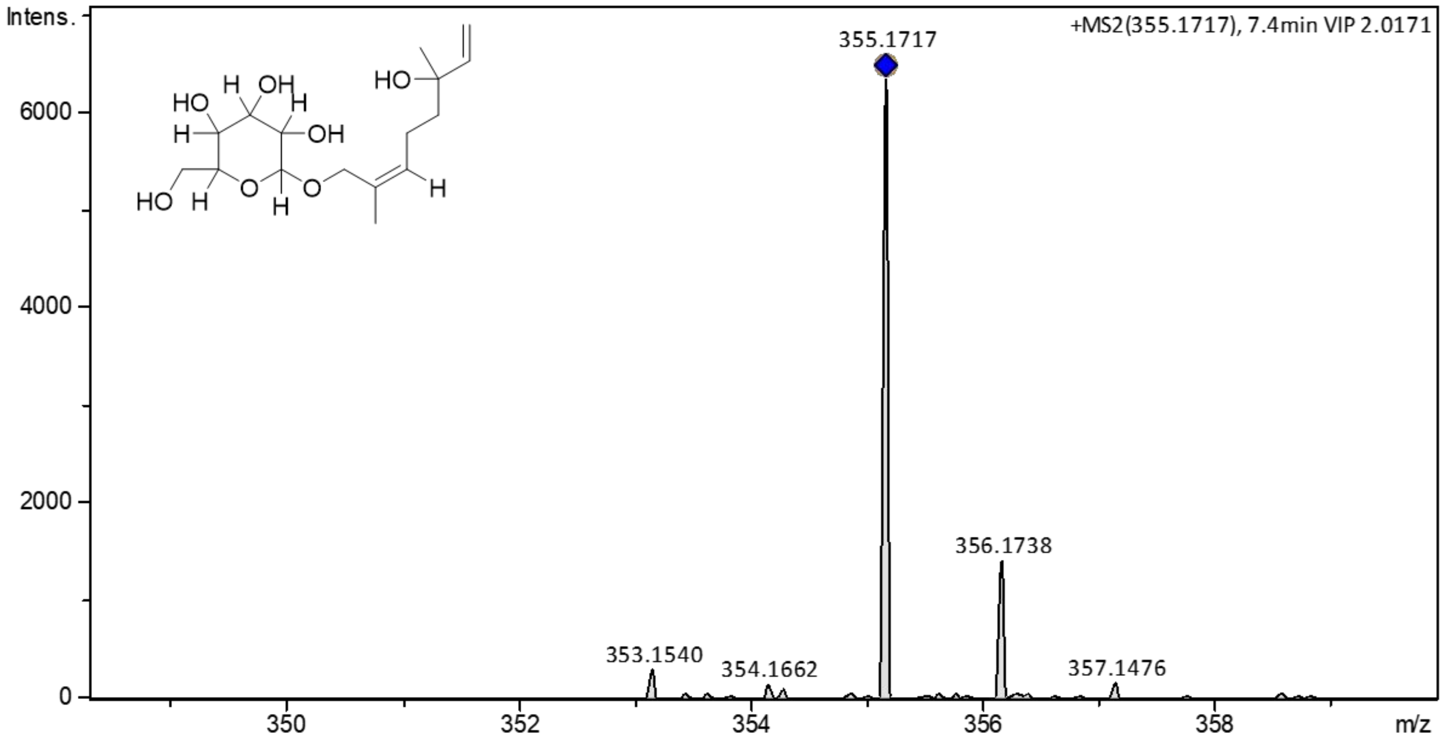

Figure SI 12. Mass spectrum of metabolite **12**.

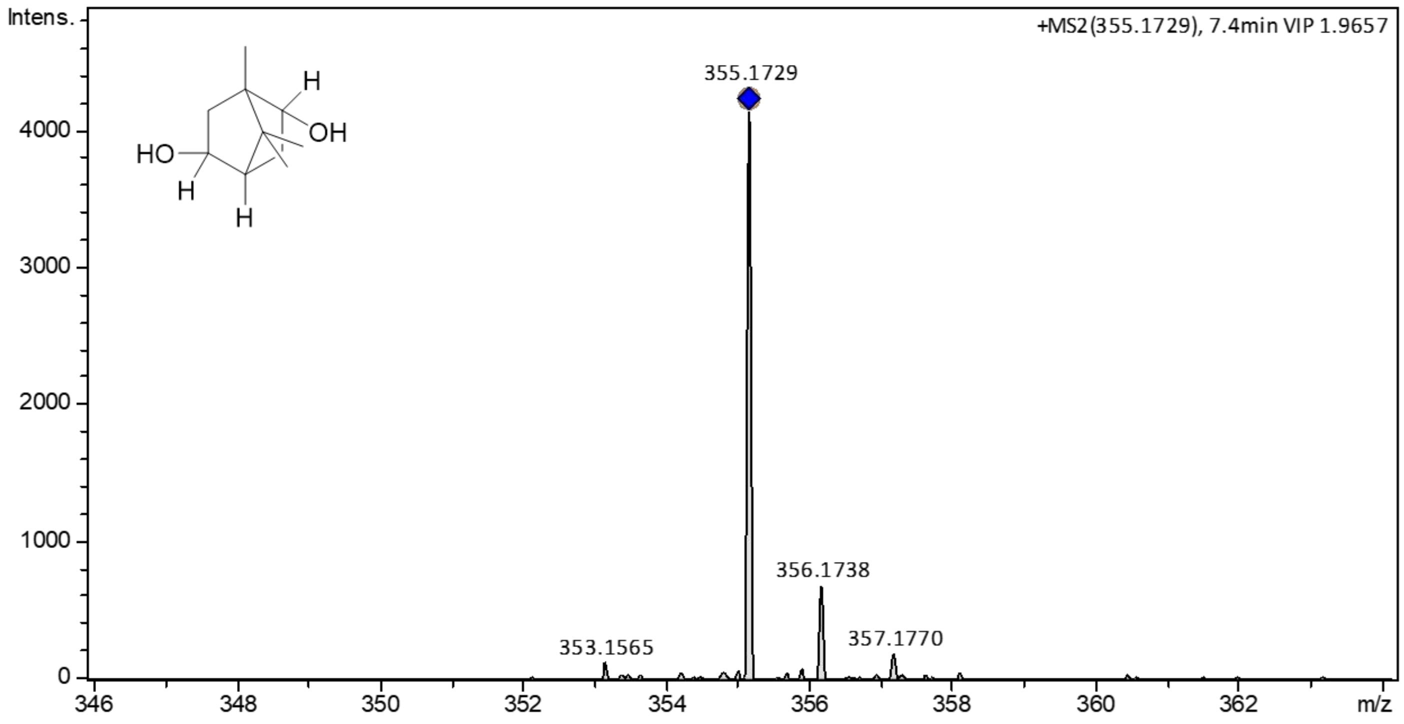

Figure SI 13. Mass spectrum of metabolite **13**.

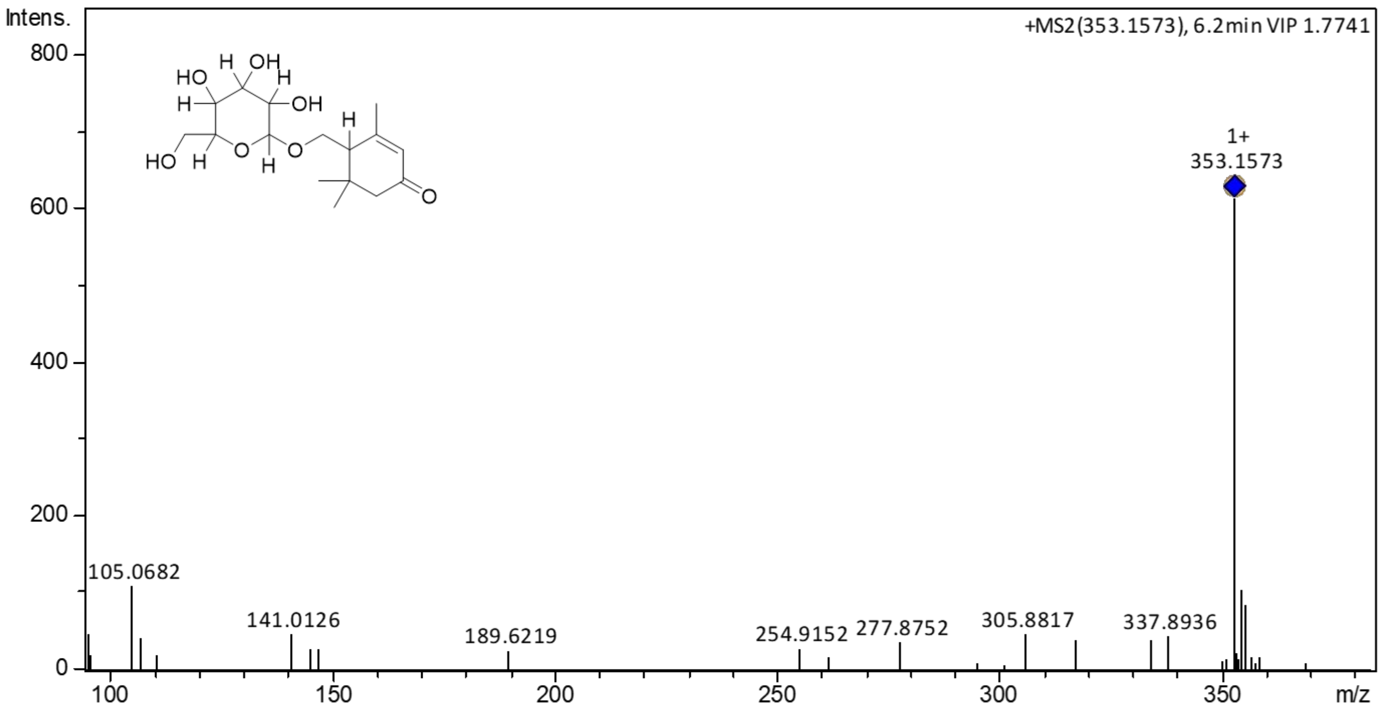

Figure SI 14. Mass spectrum of metabolite **14**.

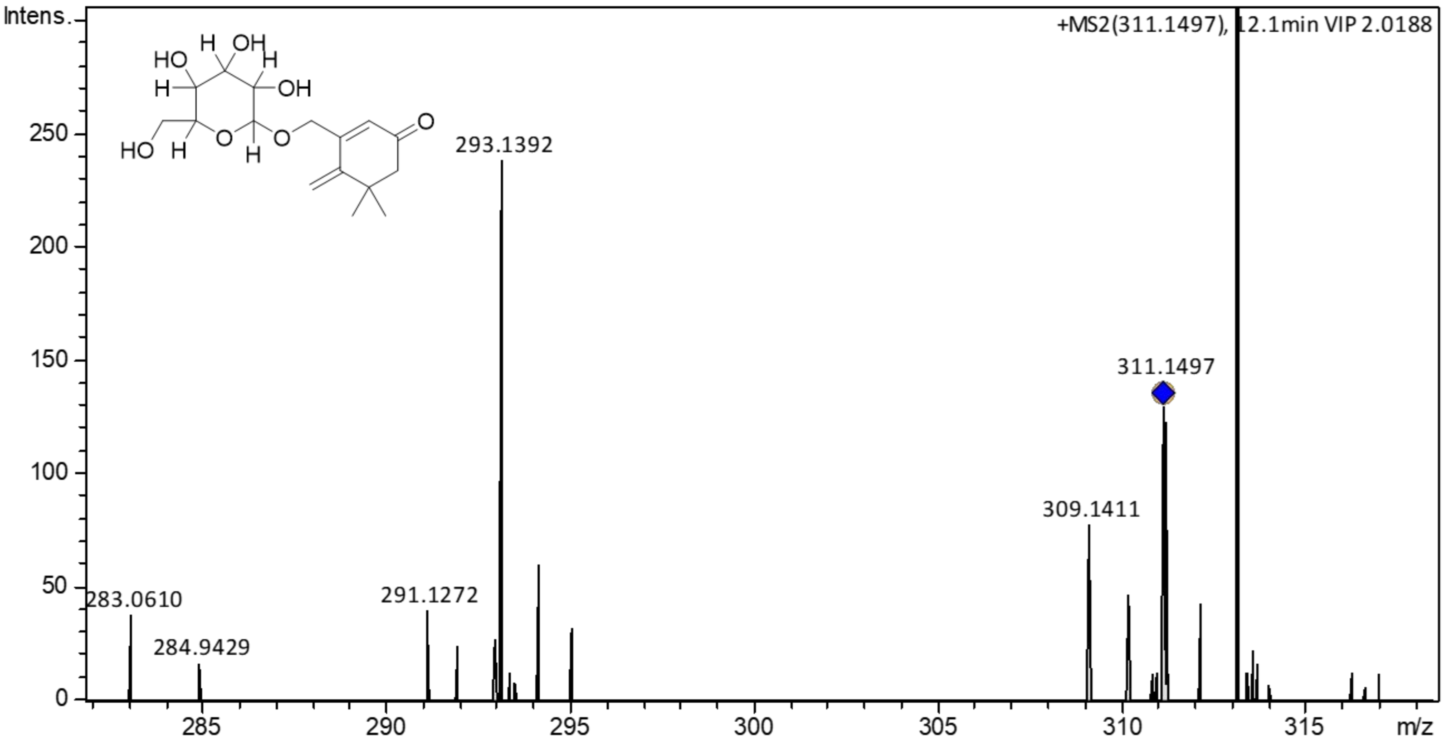

Figure SI 15. Mass spectrum of metabolite **15**.

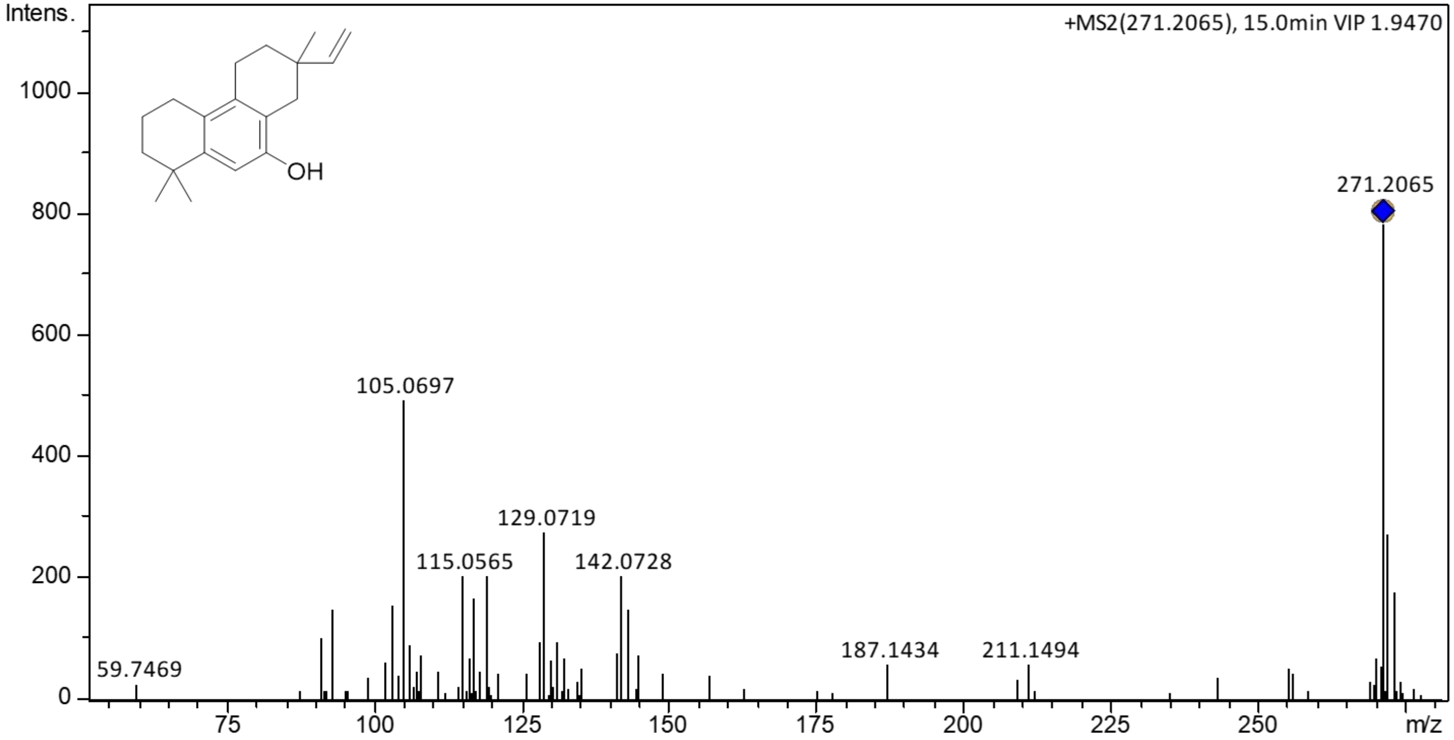

Figure SI 16. Mass spectrum of metabolite **16**.

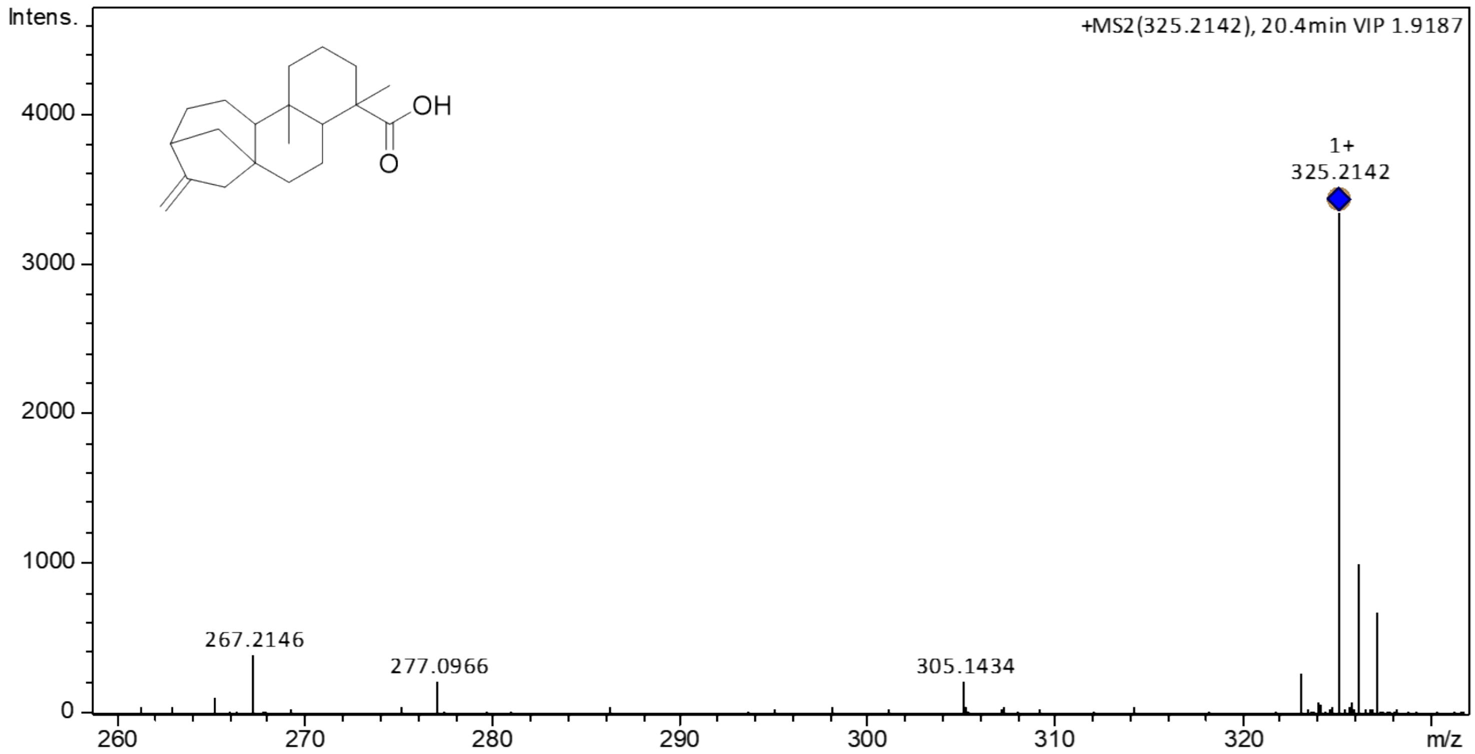

Figure SI 17. Mass spectrum of metabolite **17**.

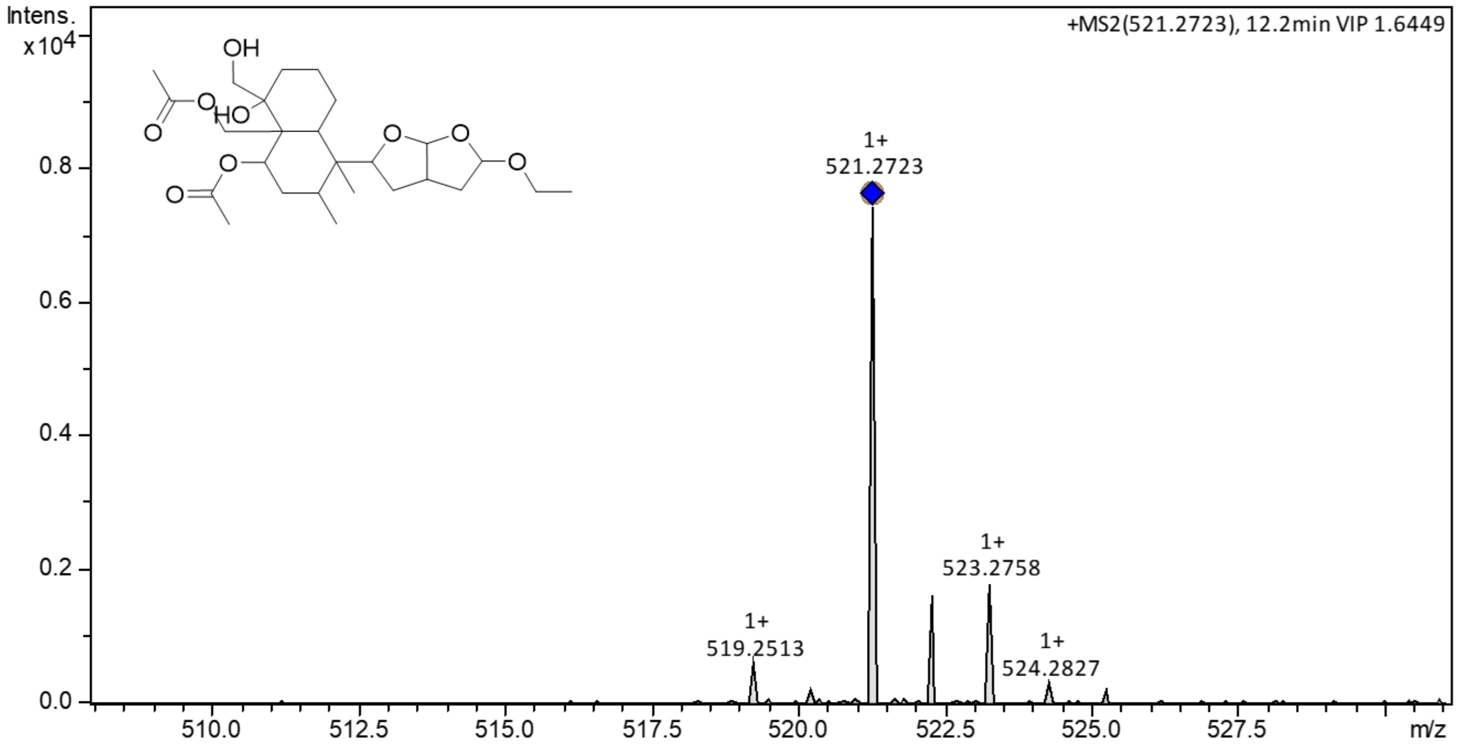

Figure SI 18. Mass spectrum of metabolite **18**.

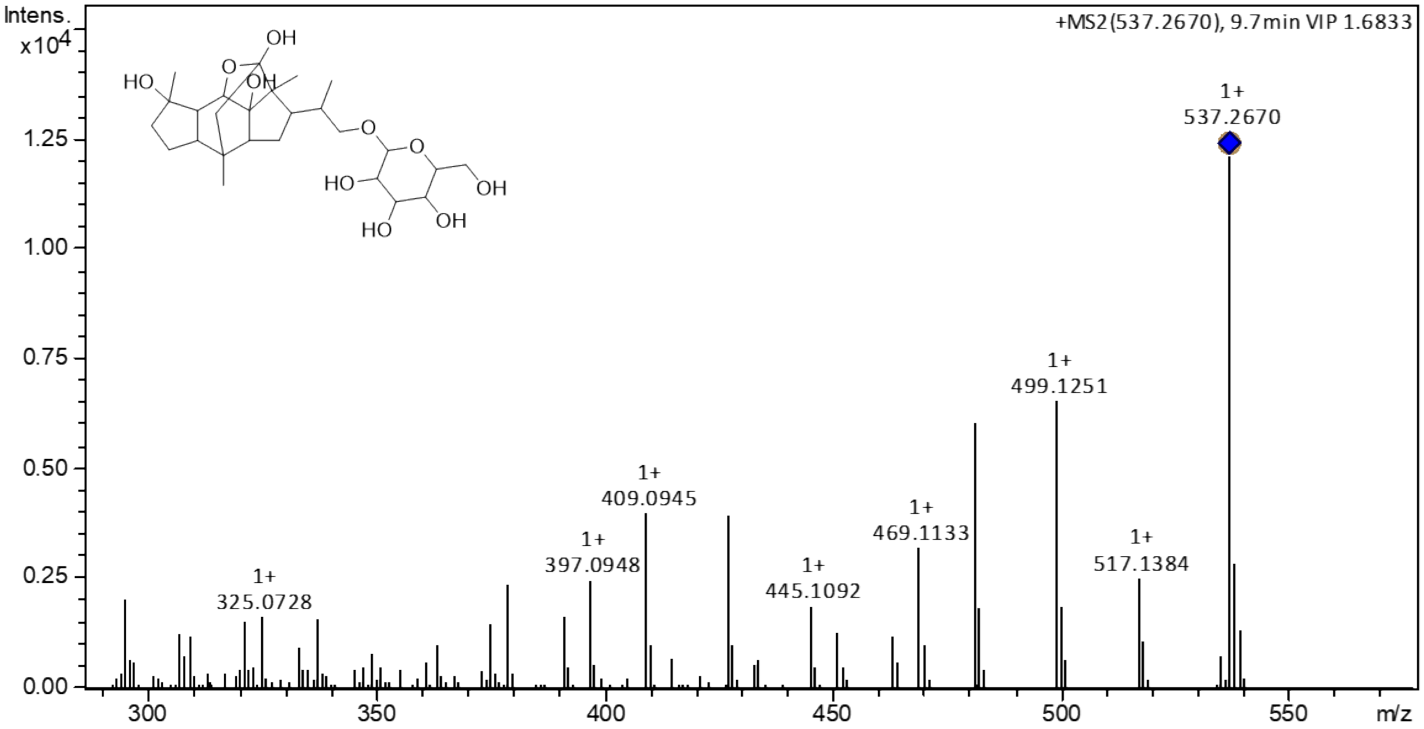

Figure SI 19. Mass spectrum of metabolite **19**.

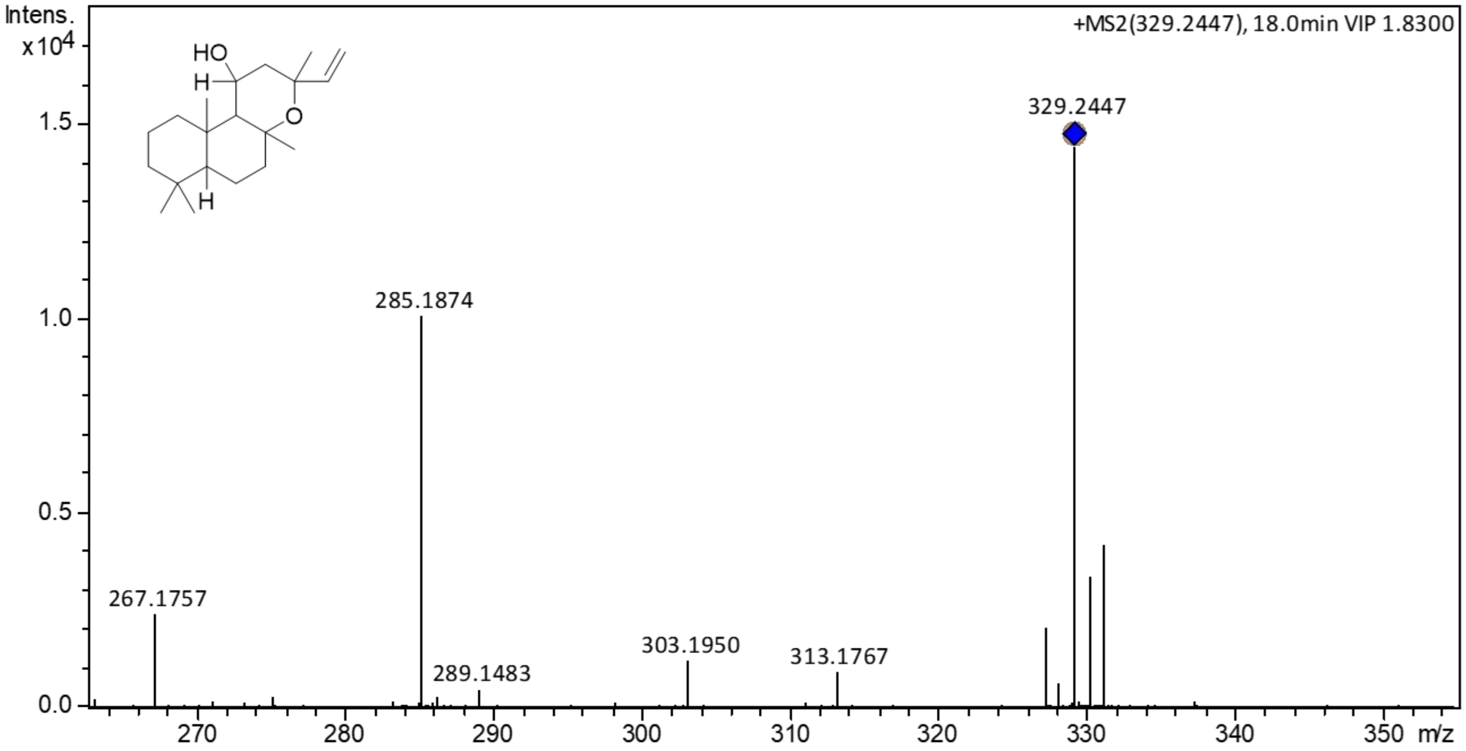

Figure SI 20. Mass spectrum of metabolite **20**.

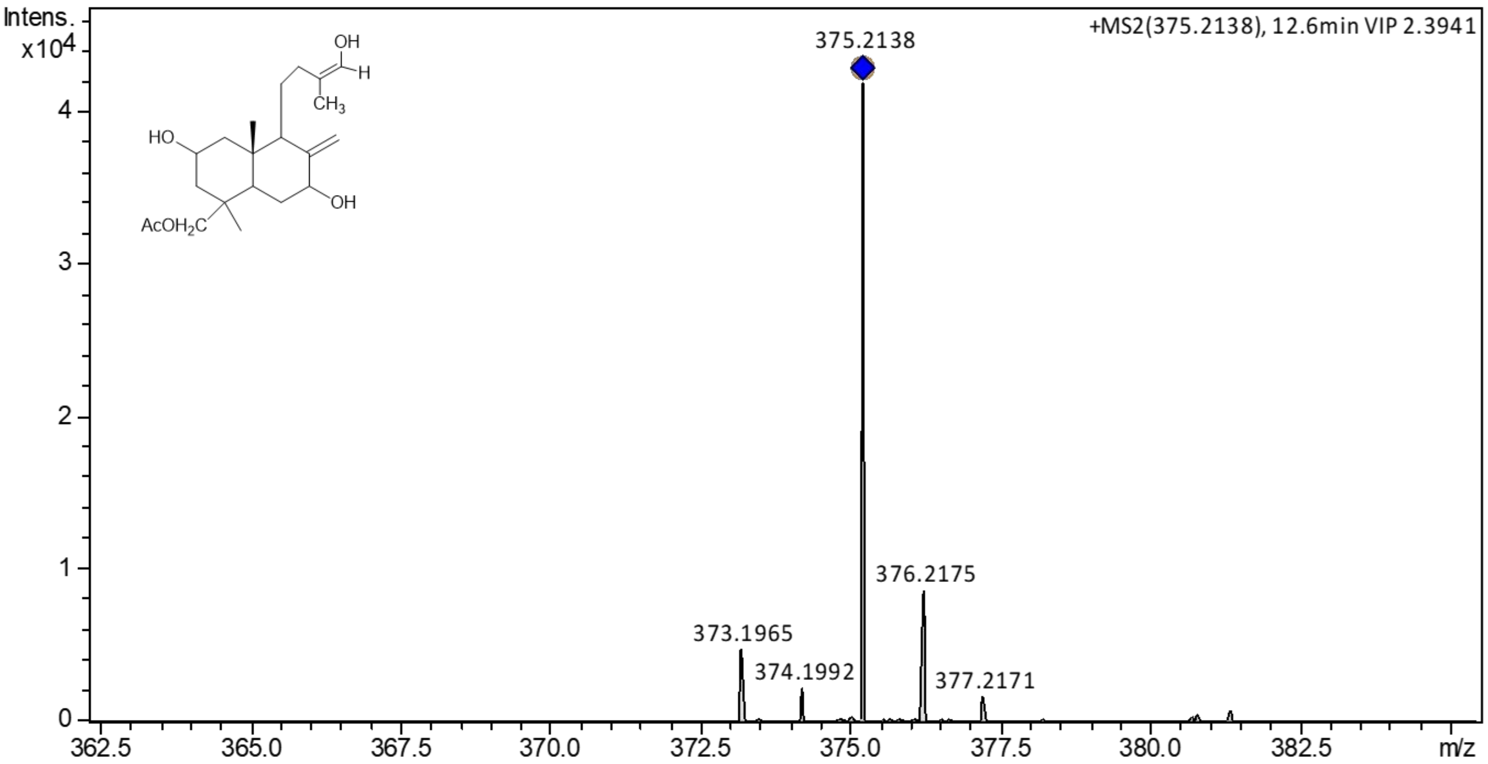

Figure SI 21. Mass spectrum of metabolite **21**.

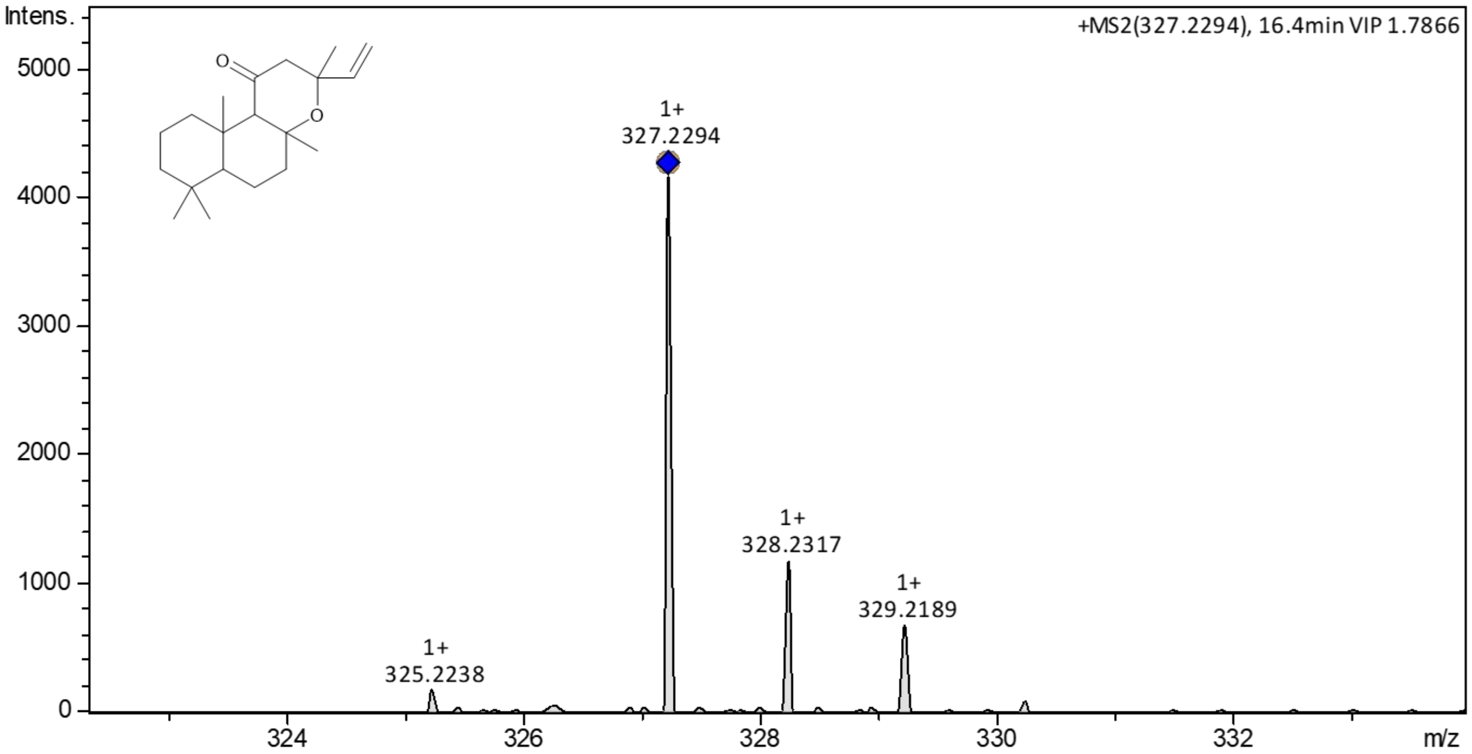

Figure SI 22. Mass spectrum of metabolite **22**.

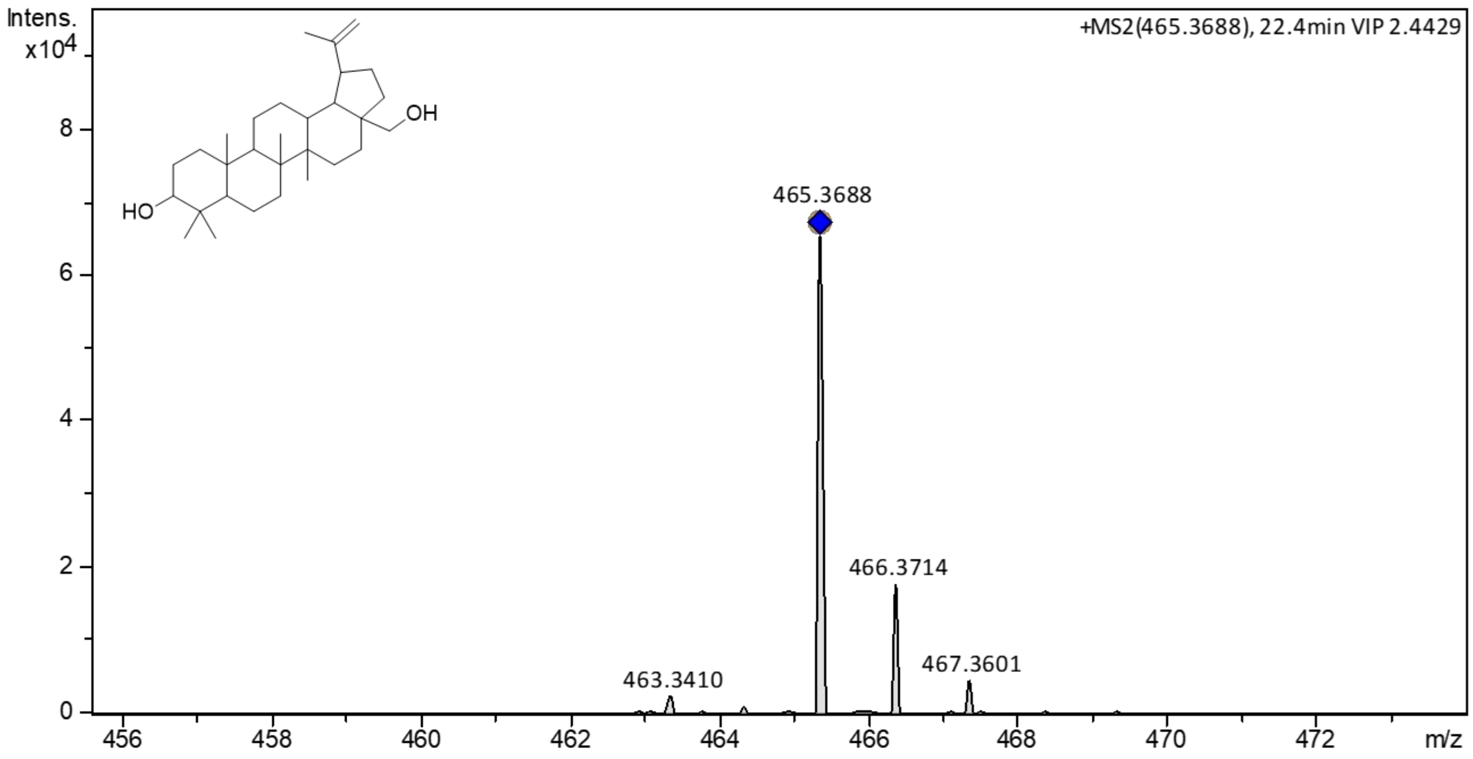

Figure SI 23. Mass spectrum of metabolite **23**.

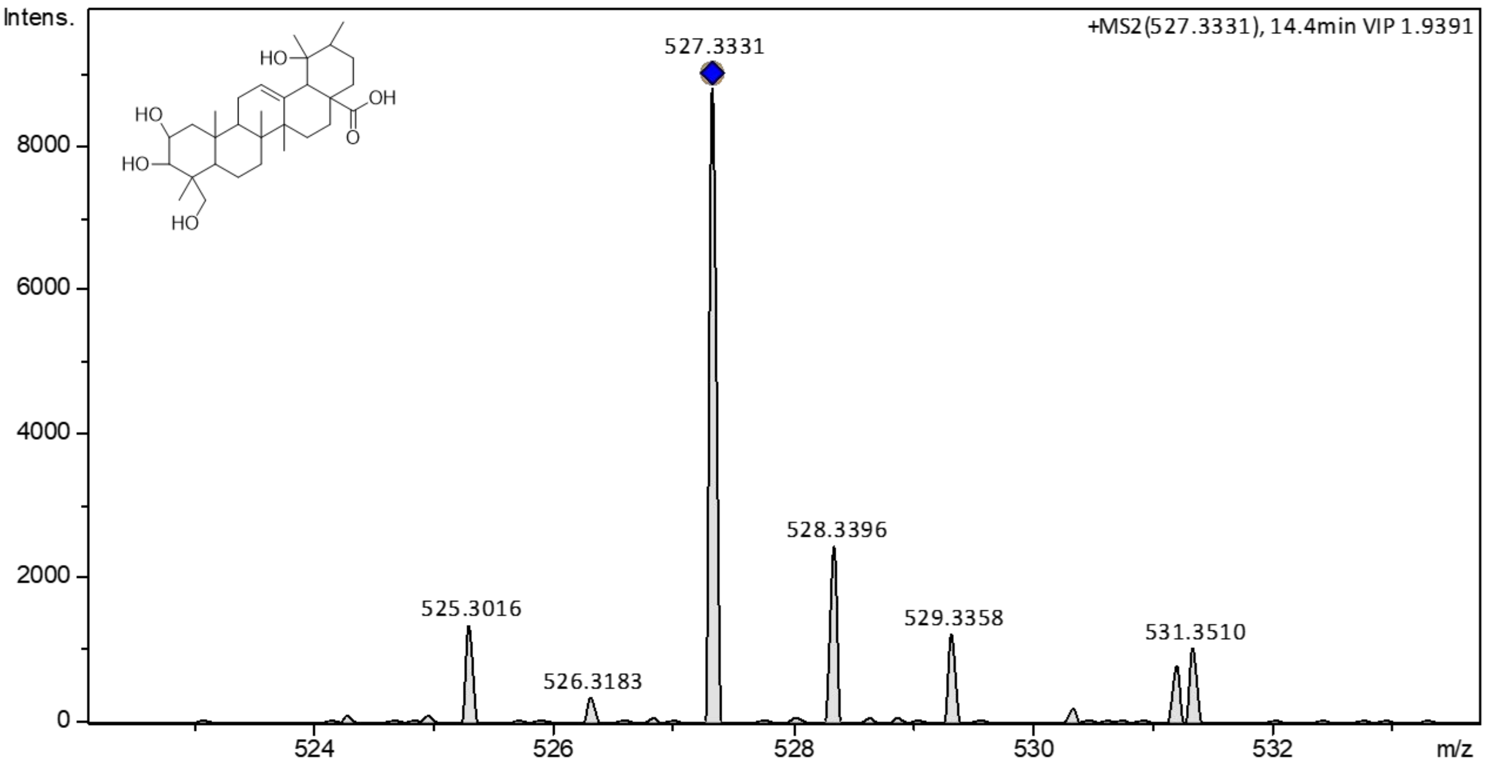

Figure SI 24. Mass spectrum of metabolite **24**.

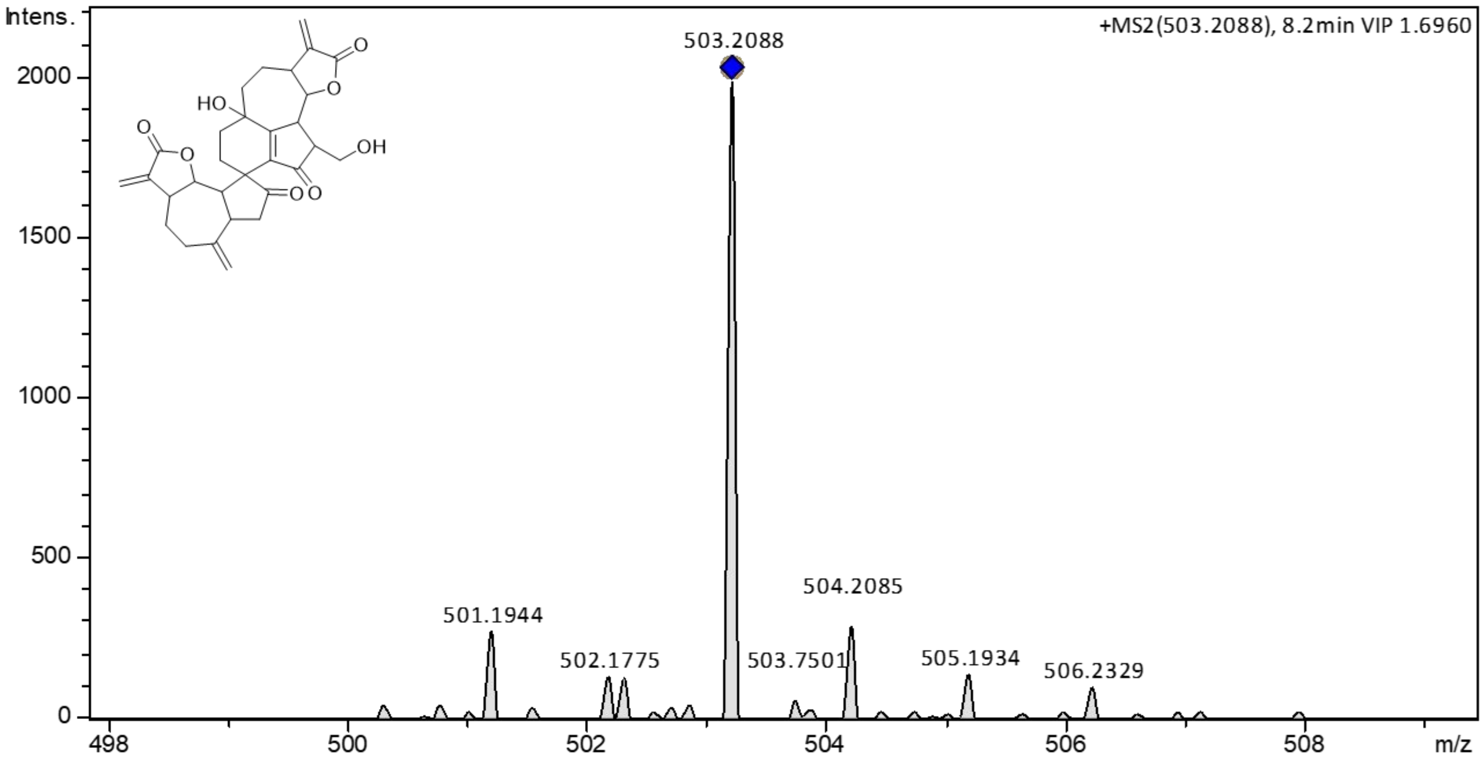

Figure SI 25. Mass spectrum of metabolite **25**.

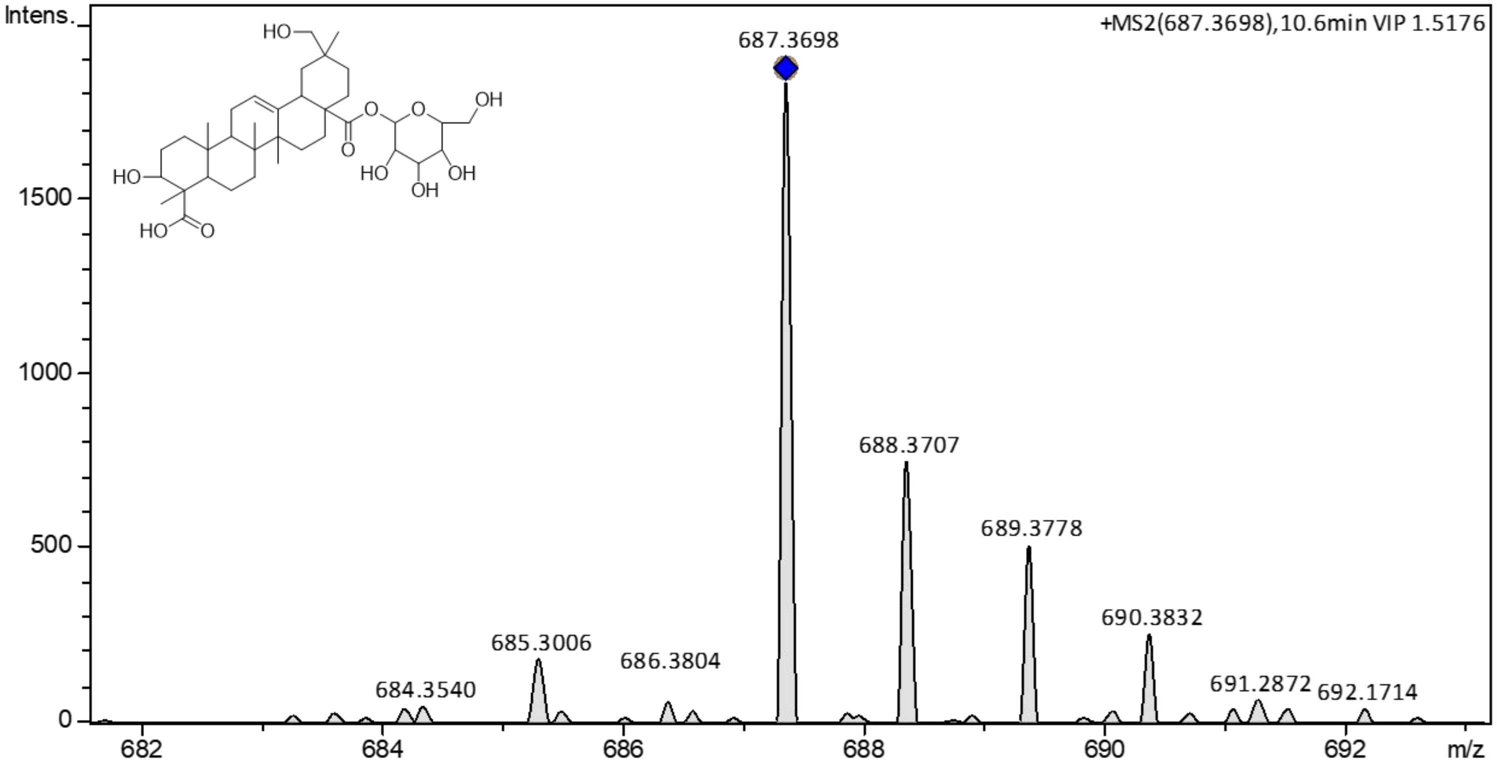

Figure SI 26. Mass spectrum of metabolite **26**.

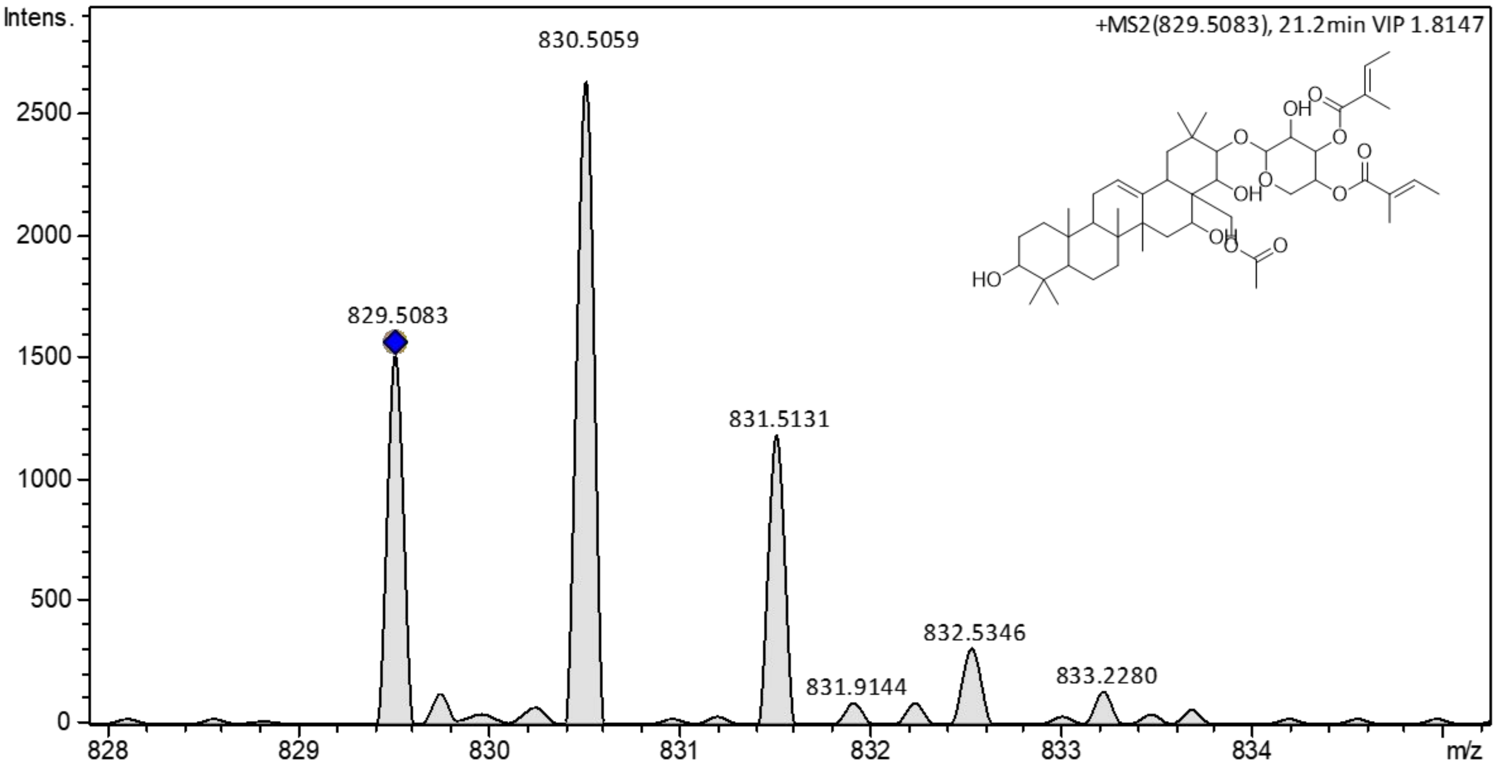

Figure SI 27. Mass spectrum of metabolite **27**.

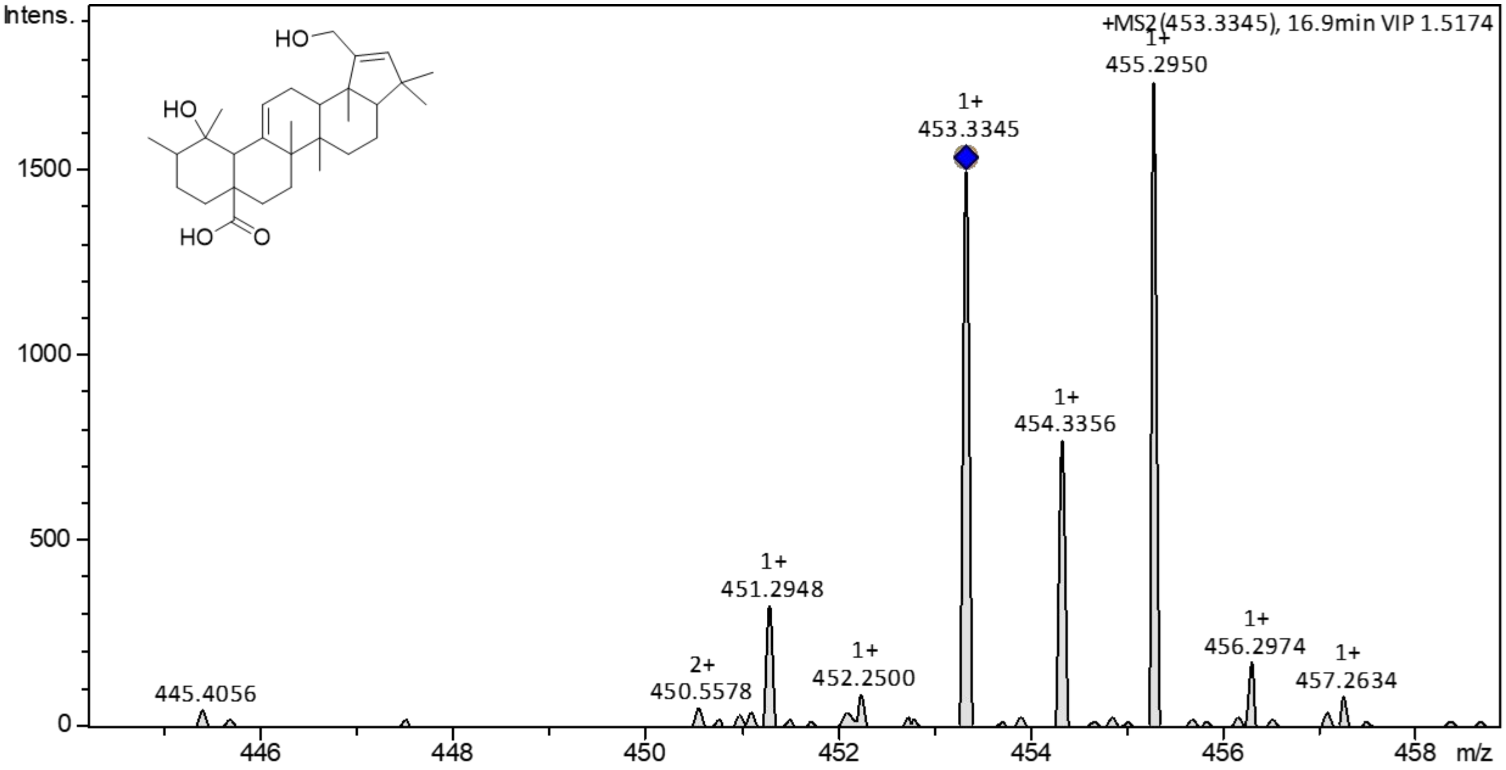

Figure SI 28. Mass spectrum of metabolite **28**.

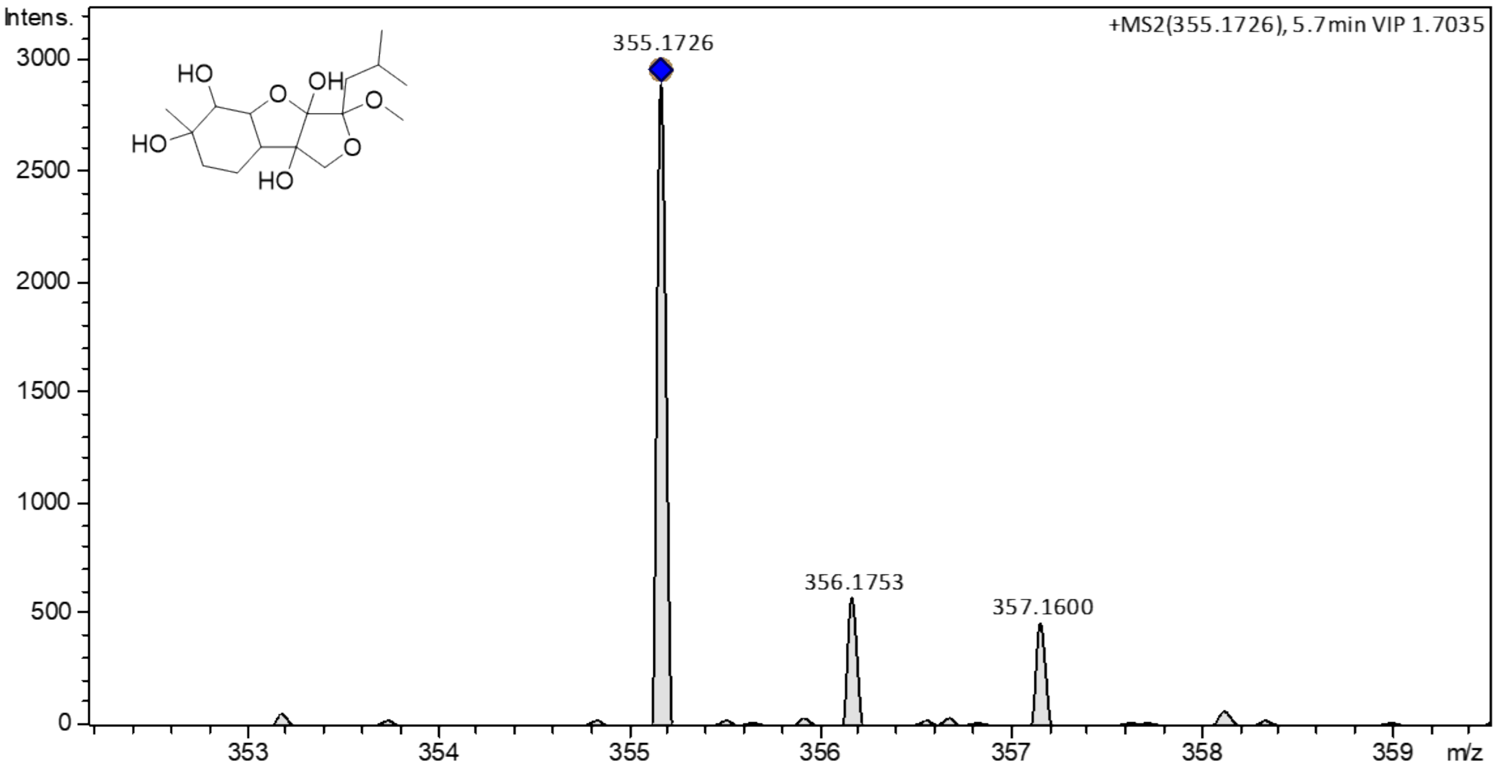

Figure SI 29. Mass spectrum of metabolite **29**.

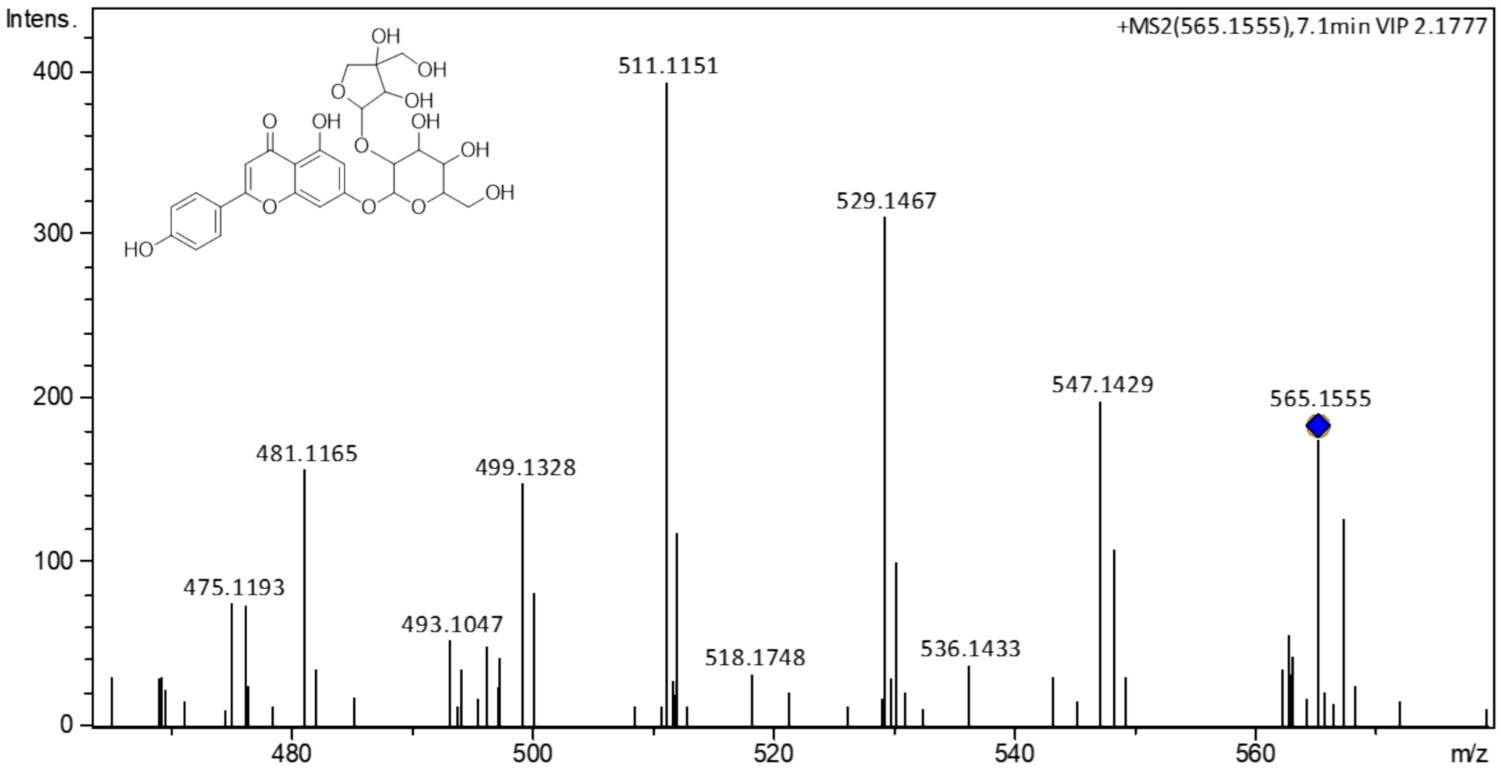

Figure SI 30. Mass spectrum of metabolite **30**.

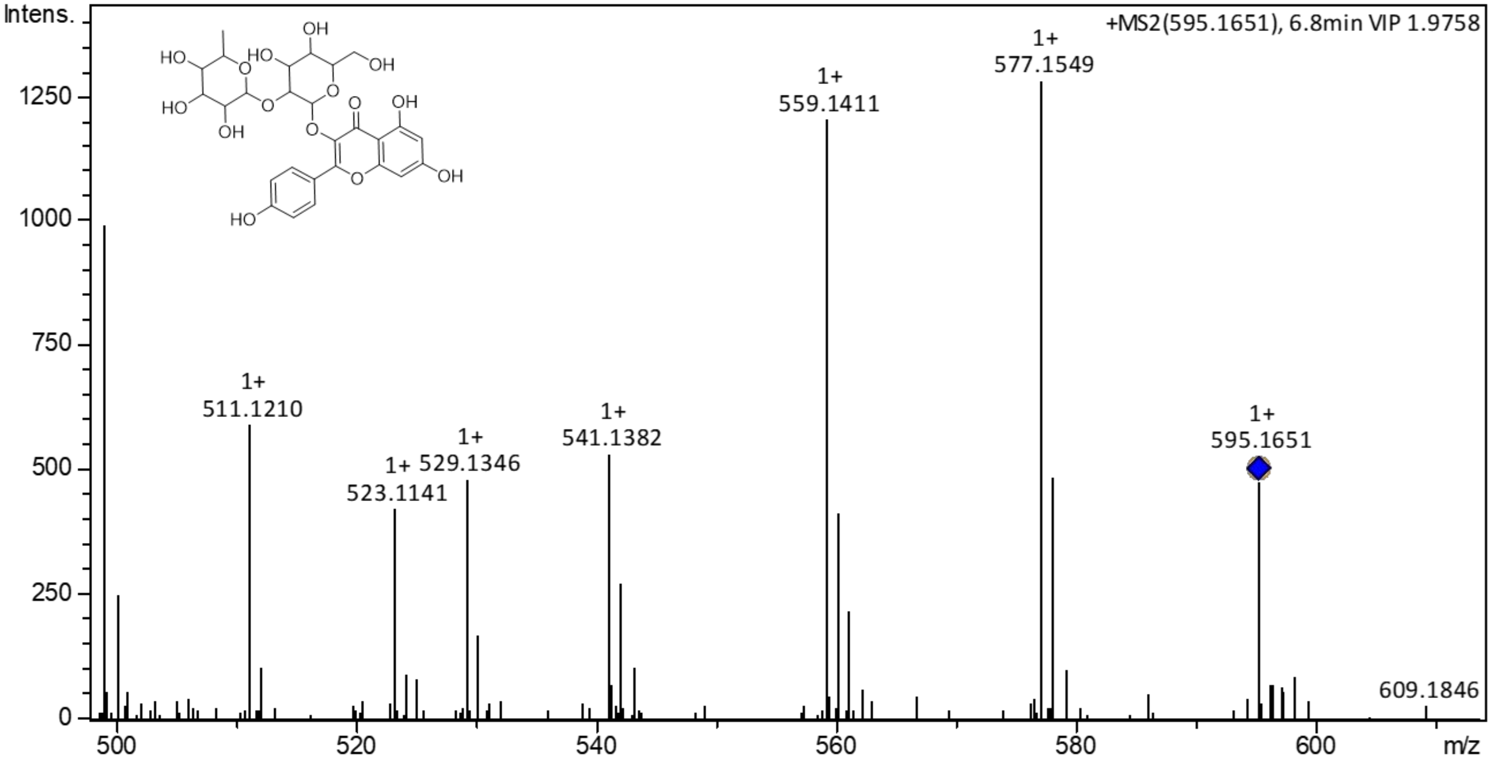

Figure SI 31. Mass spectrum of metabolite **31**.

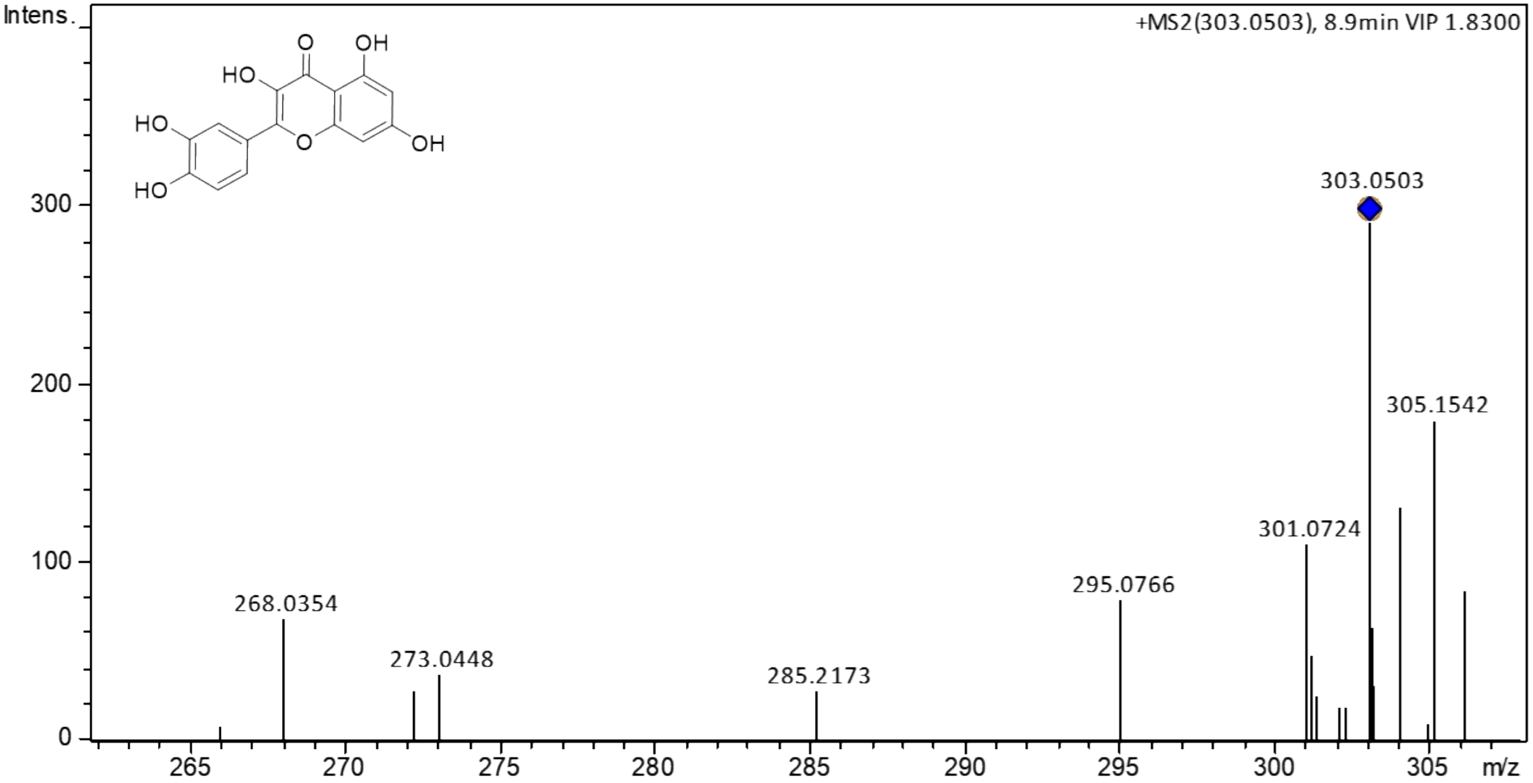

Figure SI 32. Mass spectrum of metabolite **32**.

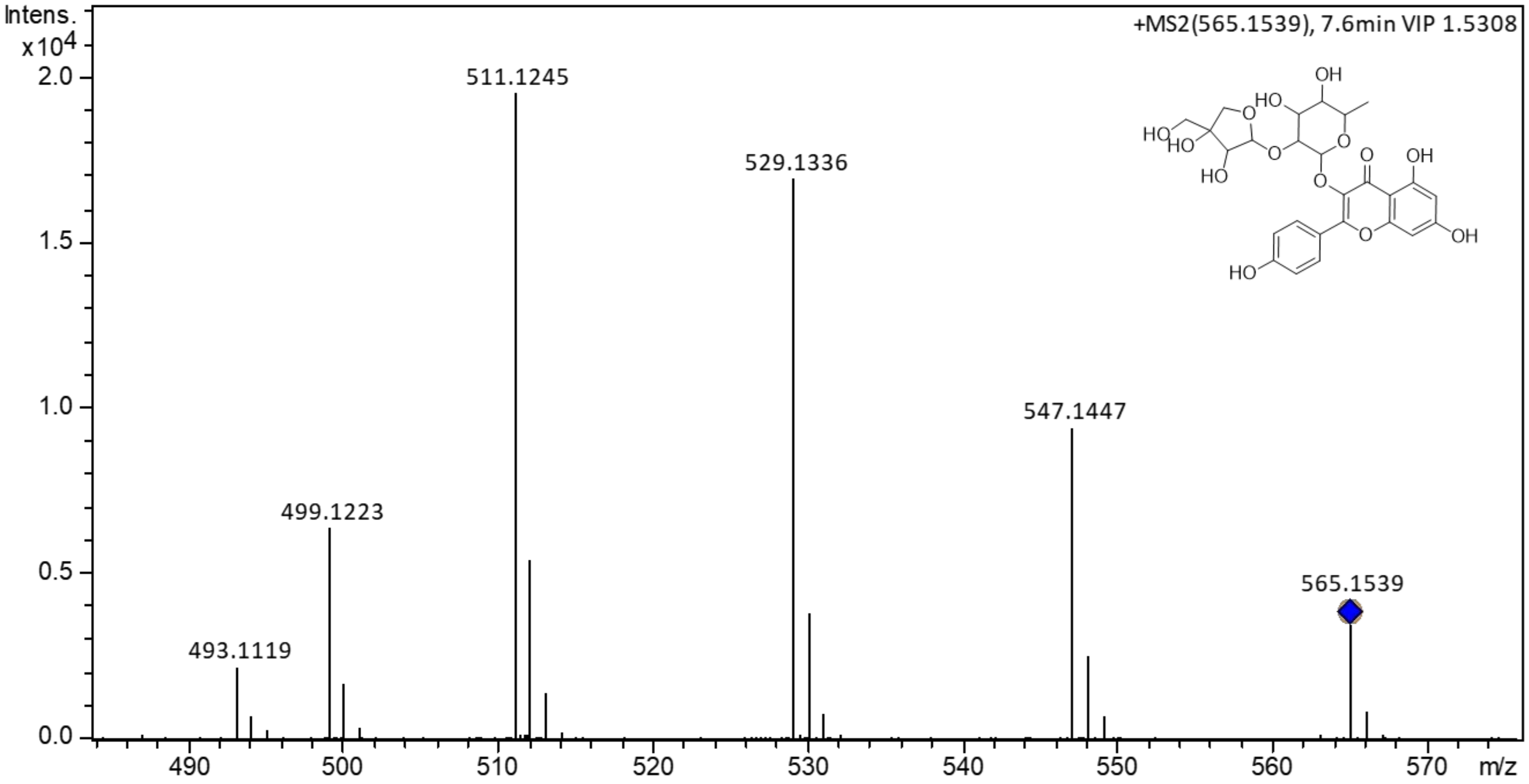

Figure SI 33. Mass spectrum of metabolite **33**.

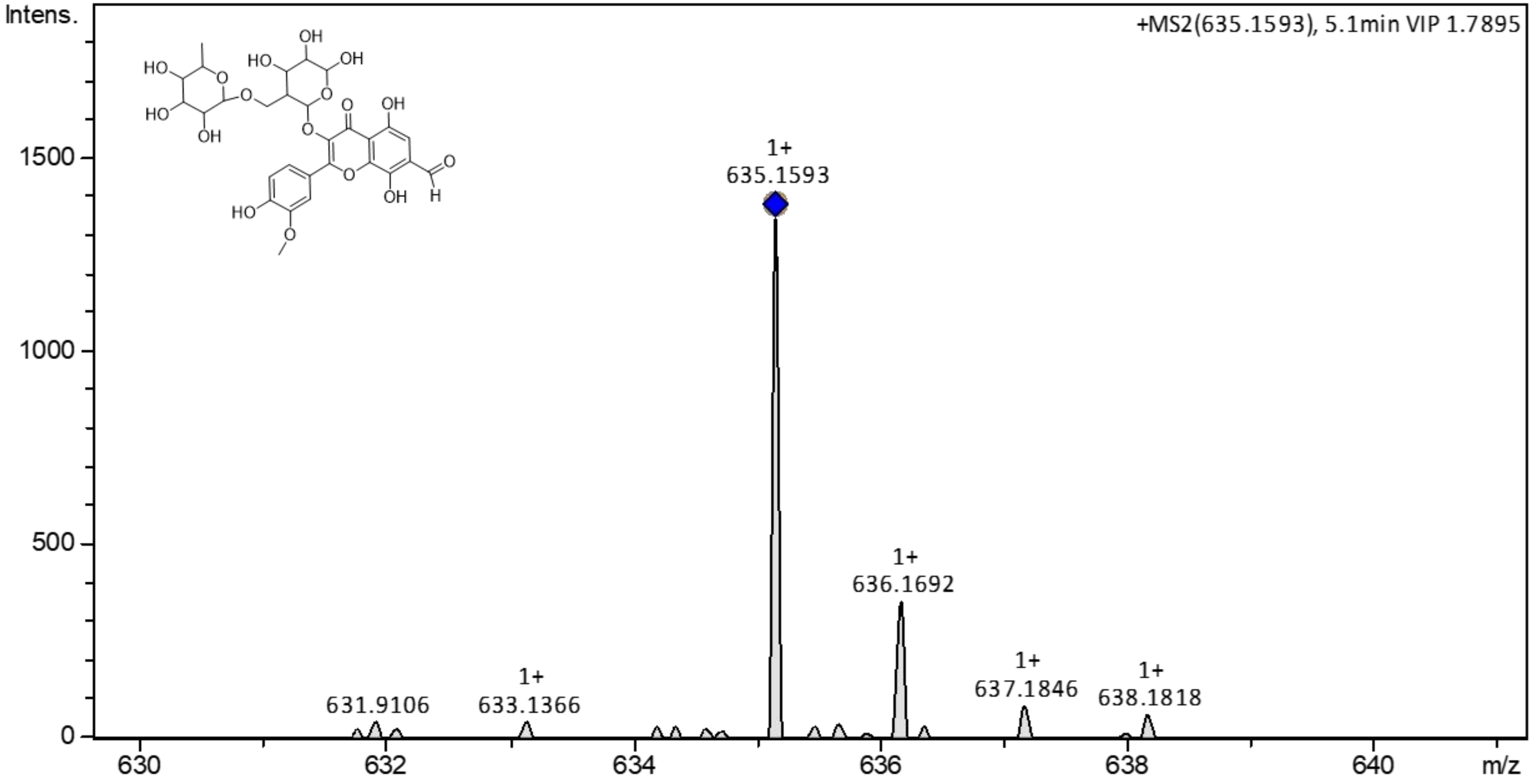

Figure SI 34. Mass spectrum of metabolite **34**.

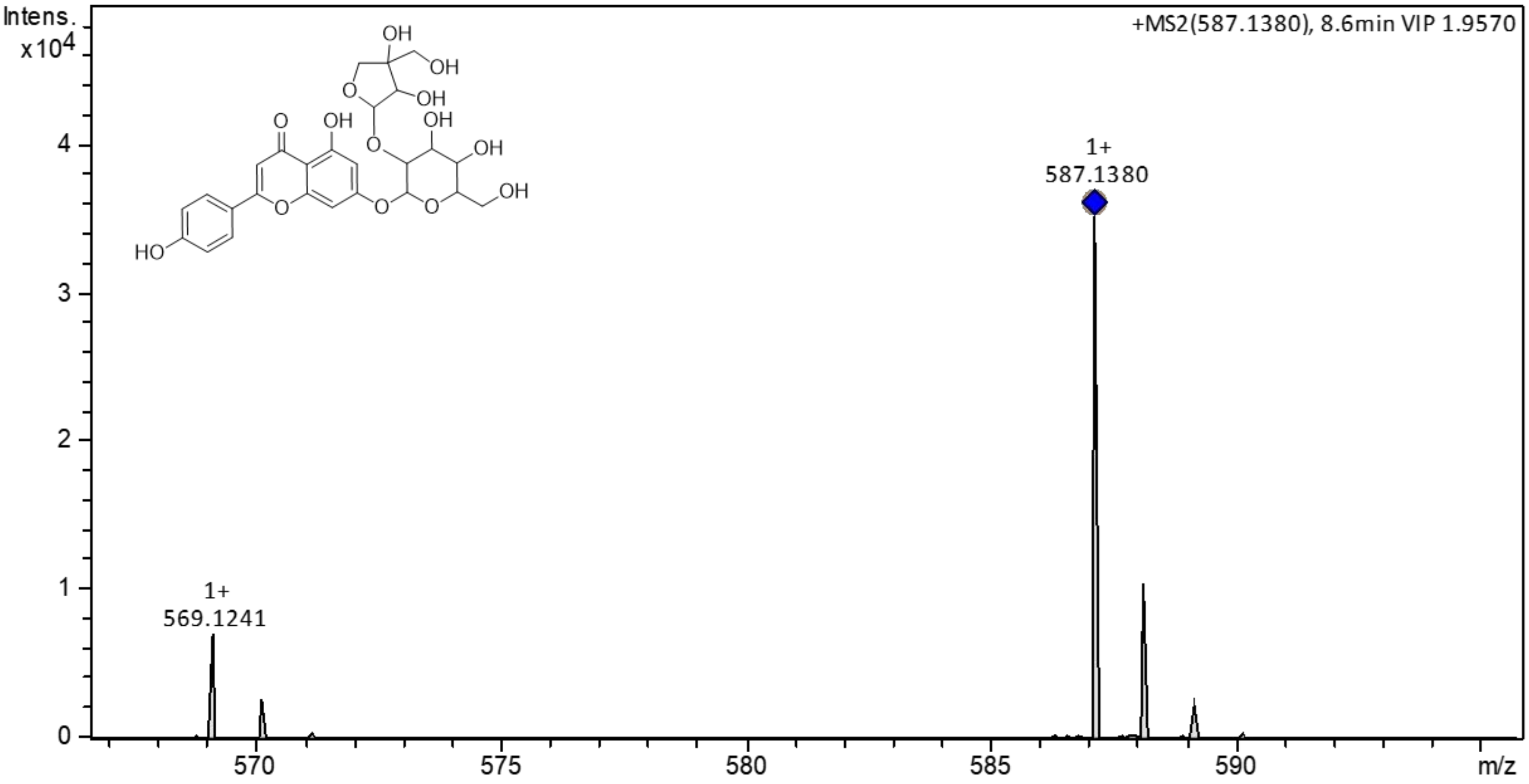

Figure SI 35. Mass spectrum of metabolite **35**.

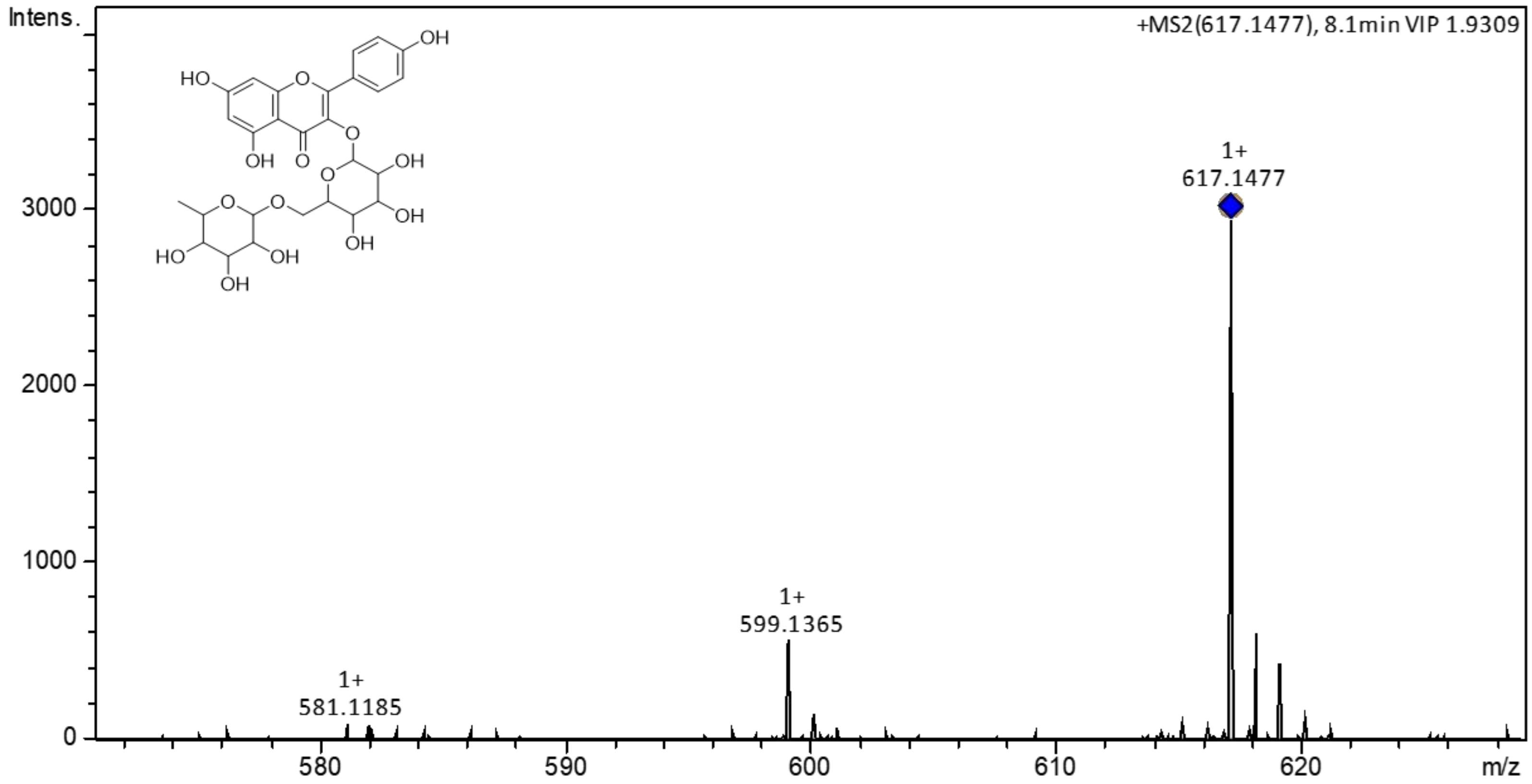

Figure SI 36. Mass spectrum of metabolite **36**.

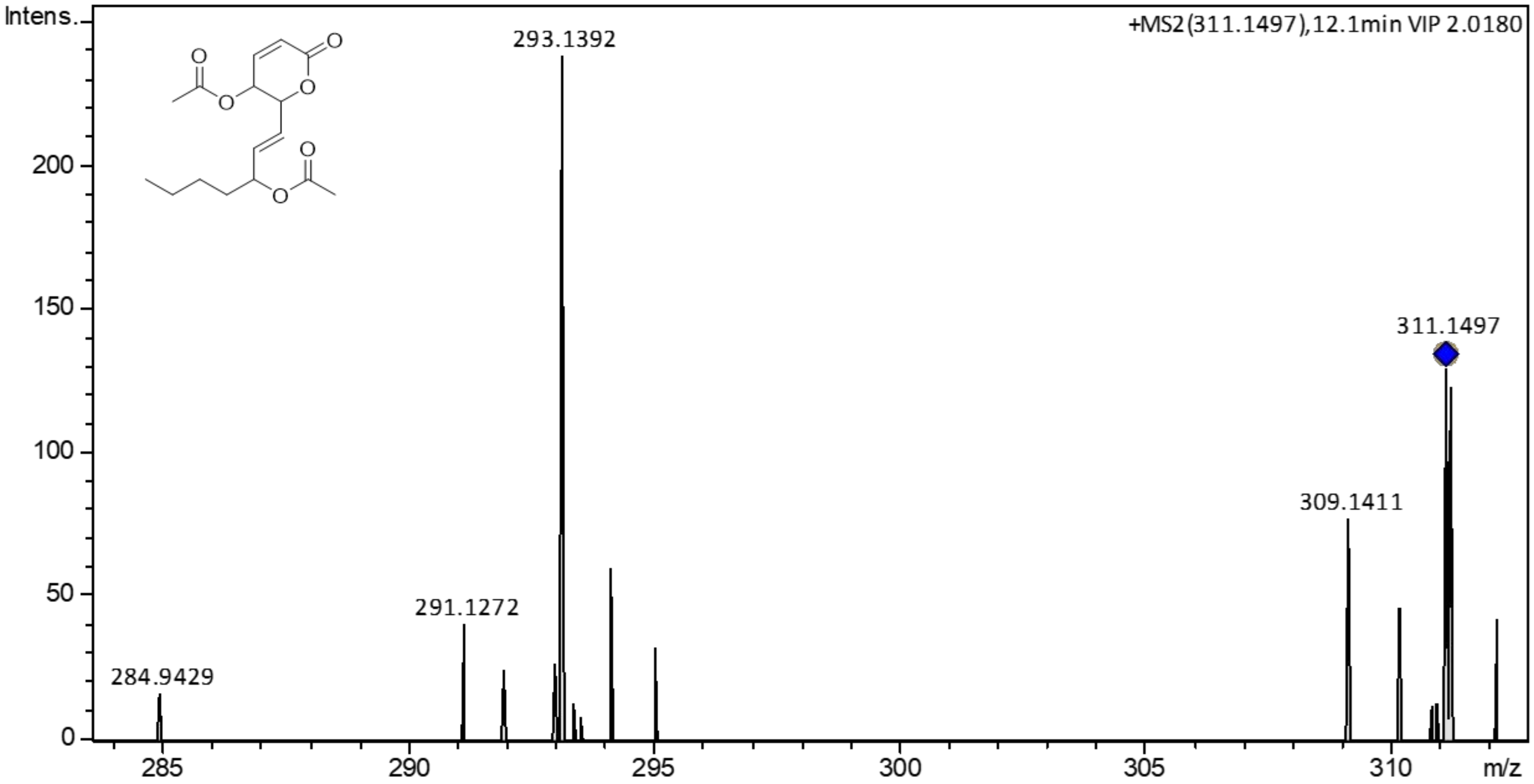

Figure SI 37. Mass spectrum of metabolite **37**.

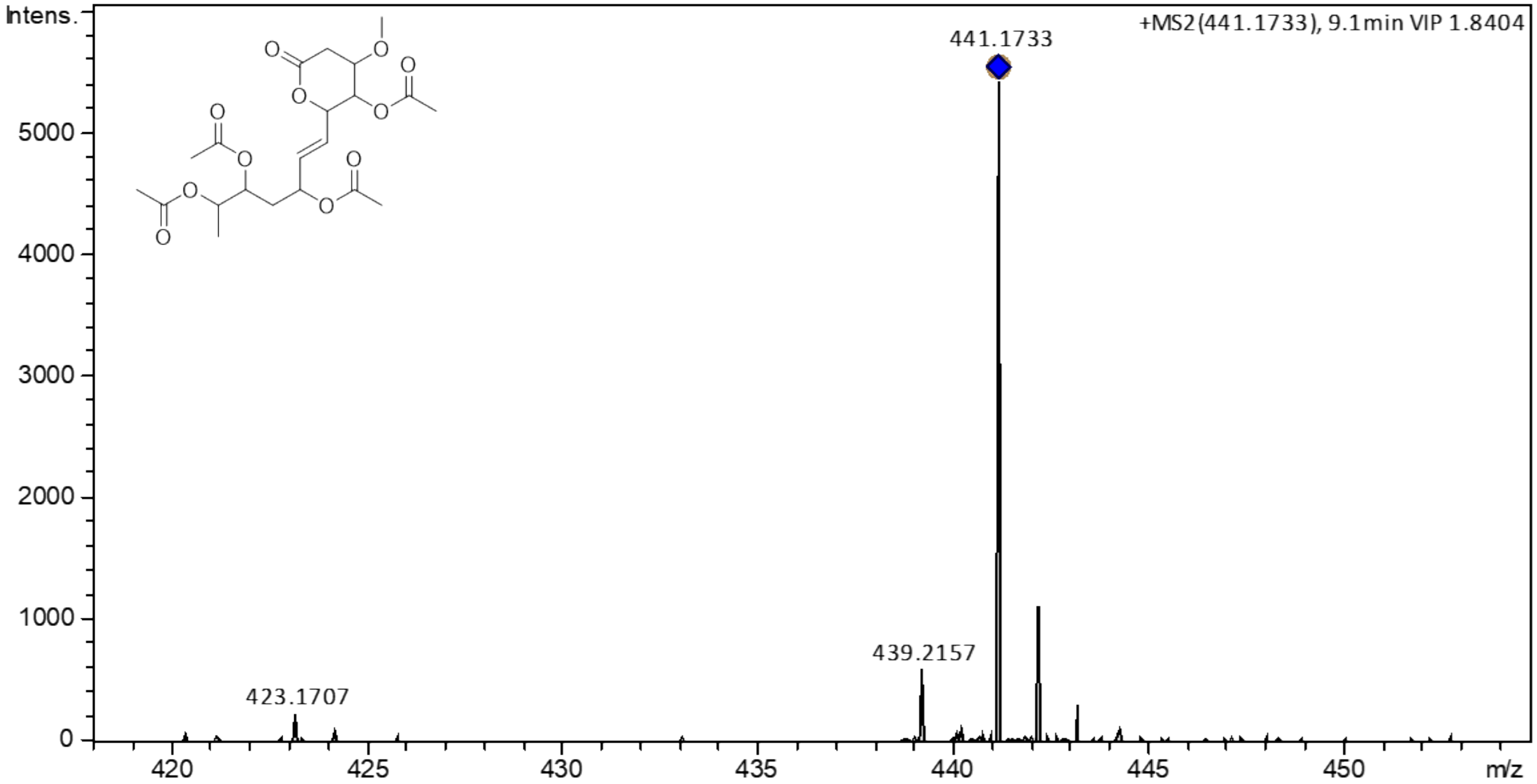

Figure SI 38. Mass spectrum of metabolite **38**.

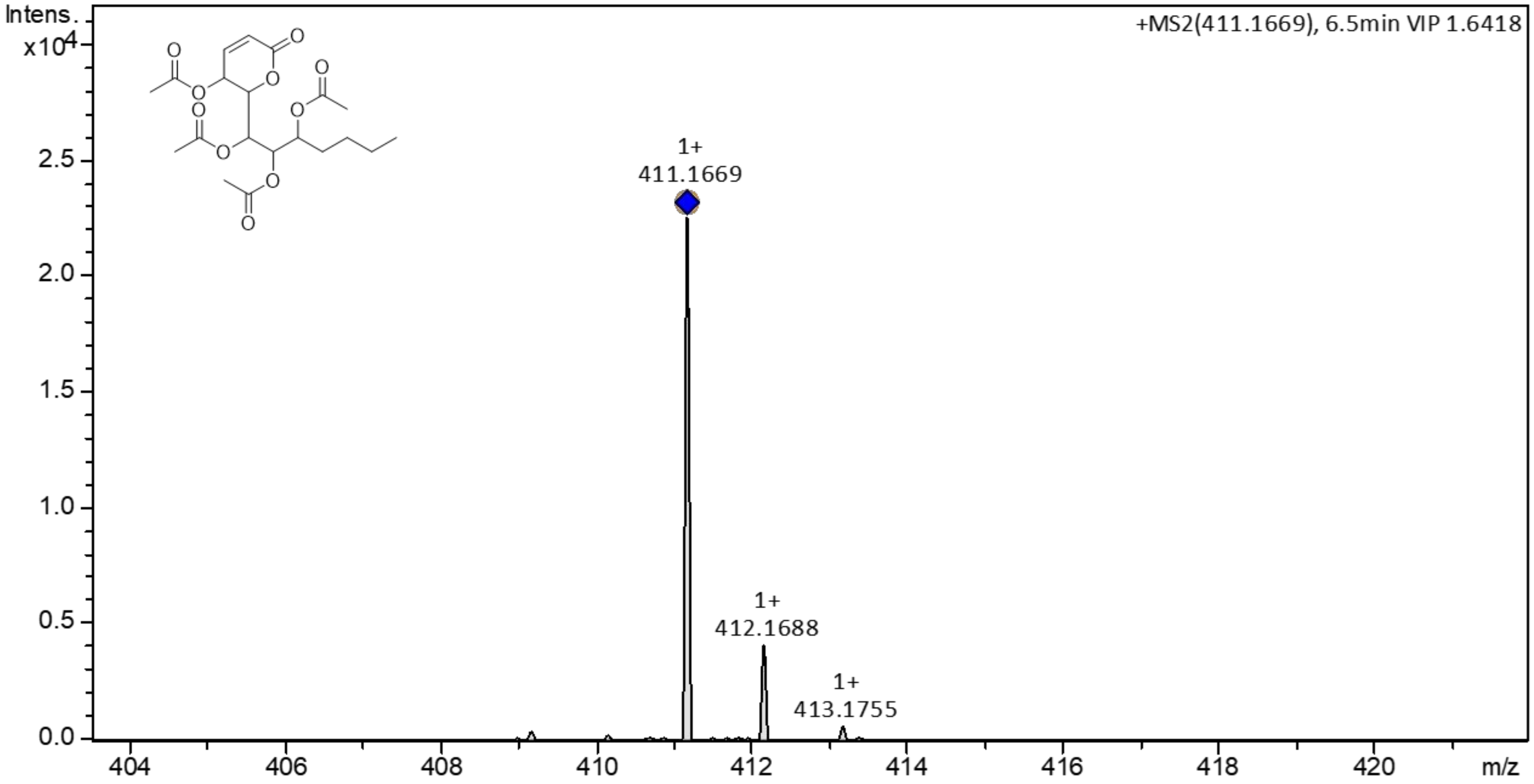

Figure SI 39. Mass spectrum of metabolite **39**.

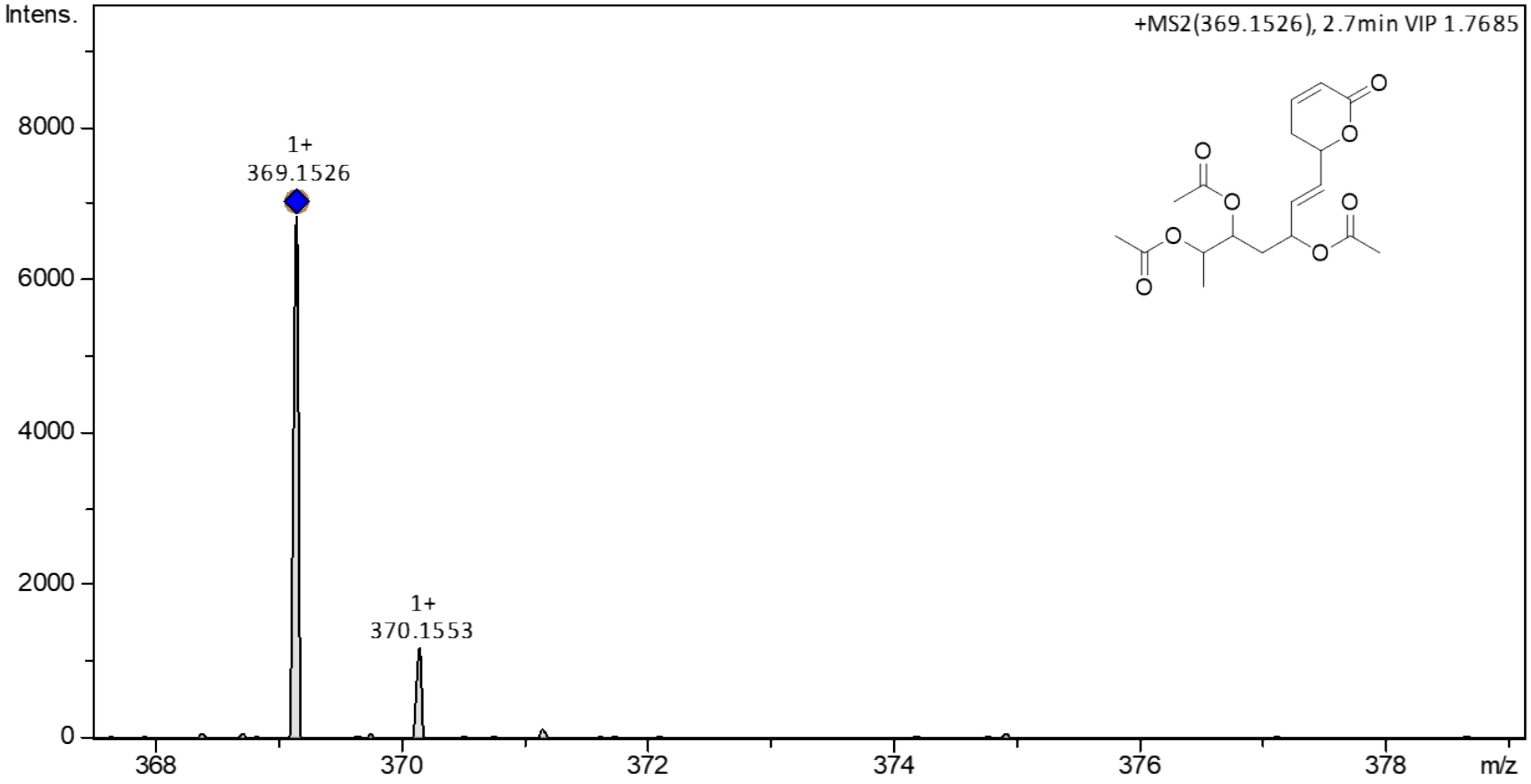

Figure SI 40. Mass spectrum of metabolite **40**.

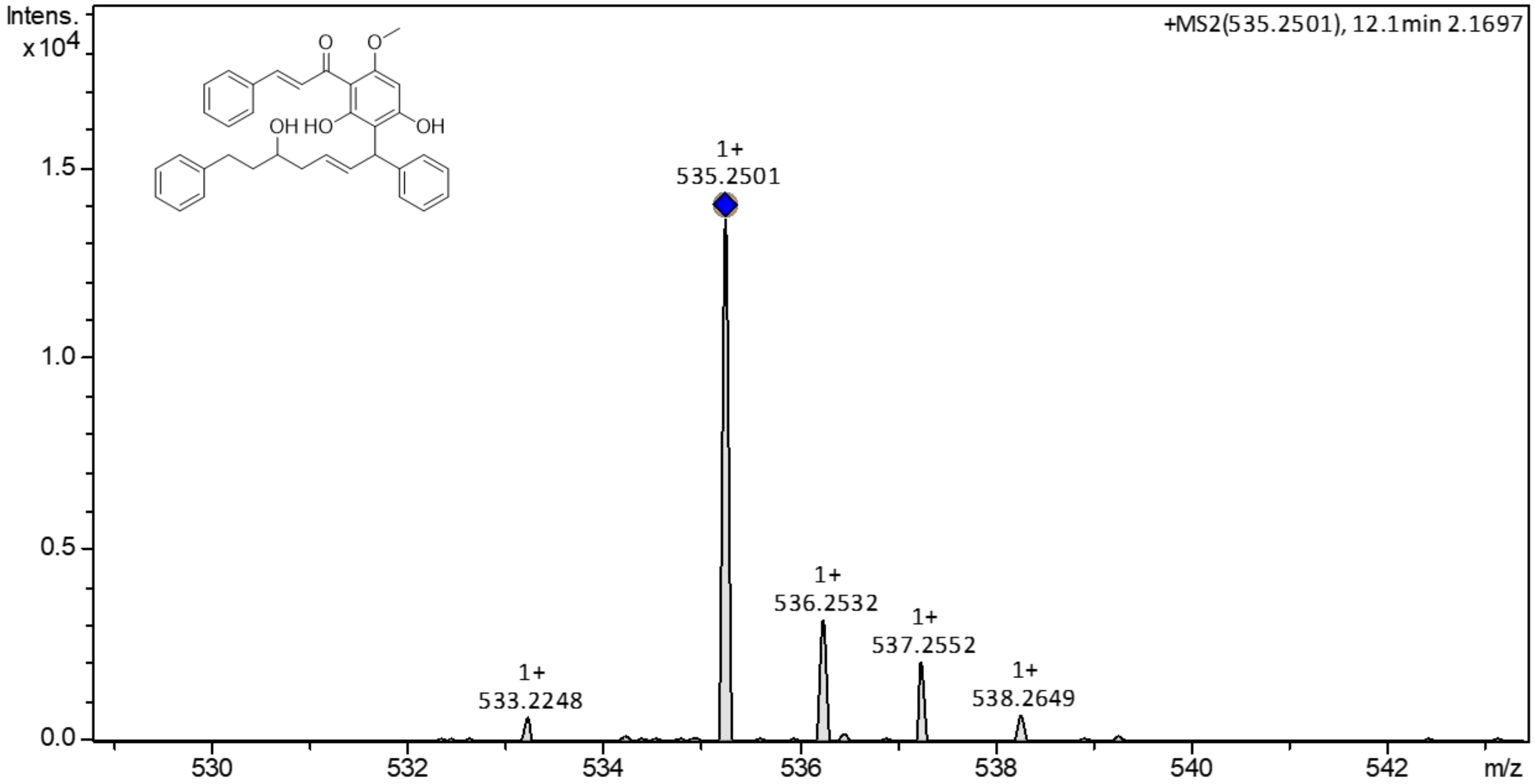

Figure SI 41. Mass spectrum of metabolite **41**.

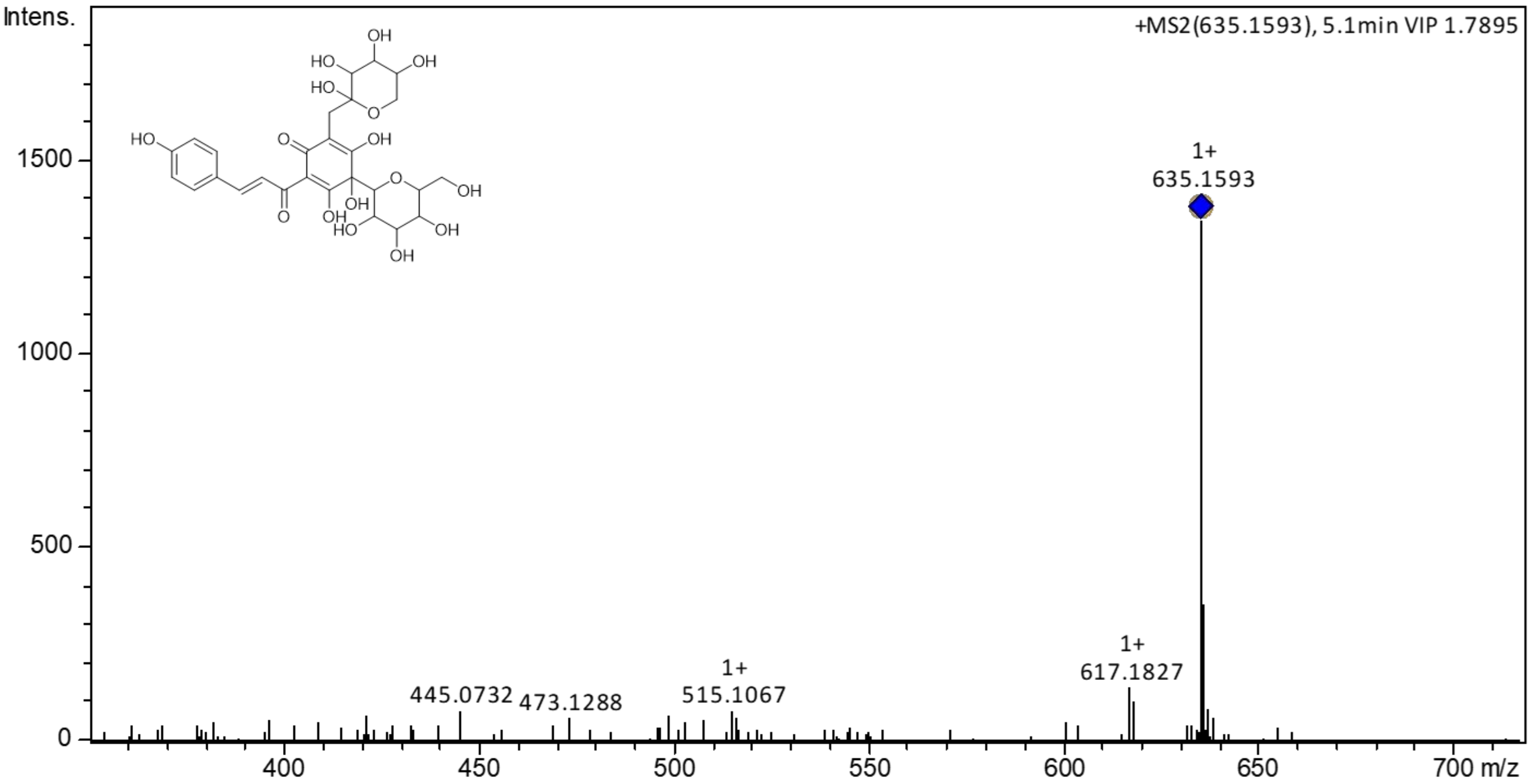

Figure SI 42. Mass spectrum of metabolite **42**.

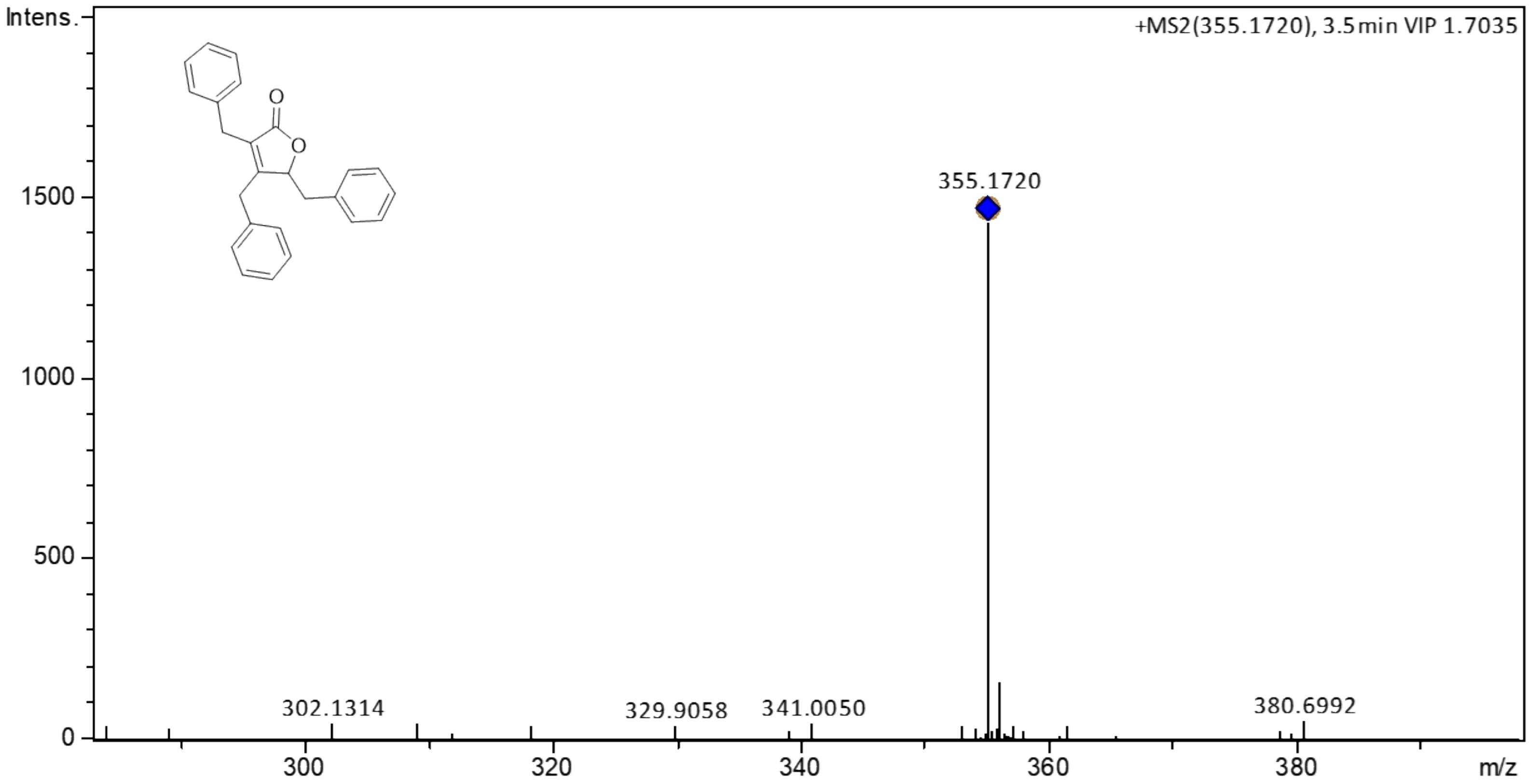

Figure SI 43. Mass spectrum of metabolite **43**.

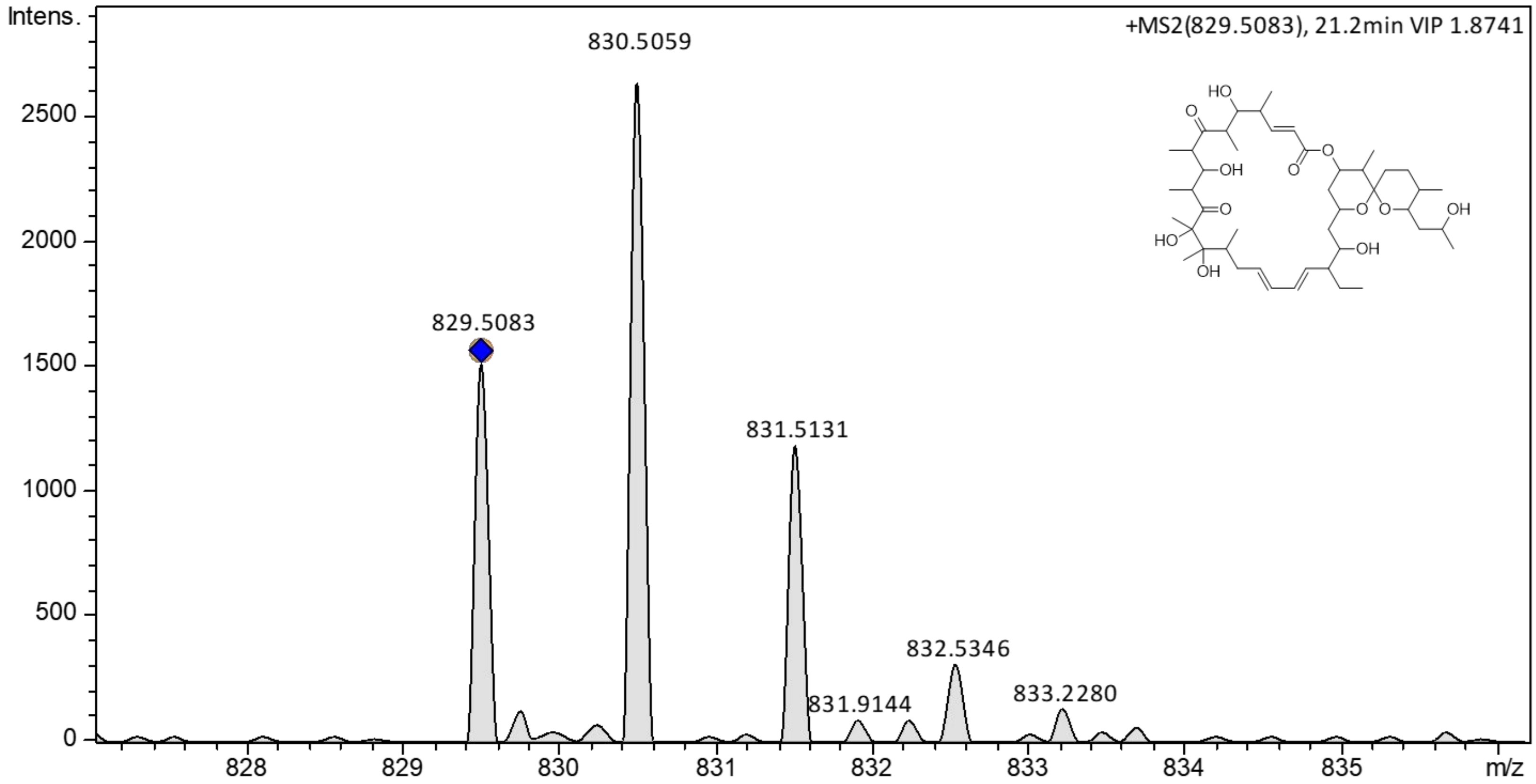

Figure SI 44. Mass spectrum of metabolite **44**.

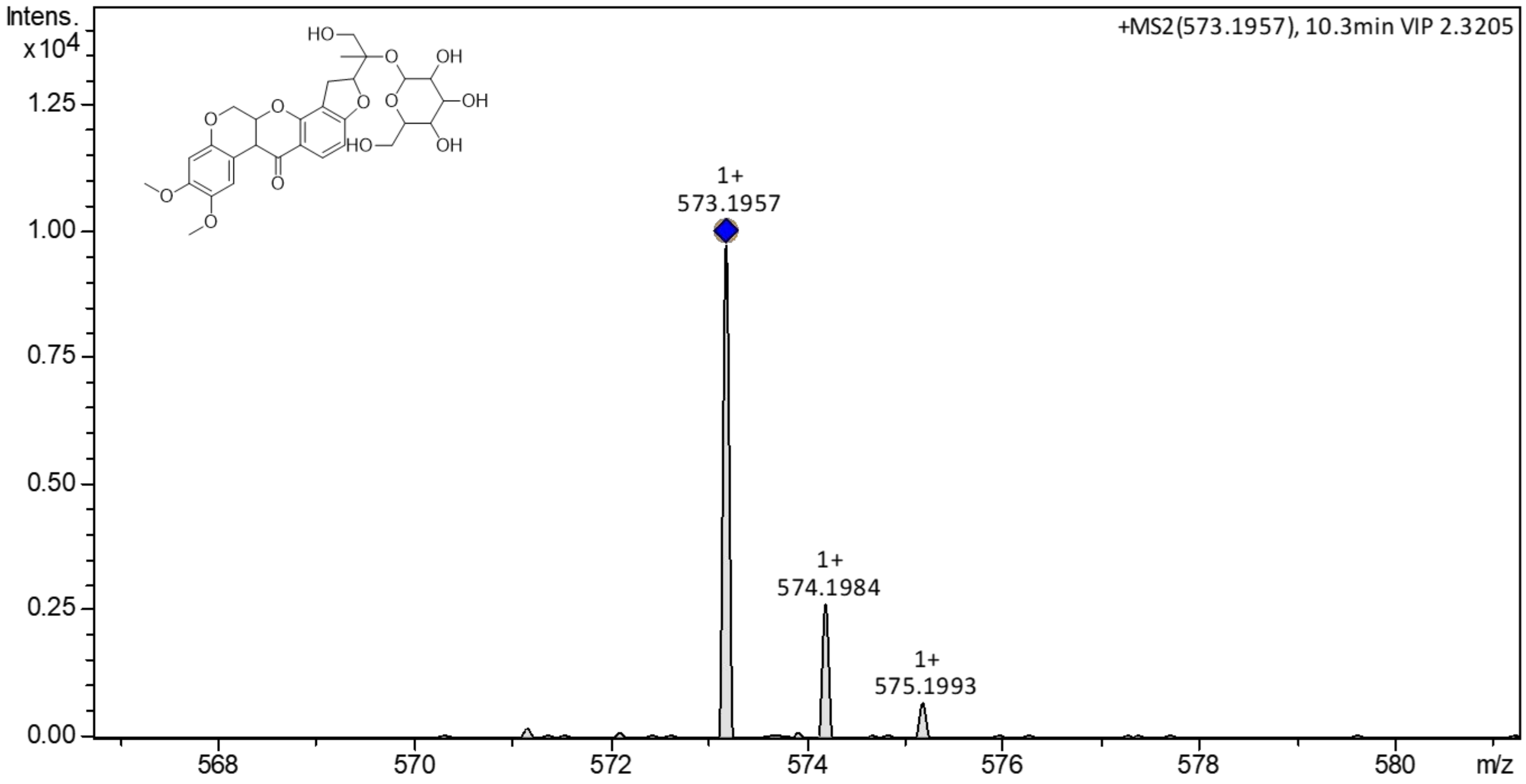

Figure SI 45. Mass spectrum of metabolite **45**.

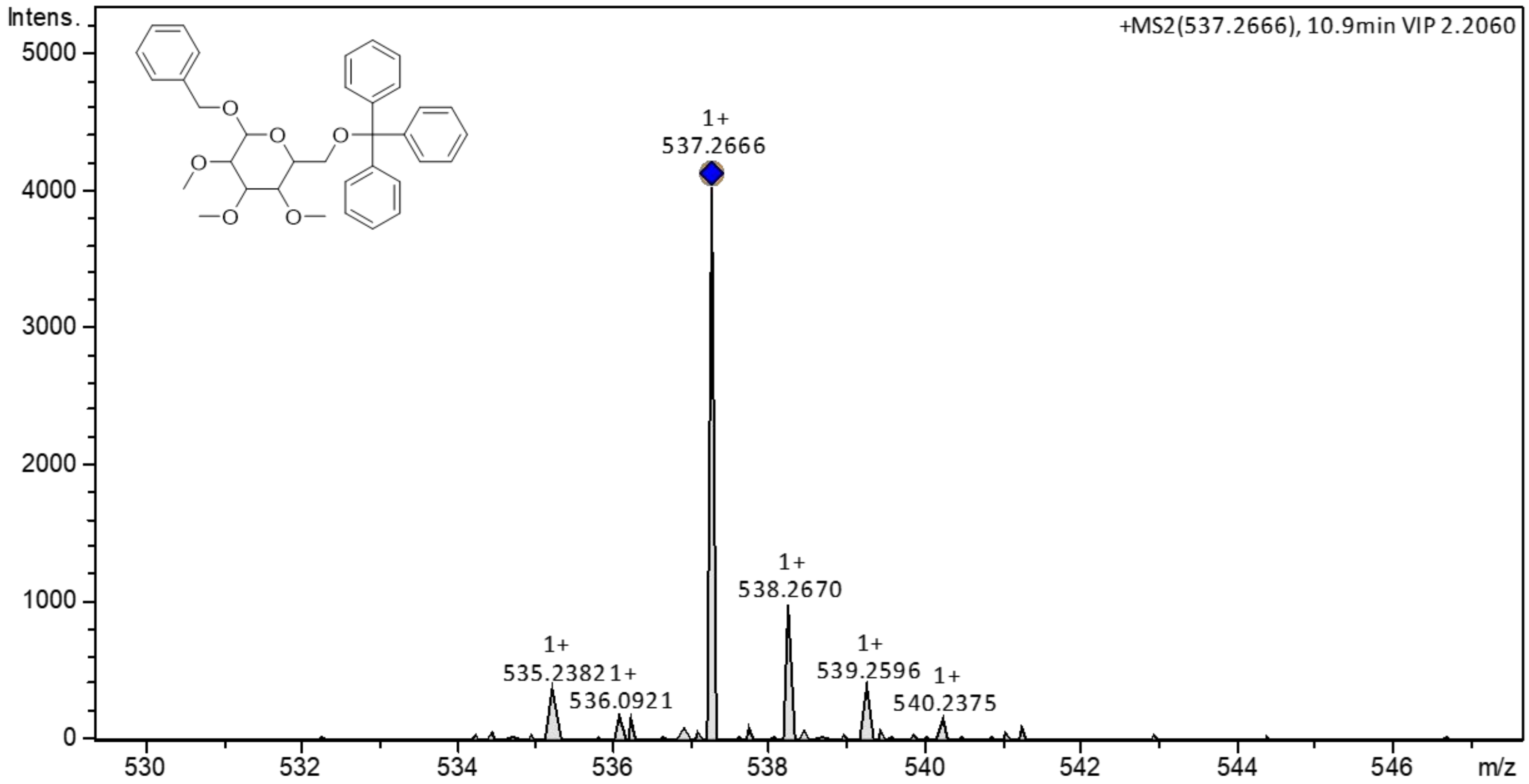

Figure SI 46. Mass spectrum of metabolite **46**.

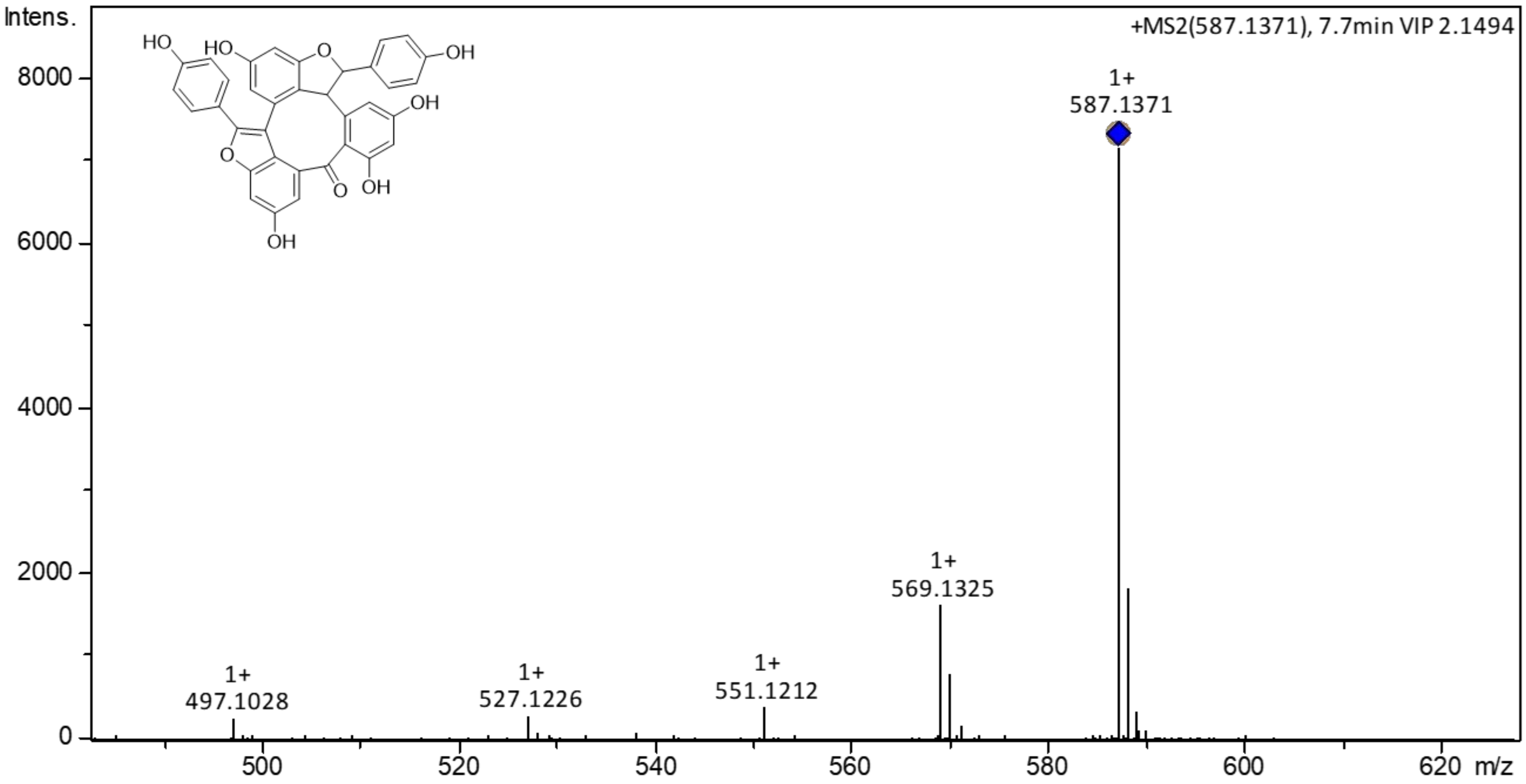

Figure SI 47. Mass spectrum of metabolite **47**.

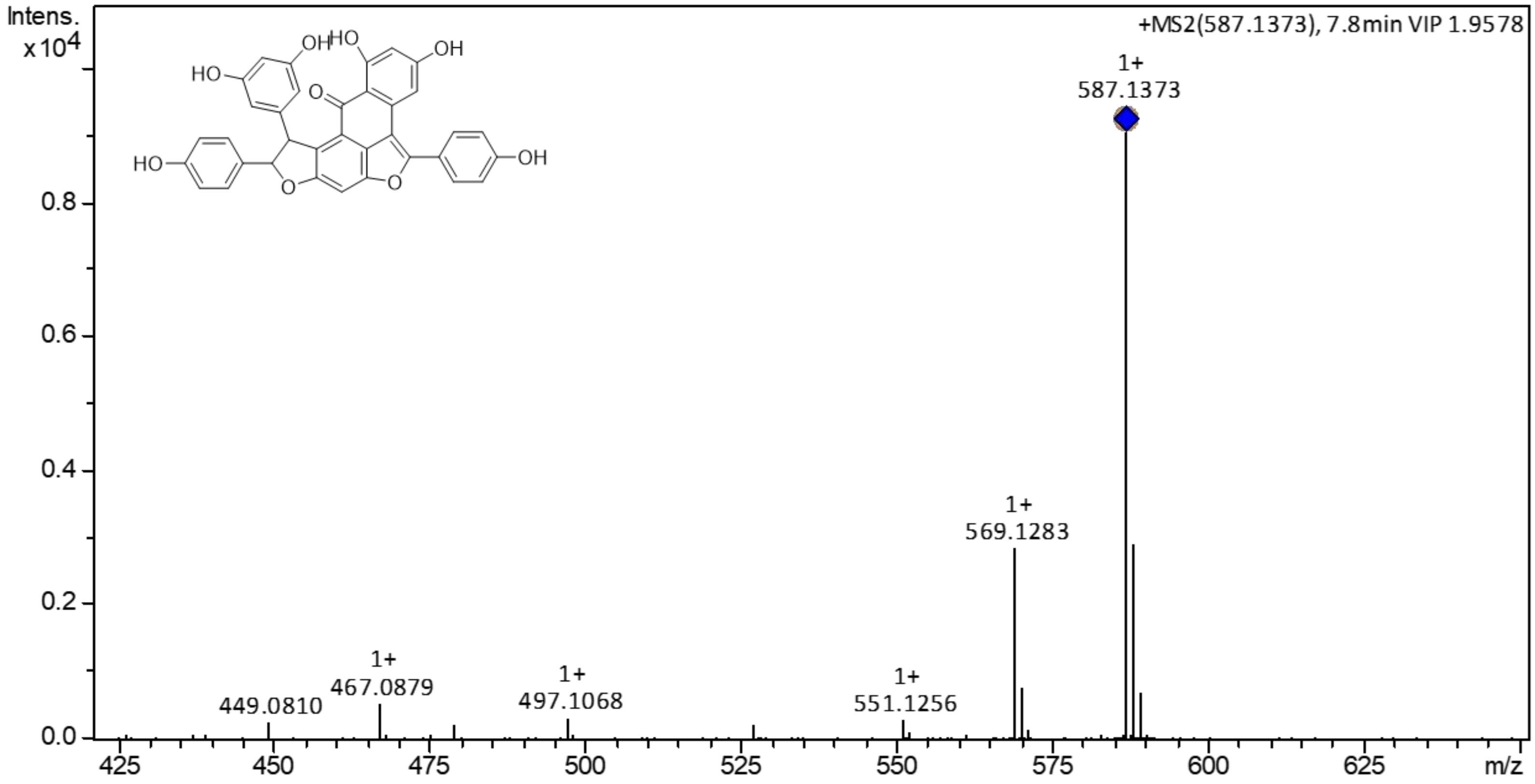

Figure SI 48. Mass spectrum of metabolite **48**.

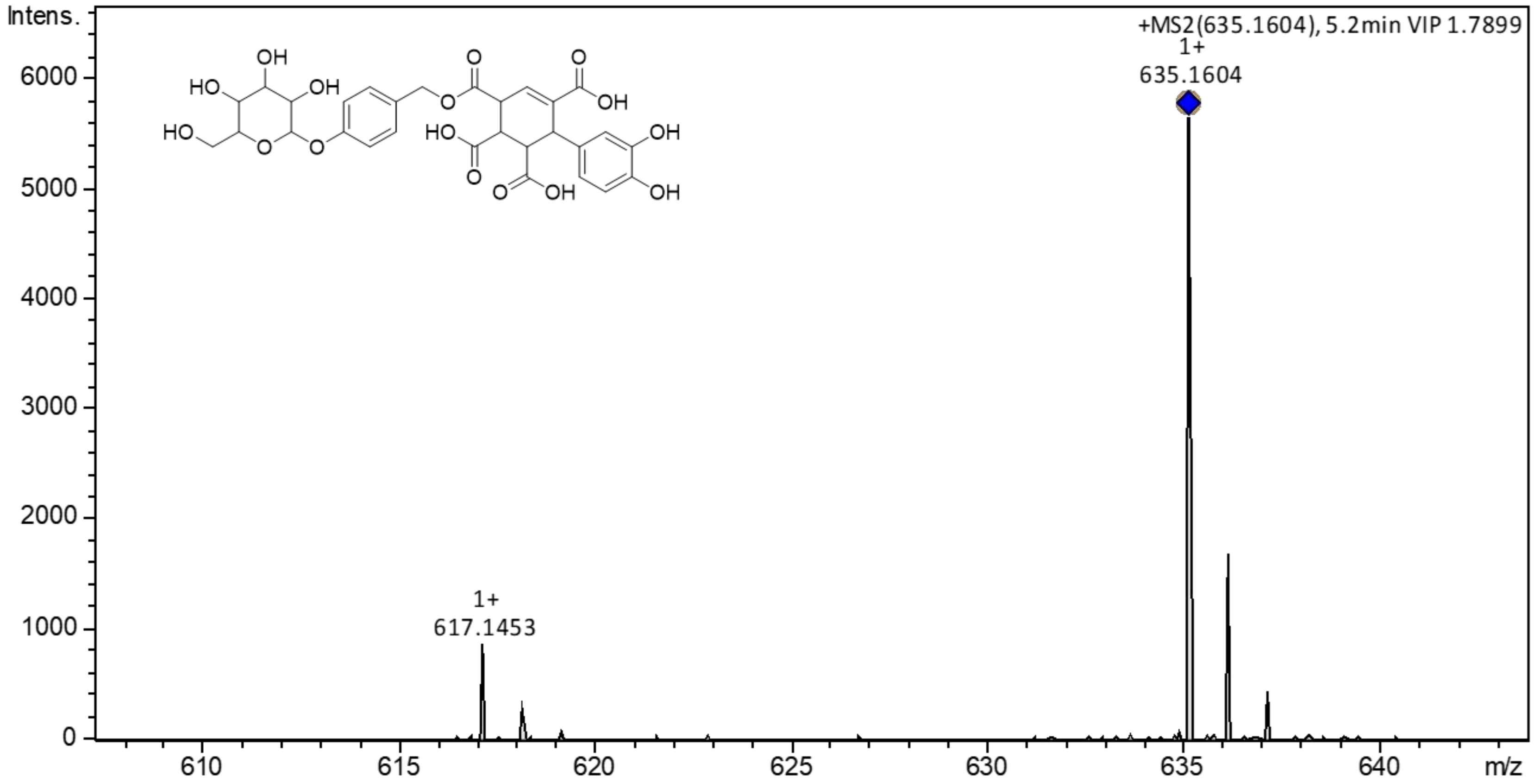

Figure SI 49. Mass spectrum of metabolite **49**.

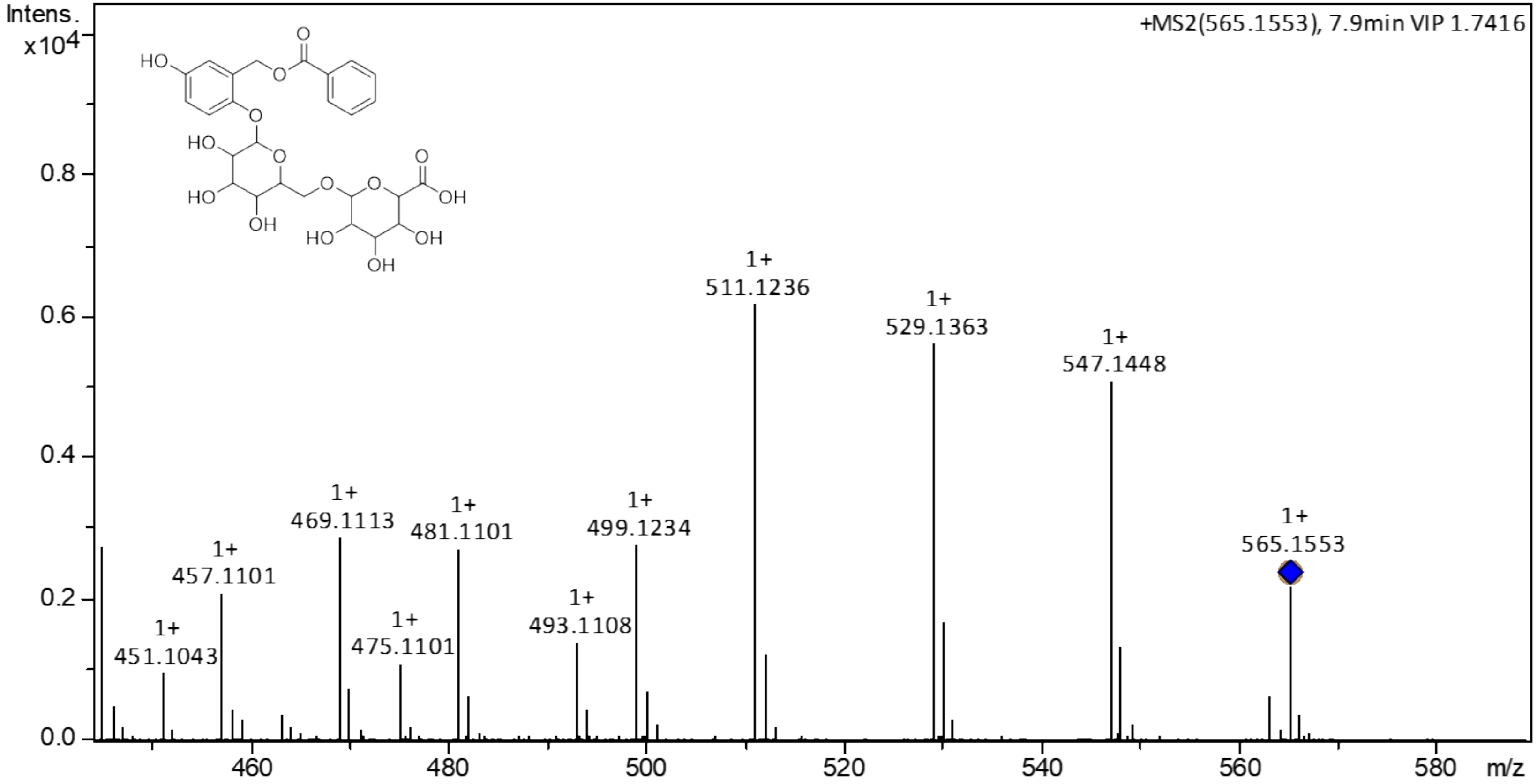

Figure SI 50. Mass spectrum of metabolite **50**.

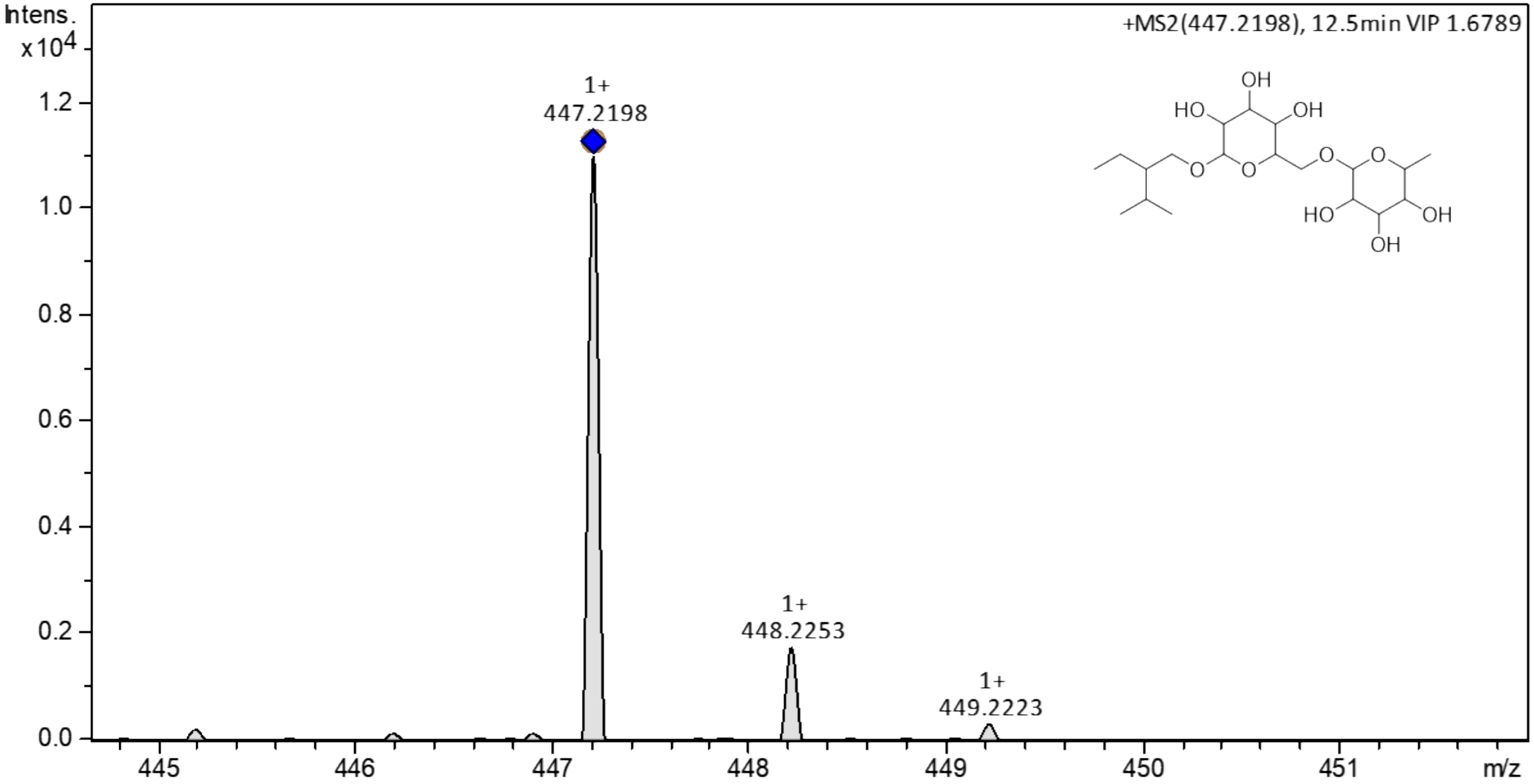

Figure SI 51. Mass spectrum of metabolite **51**.

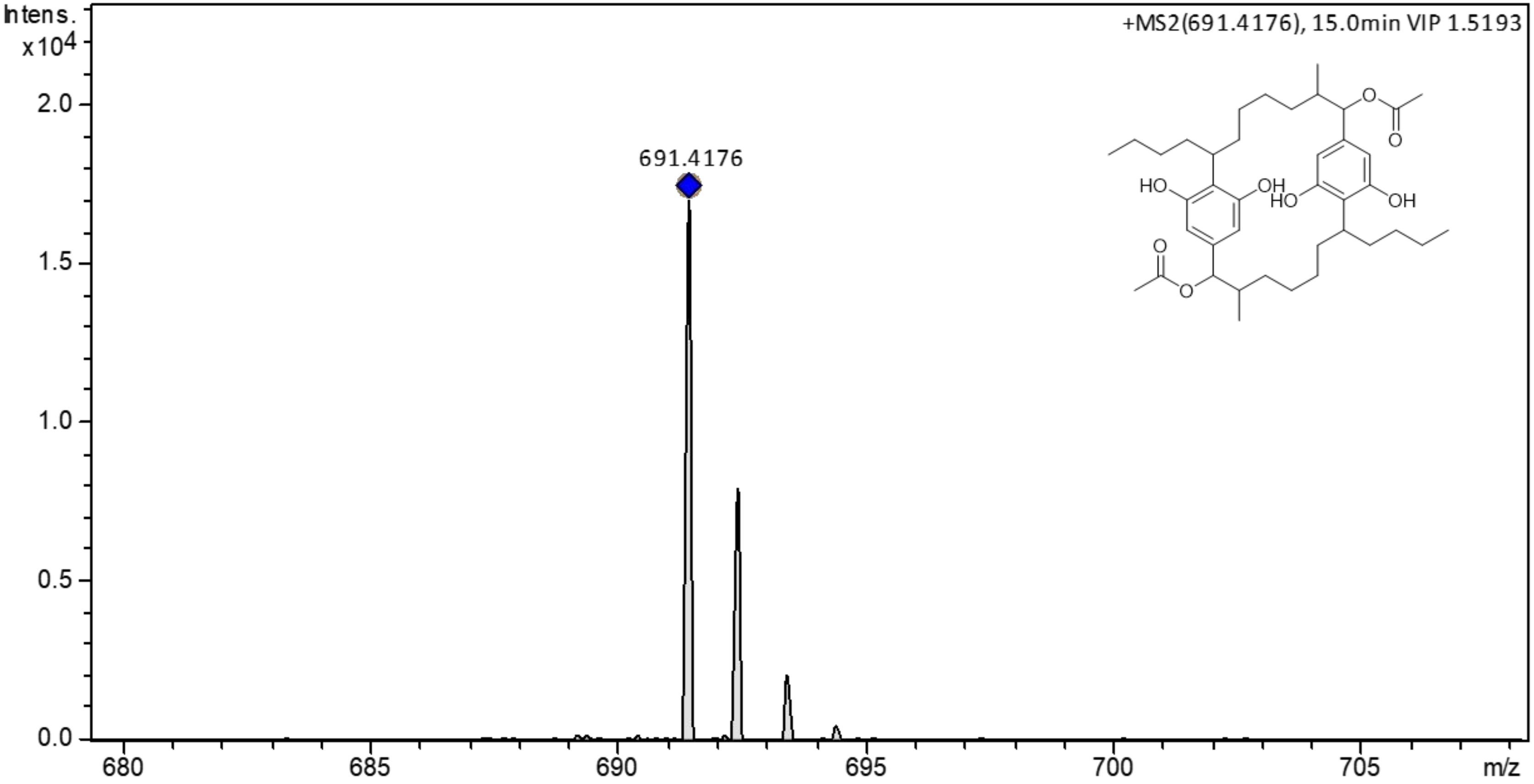

Figure SI 52. Mass spectrum of metabolite **52**.

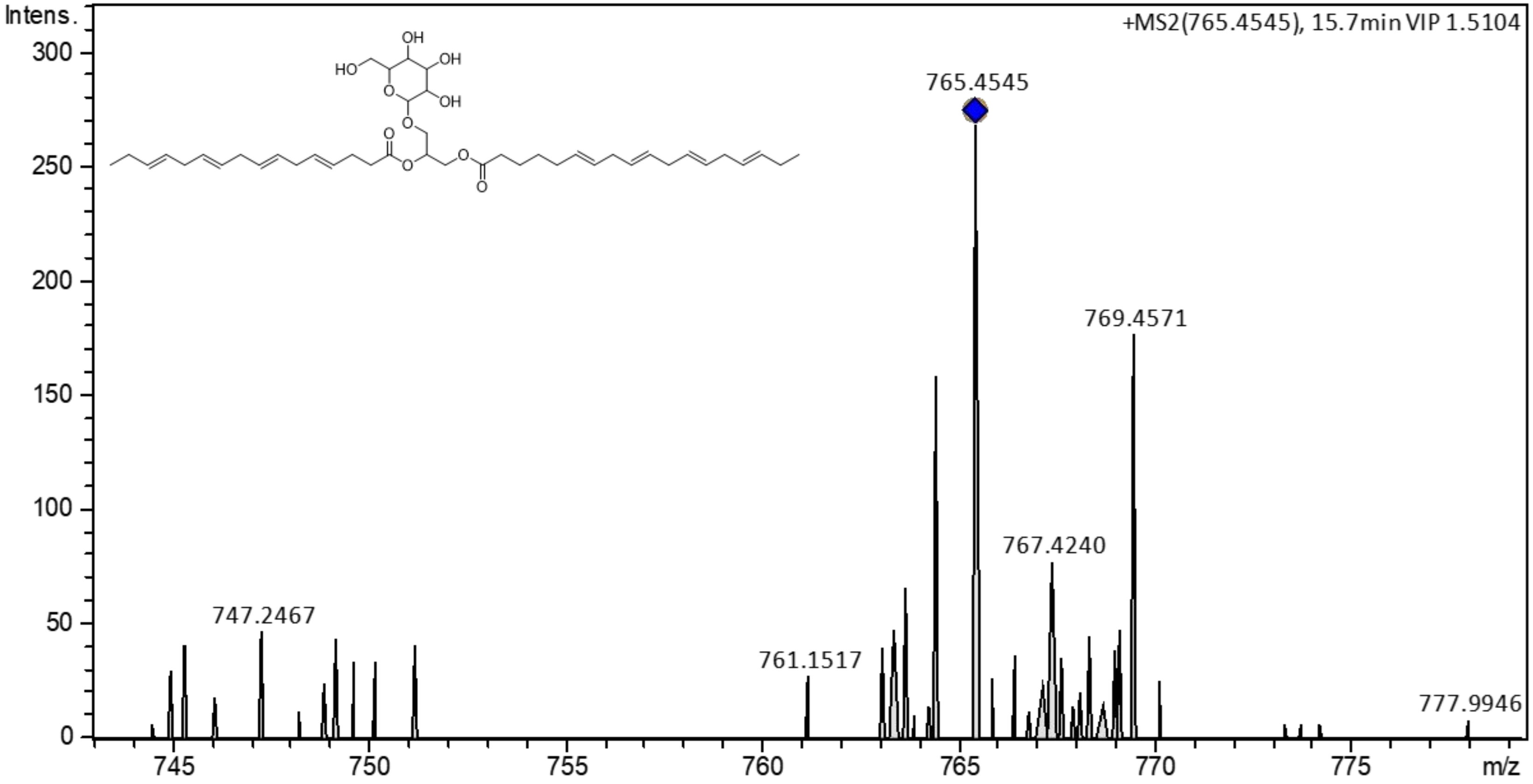

Figure SI 53. Mass spectrum of metabolite **53**.

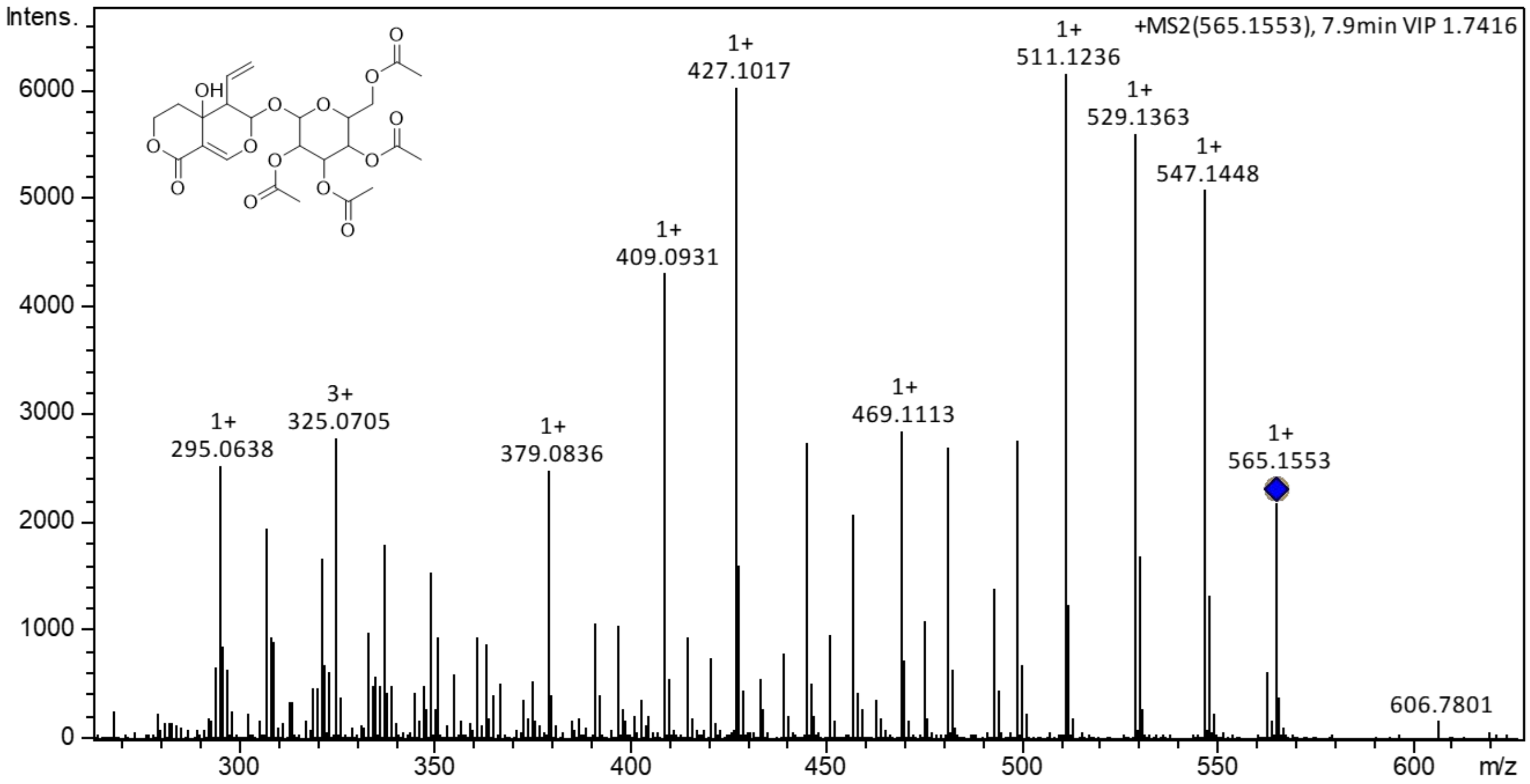

Figure SI 54. Mass spectrum of metabolite **54**.

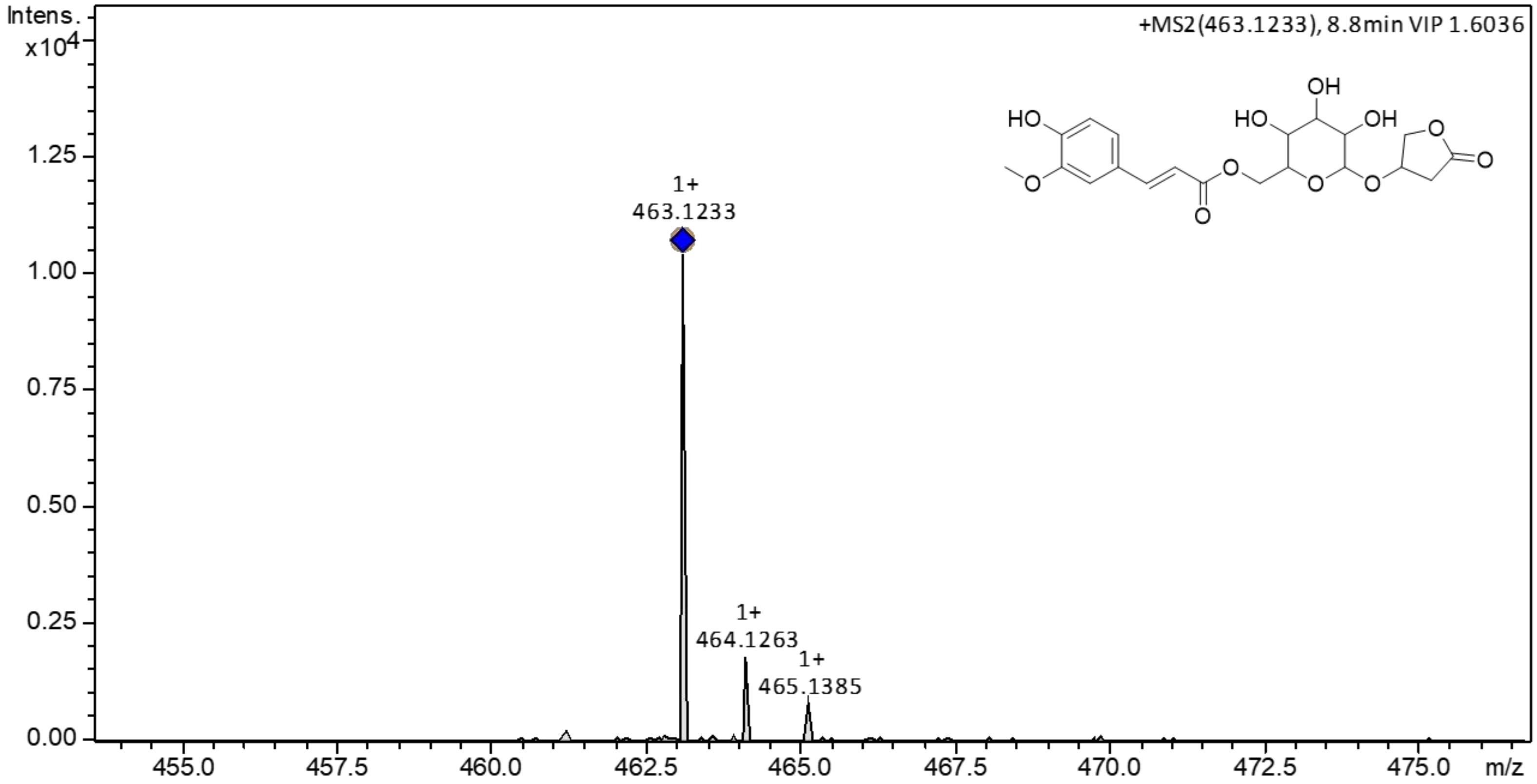

Figure SI 55. Mass spectrum of metabolite **55**.

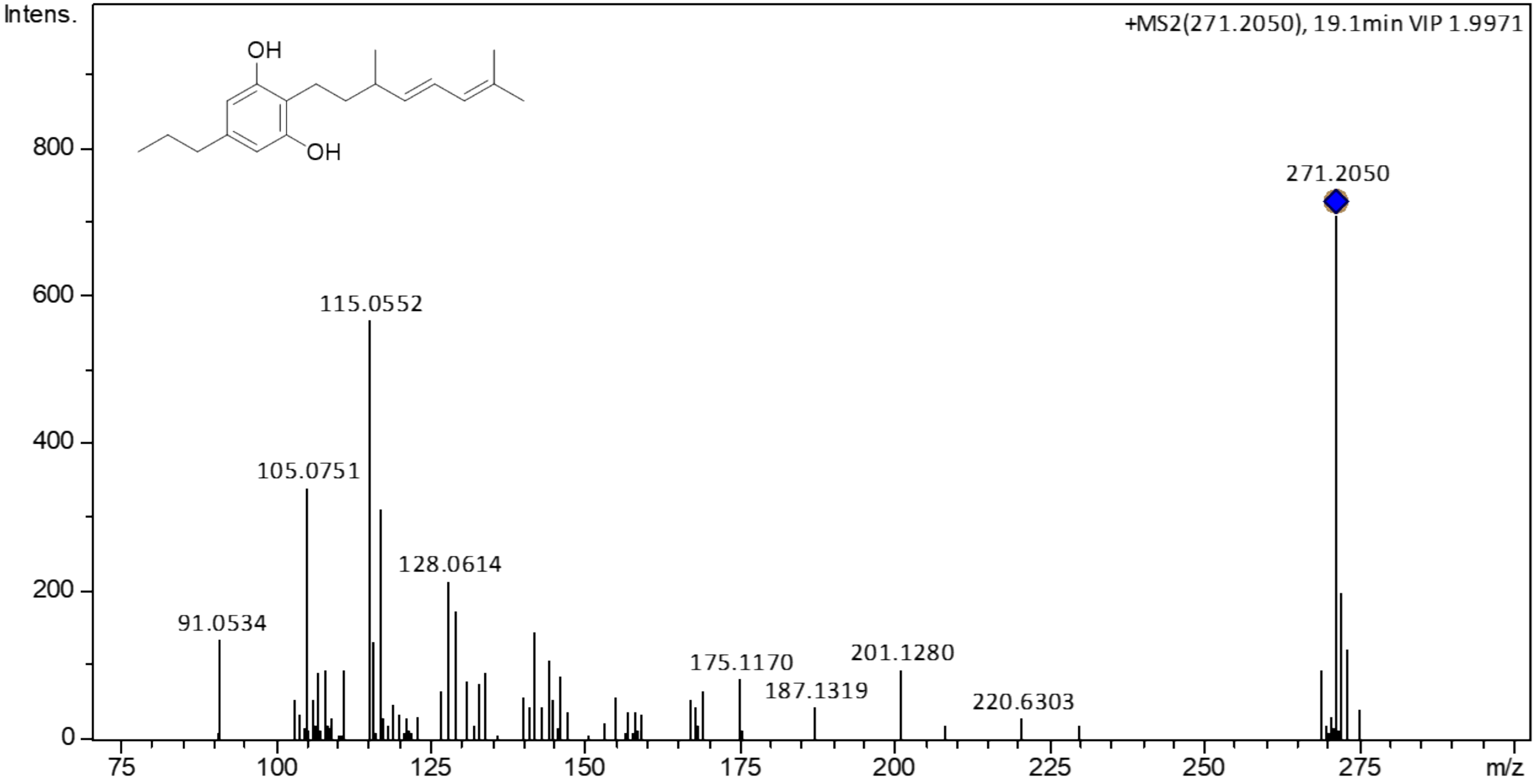

Figure SI 56. Mass spectrum of metabolite **56**.

Table S2. *Hyptis* species database.

Family	Species	Country of collection	Locality of collection	Voucher	Compound class	Chemical structure drawing	Compound name	Molecular formula	CAS number	Molecular mass
Lamiaceae	*H. albida*	Mexico	Huetamo	M-21850	Flavonoid	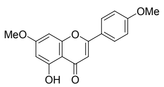	Apigenin-7,4-dimethyl ether	$C_{17}H_{14}O_5$	5128-44-9	298.0841
Lamiaceae	*H. albida*	Mexico	Huetamo	M-21850	Flavonoid	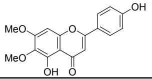	Cirsimaritin	$C_{17}H_{14}O_6$	6601-62-3	314.0790
Lamiaceae	*H. albida*	Mexico	Huetamo	M-21850	Flavonoid	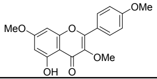	Kaempferol 3,7,4'-trimethyl ether	$C_{18}H_{16}O_6$	15486-34-7	328.0947
Lamiaceae	*H. albida*	Mexico	Huetamo	M-21850	Flavonoid	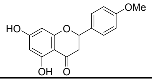	Isosakuranetin	$C_{16}H_{14}O_5$	480-43-3	286.0841
Lamiaceae	*H. albida*	Mexico	Huetamo	M-21850	Flavonoid	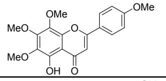	Gardenin B	$C_{19}H_{18}O_7$	2798-20-1	358.1053
Lamiaceae	*H. albida*	Mexico	Huetamo	M-21850	Flavonoid	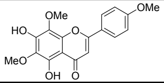	Nevadensin	$C_{18}H_{16}O_7$	10176-66-6	344.0896
Lamiaceae	*H. albida*	Mexico	Michoacan	8528-M	Triterpene	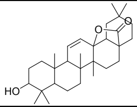	3ß-hydroxyolean-11-en-28,13ß-olide	$C_{30}H_{46}O_3$	N/A	454.3447
Lamiaceae	*H. albida*	Mexico	Michoacan	8528-M	Triterpene	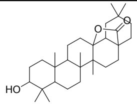	3ß-hydroxyolean-28,13ß-olide	$C_{30}H_{48}O_3$	N/A	456.3603
Lamiaceae	*H. albida*	Mexico	Michoacan	8528-M	Triterpene	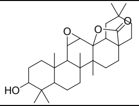	3ß-hydroxy-11,12a-epoxyolean-28,13ß-olide	$C_{30}H_{46}O_4$	N/A	470.3396
Lamiaceae	*H. albida*	Mexico	Michoacan	8528-M	Triterpene	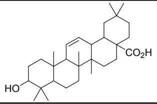	Oleanolic acid	$C_{30}H_{48}O_3$	508-02-1	456.3603

Lamiaceae	*H. atrorubens*	Haiti	Gros-Morne	10 473	Phenolic acid	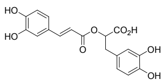	Rosmarinic acid	$C_{18}H_{16}O_8$	20283-92-5	360.0845
Lamiaceae	*H. atrorubens*	Haiti	Gros-Morne	10 473	Phenolic ester	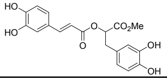	Methyl rosmarinate	$C_{19}H_{18}O_8$	99353-00-1	374.1002
Lamiaceae	*H. atrorubens*	Haiti	Gros-Morne	10 473	Flavonoid	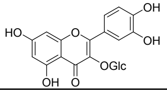	Hyperoside	$C_{21}H_{20}O_{12}$	482-36-0	464.0955
Lamiaceae	*H. brachiata*	Panama	Coclé Province	391	Flavonoid	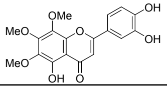	Sideritoflavone	$C_{18}H_{16}O_8$	70360-12-2	360.0845
Lamiaceae	*H. capitata*	Equador	Province of Esmeraldas	nr.012	Lignan	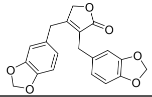	2,3-di(3′,4′-methylenedioxybenzyl)-2-buten-4-olide	$C_{20}H_{16}O_6$	137809-97-3	352.0947
Lamiaceae	*H. capitata*	Equador	Province of Esmeraldas	nr.012	α-Pyrone	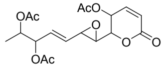	10-epi-olguine	$C_{18}H_{22}O_9$	137893-86-8	382.1264
Lamiaceae	*H. capitata*	Taiwan	Shan-De-Mun	N/A	Triterpene	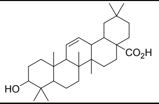	Oleanolic acid	$C_{30}H_{48}O_3$	508-02-1	456.3603
Lamiaceae	*H. capitata*	Equador	Province of Esmeraldas	nr.012	Lignan	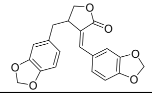	Savinin	$C_{20}H_{16}O_6$	493-95-8	352.0947
Lamiaceae	*H. capitata*	Equador	Province of Esmeraldas	nr.012	Lignan	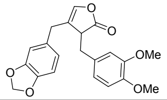	Guayadequiene	$C_{21}H_{20}O_6$	129502-61-0	368.1260
Lamiaceae	*H. capitata*	Taiwan	Shan-De-Mun	N/A	Triterpene	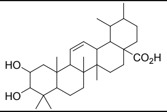	2alpha-hydroxyursolic Acid	$C_{30}H_{48}O_4$	4547-24-4	472.3553
Lamiaceae	*H. capitata*	Taiwan	Shan-De-Mun	N/A	Triterpene	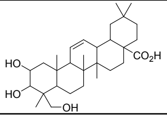	Hyptatic acid A	$C_{30}H_{48}O_5$	118711-55-0	488.3502

Lamiaceae	*H. capitata*	Taiwan	Shan-De-Mun	N/A	Triterpene	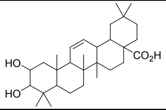	Maslinic acid	$C_{30}H_{48}O_4$	4373-41-5	472.3553
Lamiaceae	*H. capitata*	Taiwan	Shan-De-Mun	N/A	Triterpene	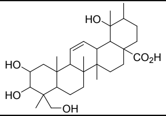	Hyptatic acid B	$C_{30}H_{48}O_6$	105706-08-9	504.3451
Lamiaceae	*H. comaroides*	Brazil	Rio Grande do Sul	UFSM 15406	Phenolic acid	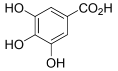	Gallic acid	$C_7H_6O_5$	149-91-7	170.0215
Lamiaceae	*H. comaroides*	Brazil	Rio Grande do Sul	UFSM 15406	Phenolic acid	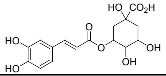	Chlorogenic acid	$C_{16}H_{18}O_9$	327-97-9	354.0951
Lamiaceae	*H. comaroides*	Brazil	Rio Grande do Sul	UFSM 15406	Phenolic acid	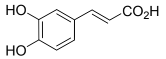	Caffeic acid	$C_9H_8O_4$	331-39-5	180.0423
Lamiaceae	*H. comaroides*	Brazil	Rio Grande do Sul	UFSM 15406	Phenolic acid	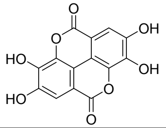	Ellagic acid	$C_{14}H_6O_8$	476-66-4	302.0063
Lamiaceae	*H. comaroides*	Brazil	Rio Grande do Sul	UFSM 15406	Flavonoid	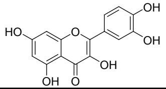	Quercetin	$C_{15}H_{10}O_7$	117-39-5	302.0427
Lamiaceae	*H. comaroides*	Brazil	Rio Grande do Sul	UFSM 15406	Flavonoid	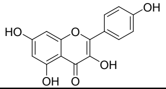	Kaempferol	$C_{15}H_{10}O_6$	520-18-3	286.0477
Lamiaceae	*H. comaroides*	Brazil	Rio Grande do Sul	SMDB 12333	Abietane diterpene	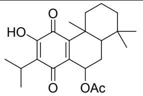	7α-acetoxyroyleanone	$C_{22}H_{30}O_5$	6812-88-0	374.2093
Lamiaceae	*H. comaroides*	Brazil	Rio Grande do Sul	SMDB 12333	Abietane diterpene	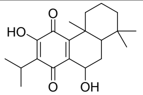	Horminone	$C_{20}H_{28}O_4$	21887-01-4	332.1988
Lamiaceae	*H. comaroides*	Brazil	Rio Grande do Sul	SMDB 12333	Abietane diterpene	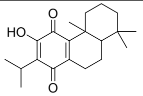	Royleanone	$C_{20}H_{28}O_3$	6812-87-9	316.2038
Lamiaceae	*H. comaroides*	Brazil	Rio Grande do Sul	SMDB 12333	Abietane diterpene	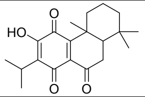	7-ketoroyleanone	$C_{20}H_{26}O_4$	57685-32-2	330.1831

Lamiaceae	*H. comaroides*	Brazil	Rio Grande do Sul	SMDB 12333	Abietane diterpene	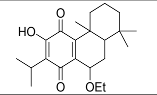	7α-ethoxyroyleanone	$C_{22}H_{32}O_4$	101409-68-1	360.2301
Lamiaceae	*H. comaroides*	Brazil	Rio Grande do Sul	SMDB 12333	Abietane diterpene	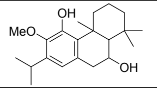	Iguestol	$C_{21}H_{32}O_3$	860018-57-1	332.2351
Lamiaceae	*H. comaroides*	Brazil	Rio Grande do Sul	SMDB 12333	Abietane diterpene	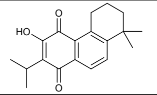	Deoxyneocryptotanshi none	$C_{19}H_{22}O_3$	27468-20-8	298.1569
Lamiaceae	*H. comaroides*	Brazil	Rio Grande do Sul	SMDB 12333	Abietane diterpene	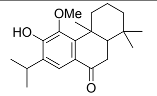	12-hydroxy-11-metoxyabieta-8,11,13-trien-7-one	$C_{21}H_{30}O_3$	N/A	330.2195
Lamiaceae	*H. comaroides*	Brazil	Rio Grande do Sul	SMDB 12333	Abietane diterpene	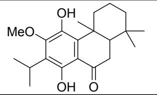	Inuroyleanol	$C_{21}H_{30}O_4$	57685-31-1	346.2144
Lamiaceae	*H. comaroides*	Brazil	Rio Grande do Sul	SMDB 12333	Abietane diterpene	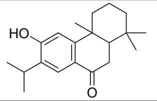	Sugiol	$C_{20}H_{28}O_2$	511-05-7	300.2089
Lamiaceae	*H. comaroides*	Brazil	Rio Grande do Sul	SMDB 12333	Abietane diterpene	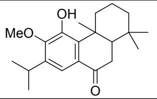	Cryptojaponol	$C_{21}H_{30}O_3$	16755-52-5	330.2195
Lamiaceae	*H. comaroides*	Brazil	Rio Grande do Sul	SMDB 12333	Abietane diterpene	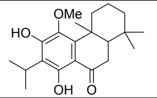	Orthosiphonol	$C_{21}H_{30}O_4$	432491-41-3	346.2144
Lamiaceae	*H. crassifolia*	Brazil	Bahia	UFB No. 95.183	Abietane diterpene	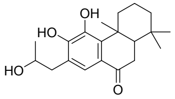	11,12,16-trihydroxy-17(15→16)-abeo-abieta-8,11,13-trien-7-one	$C_{20}H_{28}O_4$	1888419-65-5	332.1988
Lamiaceae	*H. crassifolia*	Brazil	Bahia	UFB No. 95.183	Abietane diterpene	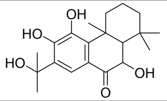	6α,11,12,15-tetrahydroxy-8,11,13-abietatrien-7-one	$C_{20}H_{28}O_5$	1912463-24-1	348.1937
Lamiaceae	*H. crassifolia*	Brazil	Bahia	UFB No. 95.183	Abietane diterpene	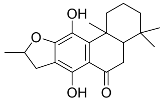	(16S)-12,16-epoxy-11,14-dihydroxy-17(15→16)-abeo-abieta-8,11,13-trien-7-one	$C_{20}H_{26}O_4$	160927-83-3	330.1831

Lamiaceae	*H. crassifolia*	Brazil	Bahia	UFB No. 95.183	Abietane diterpene	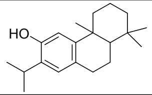	Ferruginol	$C_{20}H_{30}O$	514-62-5	286.2297
Lamiaceae	*H. crassifolia*	Brazil	Bahia	UFB No. 95.183	Abietane diterpene	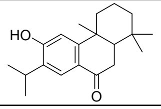	Sugiol	$C_{20}H_{28}O_2$	511-05-7	300.2089
Lamiaceae	*H. crassifolia*	Brazil	Bahia	UFB No. 95.183	Diterpene	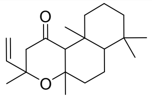	11-Oxomanoyl oxide	$C_{20}H_{32}O_2$	61242-47-5	304.2402
Lamiaceae	*H. crassifolia*	Brazil	Bahia	UFB No. 95.183	Abietane diterpene	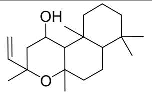	11β-hydroxymanoyloxide	$C_{20}H_{34}O_2$	56624-70-5	306.2559
Lamiaceae	*H. dilatata*	Panama	Santiago de Veraguas	N/A	Abietane diterpene	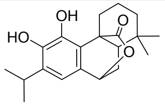	Carnosol	$C_{20}H_{26}O_4$	5957-80-2	330.1831
Lamiaceae	*H. dilatata*	Panama	Santiago de Veraguas	N/A	Abietane diterpene	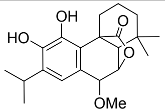	Epimethylrosmanol	$C_{25}H_{32}O_7$	212703-95-2	444.2148
Lamiaceae	*H. oblongifolia*	Mexico	N/A	N/A	α-Pyrone	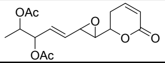	4-deacetoxy-10-epi-olguine	$C_{16}H_{20}O_7$	N/A	324.2093
Lamiaceae	*H. dilatata*	Panama	Santiago de Veraguas	N/A	Abietane diterpene	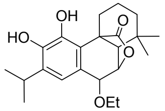	Epiethylrosmanol	$C_{26}H_{34}O_7$	212703-46-3	458.2305
Lamiaceae	*H. dilatata*	Panama	Santiago de Veraguas	N/A	Abietane diterpene	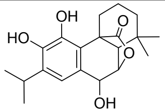	Epirosmanol	$C_{20}H_{26}O_5$	93380-12-2	346.1780
Lamiaceae	*H. dilatata*	Panama	Santiago de Veraguas	N/A	Abietane diterpene	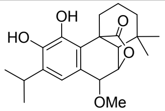	Methylrosmanol	$C_{21}H_{28}O_5$	113085-62-4	360.1937
Lamiaceae	*H. dilatata*	Panama	Santiago de Veraguas	N/A	Abietane diterpene	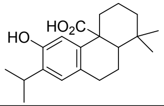	Pisiferic acid	$C_{23}H_{32}O_4$	67494-15-9	372.2301

Lamiaceae	*H. dilatata*	Panama	Santiago de Veraguas	N/A	Abietane diterpene	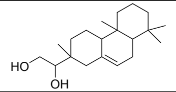	Esquirolin B	$C_{23}H_{36}O_4$	136236-45-8	376.2614
Lamiaceae	*H. dilatata*	Panama	Santiago de Veraguas	N/A	Abietane diterpene	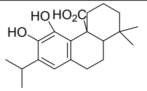	Carnosic acid	$C_{21}H_{28}O_3$	3650-09-7	328.2038
Lamiaceae	*H. dilatata*	Panama	Santiago de Veraguas	N/A	Abietane diterpene	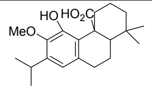	12-O-methylcarnosic acid	$C_{25}H_{34}O_6$	62201-71-2	430.5410
Lamiaceae	*H. dilatata*	Panama	Santiago de Veraguas	N/A	Abietane diterpene	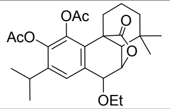	Diacetylepiethylrosma nol	$C_{26}H_{34}O_7$	N/A	458.2305
Lamiaceae	*H. emoryi*	N/A	N/A	N/A	Triterpene	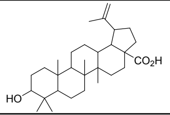	Betulinic acid	$C_{30}H_{48}O_3$	472-15-1	456.3603
Lamiaceae	*H. fasciculata*	N/A	N/A	N/A	Triterpene	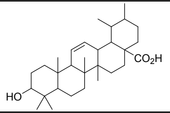	Ursolic acid	$C_{30}H_{48}O_3$	77-52-1	456.3603
Lamiaceae	*H. fasciculata*	Brazil	São Paulo	B2-154	Flavonoid	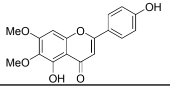	Cirsimaritin	$C_{17}H_{14}O_6$	6601-62-3	314.0790
Lamiaceae	*H. fasciculata*	Brazil	São Paulo	B2-154	Flavonoid	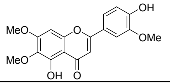	Cirsilineol	$C_{18}H_{16}O_7$	41365-32-6	344.0896
Lamiaceae	*H. fasciculata*	Brazil	São Paulo	B2-155	Labdane diterpene	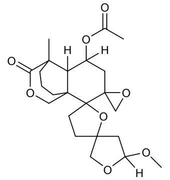	15β-methoxyfaciculatin	$C_{23}H_{32}O_8$	876407-56-6	436.2097
Lamiaceae	*H. fasciculata*	Brazil	Rio Grande do Sul	UFRGS-537	Flavonoid	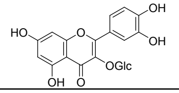	Isoquercitrin	$C_{21}H_{20}O_{12}$	482-35-9	464.0955
Lamiaceae	*H. fasciculata*	Brazil	Pernambuco	N/A	Flavonoid	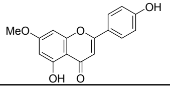	Genkwanin	$C_{16}H_{12}O_5$	437-64-9	284.0685

Lamiaceae	*H. incana*	Brazil	São Paulo	N/A	Sesquiterpene	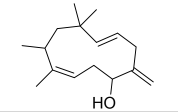	Hyptishumulene I	$C_{15}H_{24}O_2$	1820926-17-7	236.1776
Lamiaceae	*H. incana*	Brazil	São Paulo	N/A	Monoterpene	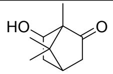	6-hydroxycamphor	$C_{10}H_{16}O_2$	29050-85-9	168.1150
Lamiaceae	*H. incana*	Brazil	São Paulo	N/A	Abietane diterpene	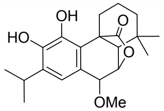	7-methoxyrosmanol	$C_{21}H_{28}O_5$	113085-62-4	360.1937
Lamiaceae	*H. incana*	Brazil	São Paulo	N/A	Abietane diterpene	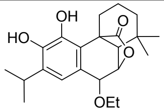	7-ethoxyrosmanol	$C_{22}H_{30}O_5$	111200-01-2	374.2093
Lamiaceae	*H. incana*	Brazil	São Paulo	N/A	Abietane diterpene	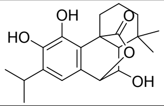	Isorosmanol	$C_{20}H_{26}O_5$	93780-80-4	346.1780
Lamiaceae	*H. incana*	Brazil	São Paulo	N/A	Diterpene	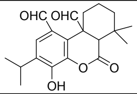	Safficinolide	$C_{20}H_{24}O_5$	153660-18-5	344.1624
Lamiaceae	*H. lacustris*	Brazil	São Paulo	Silva-Luz 296	Phenolic acid	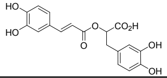	Rosmarinic acid	$C_{18}H_{16}O_8$	20283-92-5	360.0845
Lamiaceae	*H. lacustris*	Brazil	São Paulo	Silva-Luz 296	Abietane diterpene	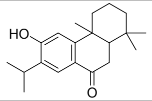	Sugiol	$C_{20}H_{28}O_2$	511-05-7	300.2089
Lamiaceae	*H. lacustris*	Brazil	São Paulo	Silva-Luz 296	Abietane diterpene	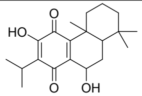	Horminone	$C_{20}H_{28}O_4$	21887-01-4	332.1988
Lamiaceae	*H. lacustris*	Brazil	São Paulo	Silva-Luz 296	Abietane diterpene	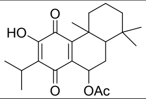	7-acetoxyhorminone	$C_{22}H_{30}O_5$	6812-88-0	374.2093
Lamiaceae	*H. lacustris*	Brazil	São Paulo	Silva-Luz 296	Abietane diterpene	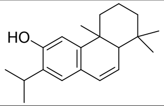	6,7-dehydroferruginol	$C_{20}H_{28}O$	34539-84-9	284.2140

Lamiaceae	*H. lacustris*	Brazil	São Paulo	Silva-Luz 296	Abietane diterpene	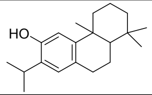	Ferruginol	$C_{20}H_{30}O$	514-62-5	286.2297
Lamiaceae	*H. lacustris*	Brazil	São Paulo	Silva-Luz 296	Abietane diterpene	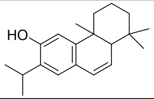	6,7-Dehydroroyleanone	$C_{20}H_{26}O_3$	6855-99-8	314.1882
Lamiaceae	*H. lacustris*	Brazil	São Paulo	Silva-Luz 296	Abietane diterpene	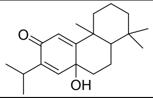	8β-hydroxy-9(11),13-abietadien-12-one	$C_{20}H_{30}O_2$	62716-38-5	302.2246
Lamiaceae	*H. lacustris*	Brazil	São Paulo	Silva-Luz 296	Abietane diterpene	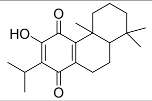	Royleanone	$C_{20}H_{28}O_3$	6812-87-9	316.2038
Lamiaceae	*H. lacustris*	Brazil	São Paulo	Silva-Luz 296	Steroid	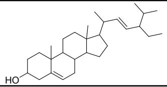	Stigmasterol	$C_{29}H_{48}O$	83-48-7	412.3705
Lamiaceae	*H. lacustris*	Brazil	São Paulo	Silva-Luz 296	Steroid	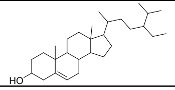	Sitosterol	$C_{29}H_{50}O$	83-46-5	414.3862
Lamiaceae	*H. lanceolata*	Nigeria	Abia	N/A	Alkaloid	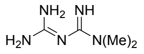	Imidodicarbonimidic diamide, N,N-dimethyl	$C_2H_7N_5$	56-03-1	129.1014
Lamiaceae	*H. lanceolata*	Nigeria	Abia	N/A	Fatty acid	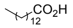	Tetradecanoic acid	$C_{14}H_{28}O_2$	544-63-8	228.2089
Lamiaceae	*H. lanceolata*	Nigeria	Abia	N/A	Hydrocarbon	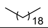	Eicosyne	$C_{20}H_{38}$	451500-40-6	278.2974
Lamiaceae	*H. lanceolata*	Nigeria	Abia	N/A	Fatty acid ester	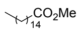	Hexadecanoic acid, methyl ester	$C_{17}H_{34}O_2$	112-39-0	270.2656
Lamiaceae	*H. lanceolata*	Nigeria	Abia	N/A	Fatty acid ester	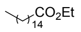	Hexadecanoic acid ethyl ester	$C_{18}H_{36}O_2$	628-97-7	284.2715
Lamiaceae	*H. lanceolata*	Nigeria	Abia	N/A	Fatty acid ester		9,12,15-Octadecatrienoic acid, ethyl ester	$C_{20}H_{40}O$	121198-16-1	296.3079
Lamiaceae	*H. lanceolata*	Nigeria	Abia	N/A	Fatty acid ester		9,12,15-Octadecatrienoic acid, methyl ester	$C_{20}H_{34}O_2$	29565-44-4	306.2559
Lamiaceae	*H. lanceolata*	Nigeria	Abia	N/A	Fatty acid	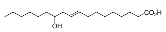	9-Octadecenoic acid, 12-hydroxy	$C_{18}H_{34}O_3$	2027-47-6	298.2508

Lamiaceae	*H. lanceolata*	Cameroon	N/A	24136/S RSCAM/ HNC.	Triterpene	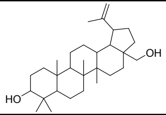	Betulin	$C_{30}H_{50}O_2$	473-98-3	442.3811
Lamiaceae	*H. lanceolata*	Cameroon	N/A	24136/S RSCAM/ HNC.	Triterpene	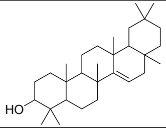	Taraxerol	$C_{30}H_{48}O_3$	127-22-0	456.3603
Lamiaceae	*H. lanceolata*	Cameroon	N/A	24136/S RSCAM/ HNC.	Triterpene	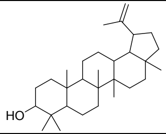	Lupeol	$C_{30}H_{50}O$	545-47-1	426.3862
Lamiaceae	*H. lanceolata*	Cameroon	N/A	24136/S RSCAM/ HNC.	Steroid	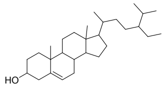	Sitosterol	$C_{29}H_{50}O$	83-46-5	414.3862
Lamiaceae	*H. lanceolata*	Cameroon	N/A	24136/S RSCAM/ HNC.	Steroid	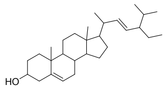	Stigmasterol	$C_{29}H_{48}O$	83-48-7	412.3705
Lamiaceae	*H. lanceolata*	Cameroon	N/A	24136/S RSCAM/ HNC.	Steroid	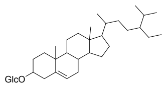	βsitosterol-3-O-β-D-glucopyranoside	$C_{35}H_{60}O_6$	474-58-8	576.4390
Lamiaceae	*H. lanceolata*	Cameroon	N/A	24136/S RSCAM/ HNC.	Fatty acid	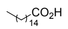	Palmitic Acid	$C_{16}H_{32}O_2$	57-10-3	256.2402
Lamiaceae	*H. lanceolata*	Cameroon	N/A	24136/S RSCAM/ HNC.	Alcohol	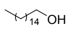	Hexacosanol	$C_{26}H_{54}O$	506-52-5	382.4175
Lamiaceae	*H. macrostachys*	Brazil	Paraiba	N/A	Pyrone	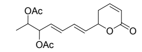	Hyptenolide	$C_{16}H_{22}O_6$	1638733-30-8	310.1416
Lamiaceae	*H. marrubioides*	Brazil	Minas Gerais	N/A	Phenolic compound	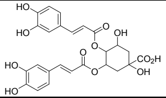	3,4-O-(Z)-dicaffeoylquinic acid	$C_{25}H_{24}O_{12}$	14534-61-3	516.1268
Lamiaceae	*H. marrubioides*	Brazil	Minas Gerais	N/A	Flavonoid	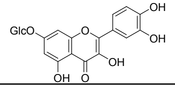	Quercetin-7-O-glucoside	$C_{21}H_{20}O_{12}$	491-50-9	464.0955

Lamiaceae	*H. marrubioides*	Brazil	Minas Gerais	N/A	Sesquiterpene	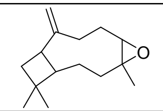	Caryophyllene oxide	$C_{15}H_{24}O$	1139-30-6	220.1827
Lamiaceae	*H. marrubioides*	Brazil	Minas Gerais	N/A	Sesquiterpene	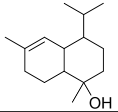	τ-cadinol	$C_{15}H_{26}O$	5937-11-1	222.9840
Lamiaceae	*H. marrubioides*	Brazil	Minas Gerais	N/A	Fatty acid ester	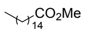	Methyl hexadecanoate	$C_{17}H_{34}O_2$	112-39-0	240.2559
Lamiaceae	*H. marrubioides*	Brazil	Minas Gerais	N/A	Fatty acid ester		9,12,15-Octadecatrienoic acid, methyl ester	$C_{19}H_{32}O_2$	7361-80-0	292.2402
Lamiaceae	*H. marrubioides*	Brazil	Minas Gerais	N/A	Phenolic compound	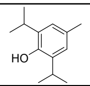	2,6-Diisopropyl-4-methylphenol	$C_{13}H_{20}O$	20766-99-8	192.1514
Lamiaceae	*H. marrubioides*	Brazil	Minas Gerais	N/A	Triterpene	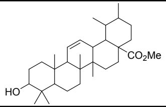	Ursolic acid methyl ester	$C_{31}H_{50}O_3$	32208-45-0	470.3760
Lamiaceae	*H. marrubioides*	Brazil	Minas Gerais	N/A	Fatty acid ester	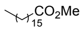	Methyl heptadecanoate	$C_{18}H_{36}O_2$	1731-92-6	284.2715
Lamiaceae	*H. marrubioides*	Brazil	Minas Gerais	N/A	Hydrocarbon	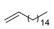	Heptadec-1-ene	$C_{17}H_{34}$	6765-39-5	238.2661
Lamiaceae	*H. marrubioides*	Brazil	Minas Gerais	N/A	Diterpene	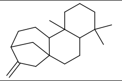	Kaur-16-ene	$C_{20}H_{32}$	562-28-7	272.2504
Lamiaceae	*H. martiusii*	Brazil	Ceará	25046	Abietane diterpene	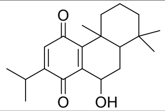	7ß-hydroxy-11,14-dioxoabieta-8,12-diene	$C_{20}H_{28}O_3$	N/A	316.2038
Lamiaceae	*H. martiusii*	Brazil	Ceará	25046	Abietane diterpene	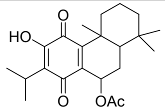	7α-acetoxy-royleanone	$C_{22}H_{30}O_5$	6812-88-0	374.2093
Lamiaceae	*H. martiusii*	Brazil	Ceará	25046	Abietane diterpene	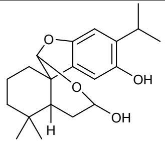	7-seco-7(20),11(20)-Diepoxy-7,14-dihydroxyabieta-8,11,13-triene	$C_{20}H_{28}O_4$	N/A	332.1988

Lamiaceae	*H. martiusii*	Brazil	Ceará	25046	Abietane diterpene	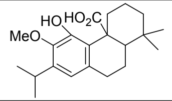	12-Methoxycarnosic acid	$C_{21}H_{30}O_4$	62201-71-2	346.2144
Lamiaceae	*H. martiusii*	Brazil	Ceará	25046	Abietane diterpene	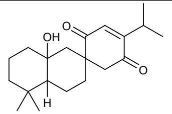	Martiusane	$C_{20}H_{30}O_3$	N/A	318.2195
Lamiaceae	*H. monticola*	Mexico	Veracruz	N/A	Furanone	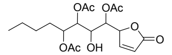	Monticofuranolide A	$C_{18}H_{26}O_9$	2738563-48-7	386.1577
Lamiaceae	*H. monticola*	Mexico	Veracruz	N/A	Flavonoid	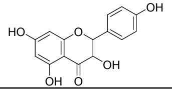	Dihydrokaempferol	$C_{15}H_{12}O_6$	480-20-6	288.0634
Lamiaceae	*H. monticola*	Mexico	Veracruz	N/A	Flavonoid	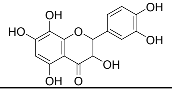	2,3-Dihydrogossypetin	$C_{15}H_{12}O_8$	40525-53-9	320.0532
Lamiaceae	*H. monticola*	Brazil	Minas Gerais	N/A	Flavonoid	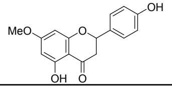	Sakuranetin	$C_{16}H_{14}O_5$	2957-21-3	286.0841
Lamiaceae	*H. monticola*	Brazil	Minas Gerais	N/A	Flavonoid	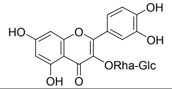	Rutin	$C_{27}H_{30}O_{16}$	153-18-4	610.1534
Lamiaceae	*H. monticola*	Brazil	Minas Gerais	N/A	Flavonoid	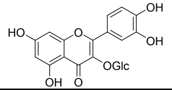	Hyperoside	$C_{21}H_{20}O_{12}$	482-36-0	464.0955
Lamiaceae	*H. monticola*	Brazil	Minas Gerais	N/A	Flavonoid	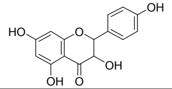	Aromadendrin	$C_{15}H_{12}O_6$	480-20-6	288.0634
Lamiaceae	*H. monticola*	Brazil	Minas Gerais	N/A	Flavonoid	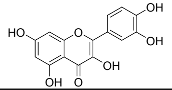	Quercetin	$C_{15}H_{10}O_7$	117-39-5	302.0427
Lamiaceae	*H. monticola*	Brazil	Minas Gerais	N/A	Flavonoid	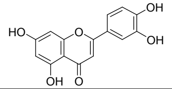	Luteolin	$C_{15}H_{10}O_6$	491-70-3	286.0477
Lamiaceae	*H. mutabilis*	Mexico	Veracruz	8520 M	Triterpene	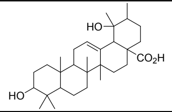	3α,19α-Dihydroxyurs-12-en-28-oic acid	$C_{30}H_{48}O_4$	N/A	472.3553

Lamiaceae	*H. mutabilis*	Mexico	Veracruz	8520 M	Triterpene	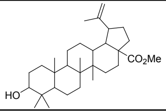	Betulinic Acid Methyl Ester	$C_{31}H_{50}O_3$	2259-06-5	470.3760
Lamiaceae	*H. mutabilis*	Mexico	Veracruz	8520 M	Triterpene	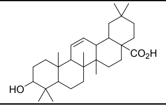	Oleanolic acid	$C_{30}H_{48}O_3$	508-02-1	456.3603
Lamiaceae	*H. mutabilis*	Mexico	Veracruz	8520 M	Triterpene	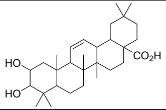	Maslinic acid	$C_{30}H_{48}O_4$	4373-41-5	472.3553
Lamiaceae	*H. mutabilis*	Mexico	Veracruz	8520 M	Triterpene	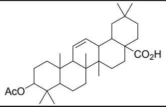	Oleanolic acetate	$C_{32}H_{50}O_4$	4339-72-4	498.3709
Lamiaceae	*H. oblongifolia*	Mexico	N/A	MEXU-10104-M	α-Pyrone	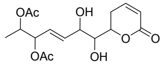	6-(1,2-Dihydroxy-5,6-diacetoxy-3-heptenyl)-5,6-dihydro-2H-pyran-2-one	$C_{16}H_{22}O_8$	N/A	342.1315
Lamiaceae	*H. oblongifolia*	Mexico	N/A	MEXU-10104-M	α-Pyrone	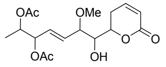	[(E)-3-acetyloxy-7-hydroxy-6-methoxy-7-(6-oxo-2,3-dihydropyran-2-yl)hept-4-en-2-yl] acetate	$C_{17}H_{24}O_8$	N/A	356.1471
Lamiaceae	*H. oblongifolia*	Mexico	N/A	MEXU-10104-M	Triterpene	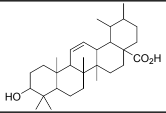	Ursolic acid	$C_{30}H_{48}O_3$	77-52-1	456.3603
Lamiaceae	*H. oblongifolia*	Mexico	N/A	MEXU-10104-M	Triterpene	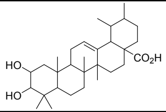	Corosolic Acid	$C_{30}H_{48}O_4$	4547-24-4	472.3553
Lamiaceae	*H. oblongifolia*	Mexico	N/A	MEXU-10104-M	α-Pyrone	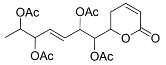	6R-[1R,2R,5R,6S-(tetracetyloxy)-3E-heptenyl]-5,6-dihydro-2H-pyran-2-one	$C_{20}H_{26}O_{10}$	N/A	426.1526
Lamiaceae	*H. pectinata*	Mexico	Veracruz	M-8153	α-Pyrone	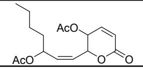	Pectinolide A	$C_{16}H_{22}O_6$	149155-54-4	310.1416

Lamiaceae	*H. pectinata*	Mexico	Veracruz	M-8153	α-Pyrone	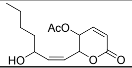	Pectinolide B	$C_{14}H_{20}O_5$	149155-55-5	268.1311
Lamiaceae	*H. pectinata*	Jamaica	Kingston	SC1804	Pyrone	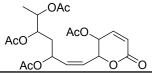	Pectinolide E	$C_{20}H_{26}O_{10}$	651329-18-9	426.1526
Lamiaceae	*H. pectinata*	Jamaica	Kingston	SC1804	Pyrone	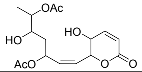	Pectinolide D	$C_{16}H_{22}O_8$	651329-17-8	342.1315
Lamiaceae	*H. pectinata*	Jamaica	Kingston	SC1804	Pyrone	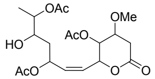	Pectinolide F	$C_{21}H_{30}O_{11}$	651329-19-0	458.1788
Lamiaceae	*H. pectinata*	Jamaica	Kingston	SC1804	Furanone	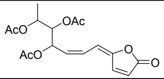	Pectinolide G	$C_{18}H_{22}O_8$	651329-20-3	366.1315
Lamiaceae	*H. pectinata*	Brazil	Rio de Janeiro	RFA399 27	Furanone	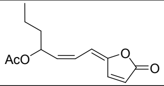	Pectinolide N	$C_{14}H_{18}O_4$	2738563-89-6	250.1205
Lamiaceae	*H. pectinata*	Brazil	Rio de Janeiro	RFA399 27	Pyrone	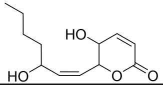	Pectinolide I	$C_{16}H_{22}O_6$	862509-97-5	310.1416
Lamiaceae	*H. pectinata*	Brazil	Rio de Janeiro	RFA399 27	Furanone	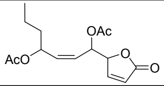	Pectinolide O	$C_{12}H_{18}O_4$	2738563-90-9	226.1205
Lamiaceae	*H. pectinata*	Brazil	Rio de Janeiro	RFA399 27	Furanone	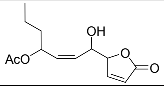	Pectinolide H	$C_{14}H_{20}O_5$	862509-94-2	268.1311
Lamiaceae	*H. pectinata*	Brazil	Rio de Janeiro	RFA399 27	Furanone	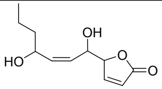	Pectinolide P	$C_{12}H_{18}O_4$	2738563-91-0	226.1205
Lamiaceae	*H. pectinata*	Mexico	Veracruz	XAL000 1406 and XAL000 0286	Flavonoid	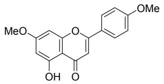	5-hydroxy-7,4′-dimethoxyflavone	$C_{17}H_{14}O_5$	5128-44-9	298.0841
Lamiaceae	*H. pectinata*	Mexico	Veracruz	XAL000 1406 and XAL000 0286	Flavonoid	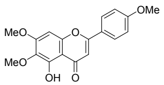	Salvigenin	$C_{18}H_{16}O_6$	19103-54-9	328.0947
Lamiaceae	*H. pectinata*	Brazil	Pernambuco	N/A	Phenylpropan oid	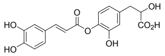	Sambacaitaric acid	$C_{18}H_{16}O_8$	N/A	360.0845

Lamiaceae	*H. pectinata*	Brazil	Pernambuco	N/A	Phenylpropanoid	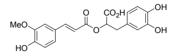	3-O-methyl-rosmarinic acid	$C_{19}H_{18}O_8$	N/A	374.1002
Lamiaceae	*H. pectinata*	Brazil	Pernambuco	N/A	Flavonoid	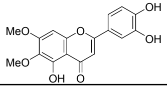	Cirsiliol	$C_{17}H_{14}O_7$	34334-69-5	344.0896
Lamiaceae	*H. pectinata*	Brazil	Pernambuco	N/A	Flavonoid	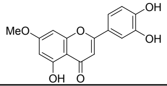	7-O-methylluteolin	$C_{16}H_{12}O_6$	20243-59-8	300.0634
Lamiaceae	*H. pectinata*	Brazil	Pernambuco	N/A	Flavonoid	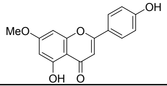	Genkwanin	$C_{16}H_{12}O_5$	437-64-9	284.0685
Lamiaceae	*H. platanifolia*	Brazil	Ceará	31674	Abietane diterpene	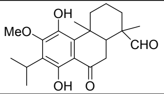	19-oxo-inuroyleanol	$C_{21}H_{28}O_5$	N/A	360.1937
Lamiaceae	*H. platanifolia*	Brazil	Ceará	31674	Abietane diterpene	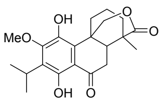	11,14-dihydroxy-12-methoxy-7-oxo-8,11,13-abietatrien-19,20 b-olide	$C_{21}H_{26}O_6$	N/A	374.1729
Lamiaceae	*H. platanifolia*	Brazil	Ceará	31674	Abietane diterpene	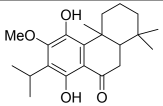	Inuroyleanol	$C_{21}H_{30}O_4$	57685-31-1	346.2144
Lamiaceae	*H. platanifolia*	Brazil	Ceará	31674	Steroid	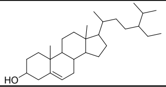	Sitosterol	$C_{29}H_{50}O$	83-46-5	414.3862
Lamiaceae	*H. platanifolia*	Brazil	Ceará	31674	Steroid	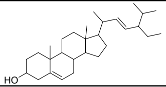	Stigmasterol	$C_{29}H_{48}O$	83-48-7	412.3705
Lamiaceae	*H. platanifolia*	Brazil	Ceará	31674	Triterpene	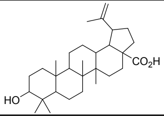	Betulinic acid	$C_{30}H_{48}O_3$	472-15-1	456.3603
Lamiaceae	*H. radicans*	N/A	N/A	N/A	Triterpene	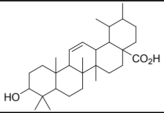	Ursolic acid	$C_{30}H_{48}O_3$	77-52-1	456.3603
Lamiaceae	*H. radicans*	N/A	N/A	N/A	Steroid	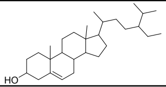	Sitosterol	$C_{29}H_{50}O$	83-46-5	414.3862

Lamiaceae	*H. radicans*	Brazil	São Paulo	Silva-Luz 295	Phenolic acid	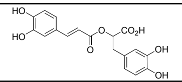	Rosmarinic acid	$C_{18}H_{16}O_8$	20283-92-5	360.0845
Lamiaceae	*H. rhomboidea*	Taiwan	Taipei	SPNTU2011HR05	Phenylpropanoid	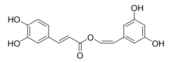	Nepetoidin A	$C_{17}H_{14}O_6$	130233-90-8	314.0790
Lamiaceae	*H. rhomboidea*	Taiwan	Taipei	SPNTU2011HR05	Flavonoid	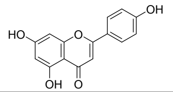	Apigenin	$C_{15}H_{10}O_5$	520-36-5	270.0528
Lamiaceae	*H. rhomboidea*	Taiwan	Taipei	SPNTU2011HR05	Flavonoid	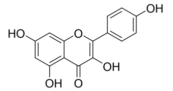	Kaempferol	$C_{15}H_{10}O_6$	520-18-3	286.0477
Lamiaceae	*H. rhomboidea*	Taiwan	Taipei	SPNTU2011HR05	Phenylpropanoid	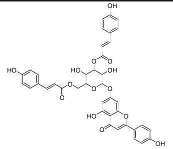	Anisofolin A	$C_{39}H_{32}O_{14}$	83529-71-9	724.1792
Lamiaceae	*H. rhomboidea*	Taiwan	Taipei	SPNTU2011HR05	Flavonoid	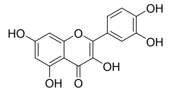	Quercetin	$C_{15}H_{10}O_7$	117-39-5	302.0427
Lamiaceae	*H. rhomboidea*	Taiwan	Taipei	SPNTU2011HR05	Phenylpropanoid	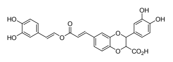	(2S,3S)-3-(3,4-dihydroxyphenyl)-6-((E)-3-(3,4-dihydroxystyryloxy)-3-oxoprop-1-enyl)-2,3-dihydrobenzo[b][1,4]dioxine-2-carboxylic acid	$C_{26}H_{20}O_{10}$	N/A	492.1056
Lamiaceae	*H. rhomboidea*	Taiwan	Taipei	SPNTU2011HR05	Phenylpropanoid	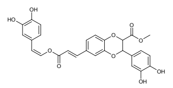	(2S,3S)-methyl 3-(3,4-dihydroxyphenyl)-6-((Z)-3-(3,4-dihydroxystyryloxy)-3-oxoprop-1 -enyl)-2,3-dihydrobenzo[b][1,4]dioxine-2-carboxylate	$C_{27}H_{22}O_{10}$	N/A	506.1213
Lamiaceae	*H. salzmanii*	Brazil	Pernambuco	HS5420	Lignan	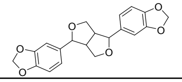	(+)-Sesamin	$C_{20}H_{18}O_6$	607-80-7	354.1103

Lamiaceae	*H. salzmanii*	Brazil	Pernambuco	HS5420	Lignan	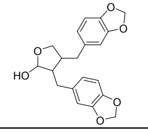	(-)-Cubebin	$C_{20}H_{20}O_6$	18423-69-3	356.1260
Lamiaceae	*H. salzmanii*	Brazil	Pernambuco	HS5420	Chalcone	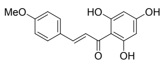	2',4',6'-Trihydroxy-4-methoxychalcone	$C_{16}H_{14}O_5$	56927-09-4	286.0841
Lamiaceae	*H. salzmanii*	Brazil	Pernambuco	HS5420	Isopimarane diterpene	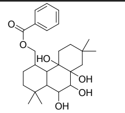	(−)-Salzol	$C_{27}H_{38}O_6$	126214-17-3	458.2668
Lamiaceae	*H. salzmanii*	Brazil	Pernambuco	HS5420	Flavonoid	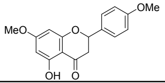	4',7-Di-O-methylnaringenin	$C_{17}H_{16}O_5$	29424-96-2	300.0998
Lamiaceae	*H. salzmanii*	Brazil	Pernambuco	HS5420	Phenolic acid	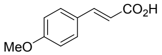	4-Methoxycinnamic acid	$C_{10}H_{10}O_3$	830-09-1	178.0630
Lamiaceae	*H. spicigera*	Mexico	Veracruz	M-21851	Labdane diterpene	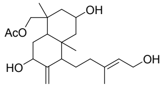	19-acetoxy-2α,7α,15-trihydroxylabda-8(17),(13Z)-diene	$C_{21}H_{34}O_5$	219561-53-2	366.2406
Lamiaceae	*H. spicigera*	Mexico	Veracruz	M-21851	Labdane diterpene	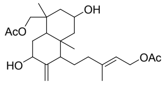	15,19-diacetoxy-2α,7α-dihydroxylabda-8(17),(13Z)-diene	$C_{23}H_{36}O_6$	219561-64-5	408.2512
Lamiaceae	*H. spicigera*	Mexico	Veracruz	M-21851	Labdane diterpene	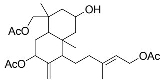	7α,15,19-triacetoxy-2α-hydroxylabda-8(17),(13Z)-diene	$C_{25}H_{38}O_7$	219561-75-8	450.2618
Lamiaceae	*H. spicigera*	Mexico	Veracruz	M-21851	Labdane diterpene	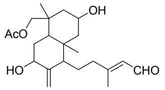	19-acetoxy-2α,7α-dihydroxylabda-8(17),(13Z)-dien-15-al	$C_{21}H_{32}O_5$	219561-83-8	364.2250
Lamiaceae	*H. spicigera*	Mexico	Veracruz	M-21851	Labdane diterpene	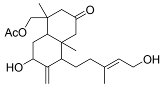	19-acetoxy-7α,15-dihydroxylabda-8(17),(13Z)-dien-2-one	$C_{22}H_{34}O_5$	219561-93-0	378.2406
Lamiaceae	*H. spicigera*	Mexico	Veracruz	M-21851	Kaurane diterpene	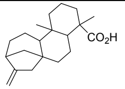	Kaurenoic acid	$C_{20}H_{30}O_2$	6730-83-2	302.2246
Lamiaceae	*H. spicigera*	Mexico	Veracruz	M-21851	Triterpene	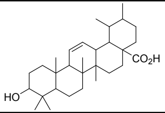	Ursolic acid	$C_{30}H_{48}O_3$	77-52-1	456.3603

Lamiaceae	*H. spicigera*	Mexico	Veracruz	M-21851	Labdane diterpene	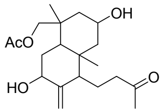	19-acetoxy-2α,7α-dihydroxylabda-14,15-dinorlabd-8(17)-en-13-one	$C_{20}H_{32}O_5$	219562-01-3	352.2250
Lamiaceae	*H. spicigera*	Mexico	Veracruz	M-21851	Labdane diterpene	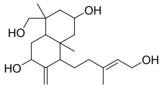	2α,7α,15,19-tetrahydroxy-ent-labda-8(17),(13Z)-diene	$C_{20}H_{34}O_4$	219562-08-0	338.2457
Lamiaceae	*H. suaveolens*	N/A	N/A	N/A	Polyphenolic	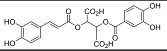	Chicoric acid	$C_{22}H_{18}O_{12}$	6537-80-0	474.0798
Lamiaceae	*H. suaveolens*	N/A	N/A	N/A	Polyphenolic	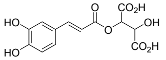	Caftaric acid	$C_{13}H_{12}O_9$	67879-58-7	312.0481
Lamiaceae	*H. suaveolens*	N/A	N/A	N/A	Phenolic acid	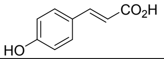	*p*-Coumaric acid	$C_9H_8O_3$	7400-08-0	164.0473
Lamiaceae	*H. suaveolens*	N/A	N/A	N/A	Phenolic acid	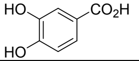	Protocatechuic acid	$C_7H_6O_4$	99-50-3	154.0266
Lamiaceae	*H. suaveolens*	N/A	N/A	N/A	Phenolic acid	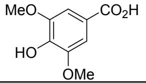	Syringic acid	$C_9H_{10}O_5$	530-57-4	198.0528
Lamiaceae	*H. suaveolens*	N/A	N/A	N/A	Phenolic acid	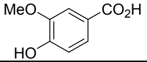	Vanillic acid	$C_8H_8O_4$	121-34-6	198.0528
Lamiaceae	*H. suaveolens*	Nigeria	N/A	EFH9010	Abietane diterpene	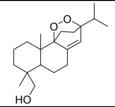	13alpha-epi-dioxiabiet-8(14)-en-18-ol	$C_{20}H_{32}O_3$	847165-11-1	320.2351
Lamiaceae	*H. suaveolens*	N/A	N/A	N/A	Phenolic ester	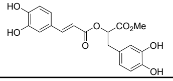	Methyl rosmarinate	$C_{19}H_{18}O_8$	99353-00-1	374.1002
Lamiaceae	*H. suaveolens*	India	Andhra Pradesh	NUH-NSP-003	Triterpene	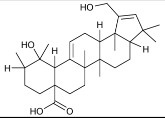	Hyptadienic Acid	$C_{30}H_{46}O_4$	128397-09-1	470.3396
Lamiaceae	*H. suaveolens*	N/A	N/A	N/A	Triterpene	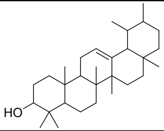	α-Amyrin	$C_{30}H_{50}O$	638-95-9	426.3862
Lamiaceae	*H. umbrosa*	Brazil	Pernambuco	5798	Quinone	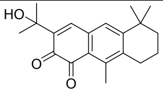	Umbrosone	$C_{20}H_{24}O_3$	131889-87-7	312.1725

Lamiaceae	*H. urticoides*	Mexico	Veracruz	N/A	Flavonoid	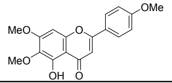	Salvigenin	$C_{18}H_{16}O_6$	19103-54-9	328.0947
Lamiaceae	*H. urticoides*	Mexico	Veracruz	N/A	Triterpene	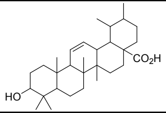	Ursolic acid	$C_{30}H_{48}O_3$	77-52-1	456.3603
Lamiaceae	*H. urticoides*	Mexico	Veracruz	N/A	Lactone	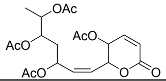	Hypurticin	$C_{20}H_{26}O_{10}$	651329-18-9	426.1526
Lamiaceae	*H. verticilata*	Mexico	Oaxaca	MEXU-431167	Lignan	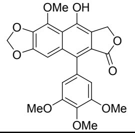	5-Methoxydehydropodophyllotoxin	$C_{23}H_{20}O_9$	N/A	440.1107
Lamiaceae	*H. verticilata*	Mexico	Oaxaca	MEXU-431167	Lignan	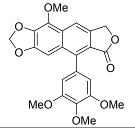	Dehydro-β-peltatin methyl ether	$C_{23}H_{20}O_8$	N/A	424.1158
Lamiaceae	*H. verticilata*	Mexico	Oaxaca	MEXU-431167	Lignan	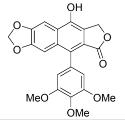	Dehydropodophyllotoxin	$C_{22}H_{18}O_8$	42123-27-3	410.1002
Lamiaceae	*H. verticilata*	Mexico	Oaxaca	MEXU-431167	Lignan	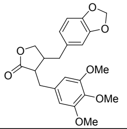	Yatein	$C_{22}H_{24}O_7$	40456-50-6	400.1522
Lamiaceae	*H. verticilata*	Mexico	Oaxaca	MEXU-431167	Lignan	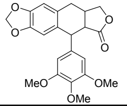	Deoxypicropodophyllotoxin	$C_{22}H_{22}O_7$	24150-39-8	398.1366
Lamiaceae	*H. verticilata*	Mexico	Oaxaca	MEXU-431167	Lignan	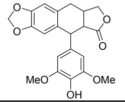	4'-Demethyldeoxypodophyllotoxin	$C_{21}H_{20}O_7$	3590-93-0	384.1209
Lamiaceae	*H. verticilata*	Mexico	Oaxaca	MEXU-431167	Lignan	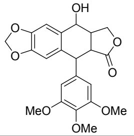	(+)-Podophyllotoxin	$C_{22}H_{22}O_8$	620622-69-7	414.1315

Lamiaceae	*H. verticilata*	N/A	N/A	N/A	Lignan	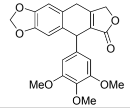	β-apopicropodophyllin	$C_{22}H_{20}O_7$	477-52-1	396.1209
Lamiaceae	*H. verticilata*	N/A	N/A	N/A	Lignan	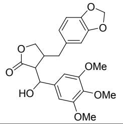	Epipodorhizol	$C_{22}H_{24}O_8$	17187-82-5	416.1471
Lamiaceae	*H. verticilata*	N/A	N/A	N/A	Lignan	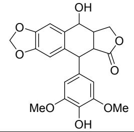	4'-Demethylpodophyllotoxin	$C_{21}H_{20}O_8$	40505-27-9	400.1158
Lamiaceae	*H. verticilata*	N/A	N/A	N/A	Alkaloid	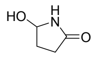	(R)-5-hydroxypyrrolidin-2-one	$C_4H_7NO_2$	168111-92-0	101.0477
Lamiaceae	*H. verticilata*	N/A	N/A	N/A	Monoterpene	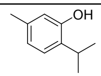	Thymol	$C_{10}H_{14}O$	89-83-8	150.1045
Lamiaceae	*H. verticilata*	N/A	N/A	N/A	Sesquiterpene	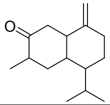	Cadina-10(15)-en-3-one	$C_{14}H_{22}O$	N/A	206.1671
Lamiaceae	*H. verticilata*	N/A	N/A	N/A	Sesquiterpene	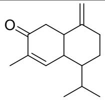	Cadina-4,10(15)-dien-3-one	$C_{15}H_{22}O$	131417-74-8	218.1671
Lamiaceae	*H. verticilata*	N/A	N/A	N/A	Diterpene	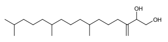	7,11,15-trimethyl-3-methylenehexadecane-1,2-diol	$C_{20}H_{40}O_2$	N/A	312.3028
Lamiaceae	*H. verticilata*	N/A	N/A	N/A	Diterpene	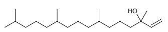	3,7,11,15-Tetramethylhexadec-1-en-3-ol	$C_{20}H_{40}O$	60046-87-9	296.3079
Lamiaceae	*H. verticilata*	N/A	N/A	N/A	Triterpene	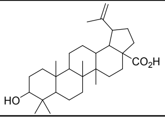	Betulinic acid	$C_{30}H_{48}O_3$	472-15-1	456.3603

Lamiaceae	*H. verticilata*	N/A	N/A	N/A	Triterpene	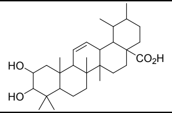	2α-hydroxyursolic acid	$C_{30}H_{48}O_4$	4547-24-4	472.3553
Lamiaceae	*H. verticilata*	N/A	N/A	N/A	Flavonoid	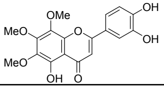	Sideritoflavone	$C_{18}H_{16}O_8$	70360-12-2	360.0845
Lamiaceae	*H. verticilata*	N/A	N/A	N/A	Abietane Diterpene	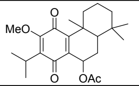	7-acetyl-12-methoxyhorminone	$C_{23}H_{32}O_5$	N/A	388.2250
Lamiaceae	*H. verticilata*	N/A	N/A	N/A	Abietane Diterpene	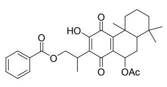	7-acetoxy-16-benzoxy-12-hydroxyabieta-8,12-diene-11,14-dione	$C_{29}H_{34}O_7$	1178557-95-3	494.2305
Lamiaceae	*H. verticilata*	N/A	N/A	N/A	Abietane Diterpene	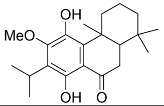	11,14 dihydroxy-12-methoxyabieta-8,11,13-triene-7-one	$C_{21}H_{30}O_4$	N/A	346.2144
Lamiaceae	*H. verticilata*	N/A	N/A	N/A	Abietane Diterpene	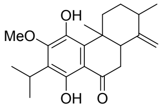	11,14-dihydroxy-12-methoxy- 18(4→3βH) abeo-abieta-4(19),8,11,13-tetraene-7-one	$C_{21}H_{28}O_4$	N/A	344.1988
Lamiaceae	*H. verticilata*	N/A	N/A	N/A	Abietane Diterpene	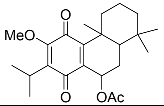	7-acetoxy-12-methoxyabieta-8,12-diene-11,14-dione	$C_{23}H_{32}O_5$	N/A	388.2250
Lamiaceae	*H. verticilata*	N/A	N/A	N/A	Abietane Diterpene	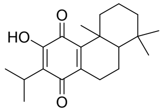	Royleanone	$C_{20}H_{28}O_3$	6812-87-9	316.2038
Lamiaceae	*H. verticilata*	N/A	N/A	N/A	Abietane Diterpene	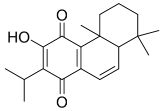	6,7-Dehydroroyleanone	$C_{20}H_{26}O_3$	6855-99-8	314.1882
Lamiaceae	*H. verticillata*	Jamaica	Kingston	N/A	Lignan	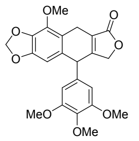	4-methoxy-9-(3,4,5-trimethoxyphenyl)-8,9-dihydrofuro[3’,4’:6,7]naphtho[2,3-	$C_{23}H_{22}O_8$	N/A	426.1315

							d][1,3]dioxol-6(5H)-one			
Lamiaceae	*H. verticillata*	Nigeria	Cross River State	N/A	Cyclic nitrosamine	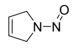	1H-pyrrole,2,5-dihydro-1-niroso-	$C_4H_6N_2O$	N/A	98.0480
Lamiaceae	*H. verticillata*	Nigeria	Cross River State	N/A	Hydrocarbons	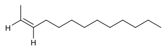	*cis*-2-Tridecene	$C_{13}H_{26}$	41446-59-7	182.2035
Lamiaceae	*H. verticillata*	Nigeria	Cross River State	N/A	Hydrocarbons		1-Pentadecene	$C_{15}H_{30}$	13360-61-7	210.2348
Lamiaceae	*H. verticillata*	Nigeria	Cross River State	N/A	Fatty acid ester	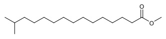	Methyl 14-methylpentadecanoate	$C_{17}H_{34}O_2$	5129-60-2	270.2559
Lamiaceae	*H. verticillata*	Nigeria	Cross River State	N/A	Hydrocarbons		1-Hexadecene	$C_{16}H_{32}$	629-73-2	224.2504
Lamiaceae	*H. verticillata*	Nigeria	Cross River State	N/A	Hydrocarbons		Hexadecane	$C_{16}H_{34}$	544-76-3	226.2661
Lamiaceae	*H. verticillata*	Nigeria	Cross River State	N/A	Fatty acid ester	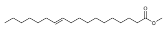	11-Octadecenoic acid, methyl ester	$C_{19}H_{36}O_2$	52380-33-3	296.2715
Lamiaceae	*H. verticillata*	Germany	Freiburg Botanical Garden	1277	Alkaloid	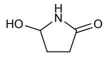	(R)-5-hydroxypyrrolidin-2-one	$C_4H_7NO_2$	168111-92-0	101.0477
Lamiaceae	*H. fruticosa*	N/A	N/A	N/A	Abietane diterpene	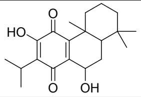	Horminone	$C_{20}H_{28}O_4$	21887-01-4	332.1988
Lamiaceae	*H. fruticosa*	N/A	N/A	N/A	Abietane diterpene	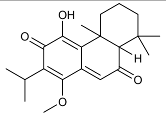	14-Methoxytaxodione	$C_{21}H_{32}O_4$	60371-71-3	348.2301
Lamiaceae	*H. fruticosa*	N/A	N/A	N/A	Abietane diterpene	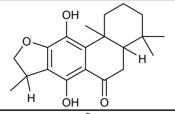	Hyptol	$C_{20}H_{26}O_4$	65563-54-4	330.1831
Lamiaceae	*H. heterodon*	Brazil	Rio Grande do Sul	Bordignon 4875	Kaurane diterpene	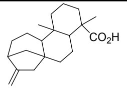	Kaurenoic acid	$C_{20}H_{30}O_2$	6730-83-2	302.2246
Lamiaceae	*H. suaveolens*	India	N/A	N/A	Triterpene	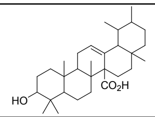	α-Peltoboykinolic acid	$C_{30}H_{48}O_3$	24778-49-2	456.3603

Lamiaceae	*H. suaveolens*	India	N/A	N/A	Triterpene	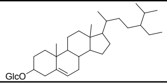	Sitosterol-β-D-glucoside	$C_{35}H_{60}O_6$	N/A	576.4390
Lamiaceae	*H. suaveolens*	India	N/A	N/A	Triterpene	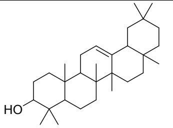	β-Amyrin	$C_{30}H_{50}O$	559-70-6	426.3862
Lamiaceae	*H. suaveolens*	India	N/A	N/A	Triterpene	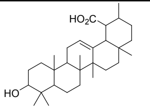	urs-12-en-3-β-ol-29-oic acid	$C_{30}H_{48}O_3$	N/A	456.3603
Lamiaceae	*H. urticoides*	Mexico	N/A	N/A	Triterpene	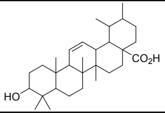	Ursolic acid	$C_{30}H_{48}O_3$	77-52-1	456.3603
Lamiaceae	*H. radicans*	Brazil	N/A	N/A	Triterpene	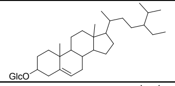	Sitosterol-β-D-glucoside	$C_{35}H_{60}O_6$	N/A	576.4390
Lamiaceae	*H. radicans*	Brazil	N/A	N/A	Triterpene	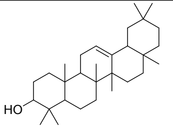	β-Amyrin	$C_{30}H_{50}O$	559-70-6	426.3862
Lamiaceae	*H. radicans*	Brazil	N/A	N/A	Triterpene	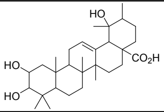	Tormentic acid	$C_{30}H_{48}O_5$	13850-16-3	488.3502
Lamiaceae	*H. radicans*	Brazil	N/A	N/A	Triterpene	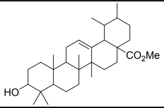	3β-Hydroxy-28-methyl-ursulate	$C_{31}H_{50}$O3	N/A	470.3760
Lamiaceae	*H. radicans*	Brazil	N/A	N/A	Triterpene	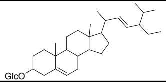	Stigmasterol-β-D-glucoside	$C_{35}H_{58}O_6$	N/A	574.4233
Lamiaceae	*H. fasciculata*	Brazil	N/A	N/A	Steroid	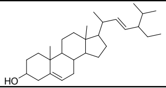	Stigmasterol	$C_{29}H_{48}O$	83-48-7	412.3705
Lamiaceae	*H. fasciculata*	Brazil	N/A	N/A	Steroid	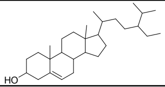	Sitosterol	$C_{29}H_{50}O$	83-46-5	414.3862

Lamiaceae	*H. fasciculata*	Brazil	N/A	N/A	Lignan	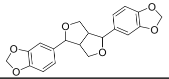	(+)-Sesamin	$C_{20}H_{18}O_6$	607-80-7	354.1103
Lamiaceae	*H. rhomboidea*	China	N/A	N/A	Lignan	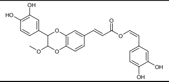	Hyprhombin B methyl ester	$C_{26}H_{22}O_9$	N/A	478.1264
Lamiaceae	*H. suaveolens*	China	N/A	N/A	Lignan	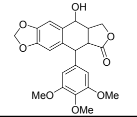	Podophyllotoxin	$C_{22}H_{22}O_8$	518-28-5	414.1315
Lamiaceae	*H. salzmannii*	Brazil	N/A	N/A	Lignan	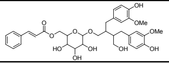	Hypenol	$C_{35}H_{42}O_{13}$	1447292-06-9	670.2625
Lamiaceae	*H. salzmannii*	Brazil	N/A	N/A	Lignan	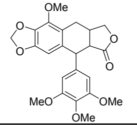	β-Peltatin-A methyl ether	$C_{23}H_{24}O_8$	N/A	428.1471
Lamiaceae	*H. pectinata*	Brazil	N/A	N/A	Pyrone	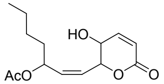	Pectinolide J	$C_{14}H_{20}O_5$	2307614-92-0	268.1311
Lamiaceae	*H. pectinata*	Brazil	N/A	N/A	Pyrone	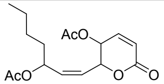	Pectinolide K	$C_{16}H_{22}O_6$	2307614-95-3	310.1416
Lamiaceae	*H. pectinata*	Brazil	N/A	N/A	Pyrone	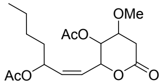	Pectinolide L	$C_{17}H_{24}O_7$	2307614-97-5	340.1522
Lamiaceae	*H. pectinata*	Brazil	N/A	N/A	Pyrone	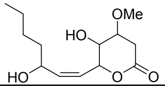	Pectinolide M	$C_{13}H_{20}O_5$	2307239-12-7	256.1311
Lamiaceae	*H. oblongifolia*	Mexico	N/A	N/A	α-Pyrone	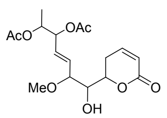	6R-[5R,6S-(diacetyloxy)-1R-(hydroxy)-2R-(methoxy)-3E-heptenyl]-5,6-dihydro-2H-pyran-2-one	$C_{16}H_{22}O_8$	N/A	342.1315
Lamiaceae	*H. oblongifolia*	Mexico	N/A	N/A	α-Pyrone	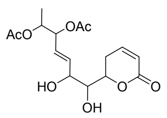	6R-[5R,6S-(diacetyloxy)-1S,2R)-(dihydroxy)-3E-heptenyl]-5,6-dihydro-2H-pyran-2-one	$C_{17}H_{24}O_8$	N/A	356.1417

Lamiaceae	*H.monticola*	Brazil	N/A	N/A	Pyrone	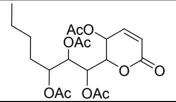	Monticolide A	$C_{20}H_{28}O_{10}$	149155-60-2	428.1682
Lamiaceae	*H.monticola*	Brazil	N/A	N/A	Pyrone	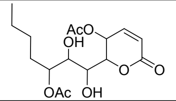	Monticolide B	$C_{16}H_{24}O_{8}$	149155-58-8	344.1471
Lamiaceae	*H. ovalifolia*	Brazil	Goiás	N/A	α-Pyrone	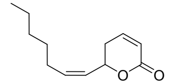	(R)-6-[1-heptenyl]-5,6-dihydro-2H-pyran	$C_{12}H_{18}O_{2}$	N/A	194.1307
Lamiaceae	*H. tomentosa*	N/A	N/A	N/A	Flavonoid	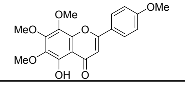	5-Hydroxy-4′,6,7,8-tetramethoxyflavone	$C_{19}H_{18}O_{7}$	2798-20-1	358.1053
Lamiaceae	*H. tomentosa*	N/A	N/A	N/A	Flavonoid	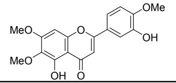	Eupatorin	$C_{18}H_{16}O_{7}$	855-96-9	344.0896
Lamiaceae	*H. suaveolens*	China	Guangdong	N/A	Flavonoid	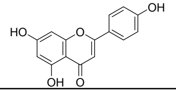	Apigenin	$C_{15}H_{10}O_{5}$	520-36-5	270.0528
Lamiaceae	*H. suaveolens*	China	Guangdong	N/A	Flavonoid	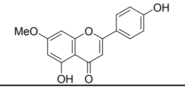	Genkwanin	$C_{16}H_{12}O_{5}$	437-64-9	284.0685
Lamiaceae	*H. suaveolens*	N/A	N/A	N/A	Flavonoid	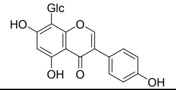	Genistein 8-C-Glucoside	$C_{21}H_{20}O_{10}$	66026-80-0	432.1056
Lamiaceae	*H. suaveolens*	China	Guangdong	N/A	Flavonoid	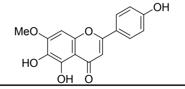	Sorbifolin	$C_{16}H_{12}O_{6}$	23130-22-5	300.0634
Lamiaceae	*H. brevipes*	Brazil	N/A	N/A	Flavonoid	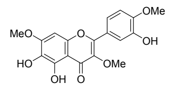	5,6,3′-trihydroxy-3,7,4′-trimethoxyflavone	$C_{18}H_{16}O_{8}$	548-74-3	360.0845
Lamiaceae	*H. brevipes*	Brazil	N/A	N/A	Flavonoid	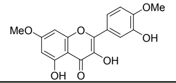	3,5,3′-trihydroxy-7,4′-dimethoxyflavone	$C_{17}H_{14}O_{7}$	529-40-8	330.0740
Lamiaceae	*H. brevipes*	Brazil	N/A	N/A	Flavonoid	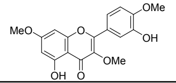	5,3′-Dihydroxy-3,7,4′-trimethoxyflavone	$C_{18}H_{16}O_{7}$	572-32-7	344.0896
Lamiaceae	*H. suaveolens*	N/A	N/A	N/A	Phenolic acid	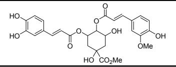	Methyl-3,4-dicaffeoylquinate	$C_{27}H_{28}O_{12}$	N/A	544.1581

Lamiaceae	*H.salzmannii*	Brazil	N/A	N/A	Phenolic acid	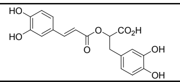	Rosmarinic acid	$C_{18}H_{16}O_8$	20283-92-5	360.0845
Lamiaceae	*H.salzmannii*	Brazil	N/A	N/A	Phenolic ester	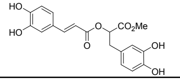	Methyl rosmarinate	$C_{19}H_{18}O_8$	99353-00-1	374.1002

N/A = Not applicable

References

Ahmad, V. U.; Rashid, M. A.; Abbasi, M. A.; Rasool, N.; Zubair, M. New Salirepin Derivatives from *Symplocos racemosa*. *J. Asian Nat. Prod. Res.* 2007, 9 (3–5), 209–215. DOI: 10.1080/10286020500531993.

Ali, A.; Ahmadi, F.; Cottrell, J. J.; Dunshea, F. R. Comprehensive Metabolite Fingerprinting of Australian Black and Green Olives and Their Antioxidant and Pharmacokinetics Properties. *Separations* **2023**, *10* (6), 354. DOI: 10.3390/separations10060354.

Araújo, E. C. C.; Lima, M. A. S.; Montenegro, R. C.; Nogueira, M.; Costa-Lotufo, L. V.; Pessoa, C.; Moraes, M. O. de; Silveira, E. R. Cytotoxic Abietane Diterpenes from *Hyptis martiusii* Benth. *Z. Naturforsch. C* **2006**, *61* (3–4), 177–183. DOI: 10.1515/znc-2006-3-404.S

Araújo, E. C. C.; Lima, M. A. S.; Montenegro, R. C.; Nogueira, M.; Costa-Lotufo, L. V.; Pessoa, C.; Moraes, M. O. de; Silveira, E. R. Spectral Assignments of New Diterpenes from *Hyptis martiusii* Benth. *Magn. Reson. Chem.* **2004**, *42* (3), 177–183. DOI: 10.1002/mrc.1489.

Asano, M.; Yamada, K.; Tanaka, T.; Matsuo, Y.; Kouno, I. New Bisabolane Sesquiterpene from the Mycelia of *Amanita virgineoides*. *Chem. Pharm. Bull.* **2013**, *61* (3), 366–369. DOI: 10.1248/cpb.c12-01070.

Astiti, M. A.; Jittmittraphap, A.; Leaungwutiwong, P.; Chutiwitoonchai, N.; Pripdeevech, P.; Mahidol, C.; Ruchirawat, S.; Kittakoop, P. LC-QTOF-MS/MS Based Molecular Networking Approach for the Isolation of α-Glucosidase Inhibitors and Virucidal Agents from *Coccinia grandis* (L.) Voigt. *Foods* **2021**, *10* (12), 3041. DOI: 10.3390/foods10123041.

Bhat, S. V.; Kalyanaraman, P. S.; Kohl, H.; de Souza, N. J.; Fehlhaber, H.-W. Inu-royleanol and 7-Ketoroyleanone, Two Novel Diterpenoids of *Inula royleana* DC. *Tetrahedron* **1975**, *31* (8), 1001–1005. DOI: 10.1016/0040-4020(75)80117-7.

Boalino, D. M.; Connolly, J. D.; McLean, S.; Reynolds, W. F.; Tinto, W. F. α-Pyrones and a 2(5H)-furanone from *Hyptis pectinata*. *Phytochemistry* **2003**, *64* (7), 1303–1307. DOI: 10.1016/j.phytochem.2003.08.017.

Brown, G. D.; Wong, H. F. Total Synthesis of (±)-Maculalactone A, Maculalactone B, and Maculalactone C and the Determination of the Absolute Configuration of Natural (+)-Maculalactone A by Asymmetric Synthesis. *Tetrahedron* **2004**, *60* (25), 5439–5451. DOI: 10.1016/j.tet.2004.04.060.

Chen, X.-L.; Luo, Q.-Y.; Hu, W.-Y.; Chen, J.-J.; Zhang, R.-P. Abietane Diterpenoids with Antioxidative Damage Activity from *Rosmarinus officinalis*. *J. Agric. Food Chem.* **2020**, *68* (20), 5631–5640. DOI: 10.1021/acs.jafc.0c01347

Da Silva, A. S.; Martínez-Fructuoso, L.; Simas, R. C.; Leitão, G. G.; Fragoso-Serrano, M.; Barros, Y. S.; de Souza, D. R.; Pereda-Miranda, R.; Leitão, S. G. Distribution of 5,6-Dihydro-α-Pyrones by Electrospray Ionization Ion Trap Mass Spectrometry in Different Aerial Parts of *Hyptis monticola*. *Phytochemistry* **2021**, *185*, 112706. DOI: 10.1016/j.phytochem.2021.112706.

Dauguet, J. C.; Bert, M.; Dolley, J.; Bekaert, A.; Lewin, G. 8-Methoxykaempferol 3-neohesperidoside and other flavonol glycosides from *Rosa rugosa* pollen. *Phytochemistry* **1993**, *33* (3), 503–506. DOI: 10.1016/0031-9422(93)85121-7.

Duan, H.; Wang, W.; Li, Y.; Khan, G. J.; Chen, Y.; Shen, T.; Bao, N.; Hua, J.; Xue, Z.; Zhai, K.; Wei, Z. Identification of phytochemicals and antioxidant activity of *Premna microphylla* Turcz. stem through UPLC-LTQ-Orbitrap-MS. *Food Chem.* **2022**, *373* (Pt B), 131482. DOI: 10.1016/j.foodchem.2021.131482.

Fang, Z.; et al. Capsofulvesins A–C, Cholinesterase Inhibitors from *Capsosiphon fulvescens*. *Chem. Pharm. Bull.* **2012**, *60* (11), 1351–1358. DOI: 10.1248/cpb.c12-00268.

Fragoso-Serrano, M.; González-Chávez, M. M.; Pereda-Miranda, R. Novel Labdane Diterpenes from the Insecticidal Plant *Hyptis spicigera*. *J. Nat. Prod.* **1999**, *62* (1), 45–50. DOI: 10.1021/np980222z.

Ge, H. M.; Yang, W. H.; Shen, Y.; Jiang, N.; Guo, Z. K.; Luo, Q.; Xu, Q.; Ma, J.; Tan, R. X. Immunosuppressive Resveratrol Aneuploids from *Hopea chinensis*. *Chem. – Eur. J.* **2010**, *16* (21), 6338-6345. DOI: 10.1002/chem.201000230.

Hensch, M.; Roussel, A.; Raynaud, J. Abietane diterpenoids from *Salvia candelabrum*. *Phytochemistry* **1975**, *14* (8), 1821–1824. DOI: 10.1016/0031-9422(75)85325-7.

Jewers, K.; Nagler, M. J.; Hussain, S. F.; Miana, G. The Identification of Acetylramosin C as Tetra-Acetylswertiamaroside. *Phytochemistry* **1975**, *14* (2), 297–298. DOI: 10.1016/0031-9422(75)85067-9.

Kitajima, J.; Suzuki, N.; Ishikawa, T.; Tanaka, Y. New Hemiterpenoid Pentol and Monoterpenoid Glycoside of *Torilis japonica* Fruit and Consideration of the Origin of Apiose. *Chem. Pharm. Bull.* **1998**, *46* (12), 1583–1586. DOI: 10.1248/cpb.46.1583.

Li, Y.-Y.; Chou, G.-X.; Wang, Z.-T. New Diarylheptanoids and Kavalactone from *Alpinia katsumadai* Hayata. *Helv. Chim. Acta* **2010**, *93* (2), 382–388. DOI: 10.1002/hlca.200900146.

Lima, K. S. B.; Guedes, M. L. S.; Silveira, E. R. Abietane Diterpenes from Hyptis crassifolia Mart. ex Benth. (Lamiaceae). J. Braz. Chem. Soc. 2015, 26 (1), 32–39. DOI: 10.5935/0103-5053.20140210.

Liu, G.; Zhuang, L.; Song, D.; Lu, C.; Xu, X. Isolation, Purification, and Identification of the Main Phenolic Compounds from Leaves of Celery (*Apium graveolens* L. var. *dulce* Mill./Pers.). *J. Sep. Sci.* **2017**, *40* (2), 472–479. DOI: 10.1002/jssc.201600995.

Liu, H.; Zheng, A.; Liu, H.; Yu, H.; Wu, X.; Xiao, C.; Dai, H.; Hao, F.; Zhang, L.; Wang, Y.; Tang, H. Identification of Three Novel Polyphenolic Compounds, Origanine A–C, with Unique Skeleton from *Origanum vulgare* L. Using the Hyphenated LC-DAD-SPE-NMR/MS Methods. *J. Agric. Food Chem.* **2012**, *60* (1), 129-135. DOI: 10.1021/jf204406u.

Miao, F.-P.; Liang, X.-R.; Liu, X.-H.; Ji, N.-Y. Aspewentins A-C, Norditerpenes from a Cryptic Pathway in an Algicolous Strain of *Aspergillus wentii*. *J. Nat. Prod.* **2014**, *77* (2), 429–432. DOI: 10.1021/np401047w.

Michavila, A.; Fernández-Gadea, F.; Rodríguez, B. Abietane diterpenoids from the root of *Salvia lavandulaefolia*. *Phytochemistry* **1986**, *25* (1), 266–268. DOI: 10.1016/S0031-9422(00)94547-3.

Mirzaei, H. H.; Firuzi, O.; Jassbi, A. R. Diterpenoids from Roots of *Salvia lachnocalyx*; In-silico and In-vitro Toxicity against Human Cancer Cell Lines. *Iran. J. Pharm. Res.* **2020**, *19* (4), 85–94. DOI: 10.22037/ijpr.2019.15429.13095.

Moore, B. S.; Chen, J.-L.; Patterson, G. M. L.; Moore, R. E. Structures of Cylindrocyclophanes A–F. *Tetrahedron* **1992**, *48* (15), 3001–3006. DOI: 10.1016/S0040-4020(01)92244-6.

Navarrete, A.; Katragunta, K.; Balderas-López, J. L.; Avula, B.; Khan, I. A. Chemical Profiling and Quantification of Flavones in Several *Pseudognaphalium* and *Gnaphalium* Species of Mexican Gordolobo Using UHPLC/PDA/MS. *J. Pharm. Biomed. Anal.* **2024**, *245*, 116186. DOI: 10.1016/j.jpba.2024.116186.

Nohara, T.; Kashiwada, Y.; Murakami, K.; Tomimatsu, T.; Kido, M.; Yagi, A.; Nishioka, I. Constituents of *Cinnamomi Cortex*. V. Structures of Five Novel Diterpenes, Cinncassiols D_1, D_1 Glucoside, D_2, D_2 Glucoside and D_3. *Chem. Pharm. Bull.* **1981**, *29* (9), 2451–2459. DOI: 10.1248/cpb.29.2451.

Obaroakpo, U. J.; Liu, L.; Zhang, S.; Jing, L.; Liu, L.; Pang, X.; Lv, J. Bioactive Assessment of the Antioxidative and Antidiabetic Activities of Oleanane Triterpenoid Isolates of Sprouted Quinoa Yoghurt Beverages and Their Anti-Angiogenic Effects on HUVECs Line. *J. Funct. Foods* **2020**, *66*, 103779. DOI: 10.1016/j.jff.2020.103779.

Pukalskas, A.; van Beek, T. A.; de Waard, P. Development of a triple hyphenated HPLC-radical scavenging detection-DAD-SPE-NMR system for the rapid identification of antioxidants in complex plant extracts. *J. Chromatogr. A* **2005**, *1074* (1-2), 81–88. DOI: 10.1016/j.chroma.2005.03.089.

Sabitha, G.; Raju, A.; Reddy, C. N.; Yadav, J. S. Stereoselective Total Synthesis of (+)-Hyptolide. *RSC Adv.* **2014**, *4* (3), 1496–1502. DOI: 10.1039/c3ra45042b.

Saidi, S.; Remok, F.; Handaq, N.; Drioiche, A.; Gourich, A. A.; Menyiy, N. E.; Amalich, S.; Elouardi, M.; Touijer, H.; Bouhrim, M.; Bouissane, L.; Nafidi, H.-A.; Bin Jardan, Y. A.; Bourhia, M.; Zair, T. Phytochemical Profile, Antioxidant, Antimicrobial, and Antidiabetic Activities of *Ajuga iva* (L.). *Life* **2023**, *13* (5), 1165. DOI: 10.3390/life13051165.

Wang, S.; Ma, D.; Hu, C. Three New Compounds from the Aerial Parts of *Caragana sinica*. *Helv. Chim. Acta* **2005**, *88* (11), 2315–2321. DOI: 10.1002/hlca.200590166.

Wang, X.; Shi, G.-R.; Liu, Y.-F.; Chen, R.-Y.; Yu, D.-Q. Four New Compounds from the Rhizome of *Aristolochia championii*. *J. Asian Nat. Prod. Res.* **2017**, *19* (2), 114–118. DOI: 10.1080/10286020.2016.1268129.

Wang, Y.; Shen, Y.-H.; Jin, H.-Z.; Fu, J.-J.; Hu, X.-J.; Qin, J.-J.; Liu, J.-H.; Chen, M.; Yan, S.-K.; Zhang, W.-D. Ainsliatrimers A and B, the First Two Guaianolide Trimers from *Ainsliaea fulvioides*. *Org. Lett.* **2008**, *10* (24), 5517–5520. DOI: 10.1021/ol802249z.

Yamagishi, T.; Zhang, D. C.; Chang, J. J.; McPhail, D. R.; McPhail, A. T.; Lee, K. H. The Cytotoxic Principles of *Hyptis capitata* and the Structures of the New Triterpenes Hyptatic Acid-A and -B. *Phytochemistry* **1988**, *27* (10), 3213–3216. DOI: 10.1016/0031-9422(88)80028-1.

Yaya, M.; Bessière, J.-M.; Dolmazon, R. Volatile Constituents of *Kyllinga erecta* S. *Bull. Chem. Soc. Ethiop.* **2001**, *15* (1), 71–78. DOI: 10.4314/bcse.v15i1.71732.

Yue, S.; Tang, Y.; Xu, C.; Li, S.; Zhu, Y.; Duan, J.-A. Two New Quinochalcone C-Glycosides from the Florets of *Carthamus tinctorius*. *Int. J. Mol. Sci.* **2014**, *15* (9), 16760–16771. DOI: 10.3390/ijms150916760.

Zhang, W.; Jiang, H.; Yang, J.; Jin, M.; Du, Y.; Sun, Q.; Cao, L.; Xu, H. Safety Assessment and Antioxidant Evaluation of Betulin by LC-MS Combined with Free-Radical Assays. *Anal. Biochem.* **2019**, *587*, 113460. DOI: 10.1016/j.ab.2019.113460.

Zhao, X.; Long, Z.; Dai, J.; Bi, K.; Chen, X. Identification of Multiple Constituents in the Traditional Chinese Medicine Formula Zhi-Zi-Chi Decoction and Rat Plasma after Oral Administration by Liquid Chromatography Coupled to Quadrupole Time-of-Flight Tandem Mass Spectrometry. *Rapid Commun. Mass Spectrom.* **2012**, *26* (20), 2443-2453. DOI: 10.1002/rcm.6360.

Zhu, H.; Bi, K.; Han, F.; Guan, J.; Zhang, X.; Mao, X.; Zhao, L.; Li, Q.; Hou, X.; Yin, R. Identification of the Absorbed Components and Metabolites of Zhi-Zi-Da-Huang Decoction in Rat Plasma by Ultra-High Performance Liquid Chromatography Coupled with Quadrupole-Time-of-Flight Mass Spectrometry. *J. Pharm. Biomed. Anal.* **2015**, *111*, 277–287. DOI: 10.1016/j.jpba.2015.03.043.
